# RECKLEEN is a lambda Red/CRISPR-Cas9 based single plasmid platform for enhanced genome editing in *Klebsiella pneumoniae*

**DOI:** 10.1038/s42003-025-08934-8

**Published:** 2025-10-30

**Authors:** Eslam M. Elsayed, Daniel Stukenberg, Doreen Meier, Bernd Schmeck, Anke Becker

**Affiliations:** 1 Center for Synthetic Microbiology (SYNMIKRO), Philipps-Universität Marburg, Marburg, Germany; 2 Department of Biology, Philipps-Universität Marburg, Marburg, Germany; 3 Department of Microbiology and Immunology, Faculty of Pharmacy, Zagazig University, Zagazig, Egypt; 4 Institute for Lung Research, Universities of Giessen and Marburg Lung Center, German Center for Lung Research (DZL), Philipps-University Marburg, Marburg, Germany; 5 Department of Medicine, Pulmonary and Critical Care Medicine, University Medical Center Marburg, Universities of Giessen and Marburg Lung Center, Philipps-University Marburg, Marburg, Germany; 6 Present Address: Department of Biology, Technical University Darmstadt, Darmstadt, Germany

**Keywords:** Microbiology techniques, Bacterial genetics, Synthetic biology

## Abstract

*Klebsiella pneumoniae* (*Kp*) has evolved as a major public health threat due to its multidrug-resistance (MDR) and hypervirulence. Current *Kp* genome-editing tools are constrained by cumbersome workflows, low flexibility, and limited scalability. Here, we present the RECKLEEN system —**Re**combineering/**C**RISPR-based ***KL****ebsiella* **E**ngineering for **E**fficient **N**ucleotide editing — as a single plasmid platform designed for precise genetic manipulation of *Kp*. RECKLEEN combines lambda Red recombineering with powerful CRISPR-Cas9-based targeted counterselection, achieving up to 99.998% killing efficiency. By implementing the near PAM-less SpG Cas9 variant in RECKLEEN, the compatible target sequence spectrum was significantly broadened. This approach enables deletions, point mutations, and DNA integrations, with efficiencies reaching 100% of the counter-selected clones. Simultaneous multi-target deletions were accomplished with up to 72% efficiency. To streamline the process, we developed a toolbox of eleven plasmids based on a modular cloning standard, enabling time- and resource-efficient assembly of editing constructs. This allows a 5-days workflow, from plasmid construction to the generation of strains with the desired genetic modification(s). The efficacy of RECKLEEN extends to various MDR *Kp* strains, such as ATCC 700721, ATCC BAA-1705, and ATCC 700603, demonstrating its broad applicability. RECKLEEN significantly enhances genome-editing capabilities for *Kp*, advancing research into its pathology and MDR mechanisms.

## Abstract (web summary)

RECKLEEN, a CRISPR–recombineering platform, enables efficient, flexible, and scalable genome editing in multidrug-resistant *Klebsiella pneumoniae*, facilitating precise deletions, point mutations, and integrations across diverse clinical strains.

## Introduction

*Klebsiella pneumoniae* (*Kp*), the “K” in the ESKAPE pathogens, is a Gram-negative bacterium that is now a common cause of both community- and hospital-acquired infections, including pneumonia, sepsis, and urinary tract infections^1,2^. In the last few decades, the evolution of *Kp* has led to the combination of hypervirulence and antimicrobial multi-drug resistance (MDR)^3,4^. These strains possess numerous virulence factors, such as their unique capsule, siderophores, fimbriae, efflux pumps, and several MDR genes^5^. Of particular concern is that *Kp* readily acquires mechanisms conferring resistance to a wide range of antimicrobial agents, including antibiotics of last resort such as carbapenems, tigecyclines, and polymyxins^1,3,6^. This enhances its virulence, survival in diverse environments, and dramatically limits the therapeutic options^5,7^. A better understanding of the pathology of *Kp* is needed to combat this public health threat. A time- and output-efficient genome editing tool applicable to MDR *Kp* strains would accelerate this basic research and enable new research approaches.

So far, both MDR and virulence of *Kp* limit the applicability of the available genome manipulation tools^1,8^. Conventional tools use multiple plasmids and require the integration of antibiotic resistance cassettes into the chromosome for selection. In most cases, this makes them unsuitable for genome editing of MDR strains^9–14^ as the resistance markers that can be used in the editing process are becoming increasingly limited due to the increasing MDR spectrum of *Kp* strains^1,3,13^. In addition, constructing the mutation and then removing the resistance marker is laborious and time-consuming^12,15^. Furthermore, the editing process leaves scars in the chromosome that limit the design space of the genetic manipulation and may affect genome stability^13,16^.

Synthetic biology has developed several advanced molecular biology tools based on components evolved from the interaction and the warfare between bacteria and phages^17–19^. The Red system, derived from the lambda (**λ**) Red bacteriophage with its main components Gam, Exo, and Beta, can integrate single-stranded DNA (ssDNA) or double-stranded DNA (dsDNA) into the genome via homologous recombination^9^. The donor DNA requires only ~50 nucleotides of homology to the target site to recombine^20–22^. Gam inhibits endogenous nucleases, protecting linear DNA from degradation. Exo, a 5′→3′ exonuclease, degrades linear dsDNA to produce single-stranded 3′ overhangs, which are then protected and annealed to complementary ssDNA by Beta^9,12^.

Similarly, CRISPR (clustered regularly interspaced short palindromic repeats)-Cas-based genome editing and gene regulation tools have been derived from a bacterial adaptive immune mechanism against phages^18,23,24^. CRISPR-Cas9 (CRISPR-associated protein 9) systems consist of two main components: the Cas9 endonuclease and a customizable synthetic guide RNA (sgRNA)^25,26^. Cas9 can be precisely directed to a specific DNA sequence through the flexible design of the spacer sequence in the *sgRNA*. At its programmed target site, Cas9 induces a double-strand break (DSB) adjacent to a 3 bp sequence on the target DNA called the protospacer adjacent motif (PAM, e.g., 5′-NGG-3′ for type II CRISPR-Cas9 of *Streptococcus pyogenes*)^25^. Many engineered Cas9 variants, such as SpG and SpRY, have expanded the targeting range to 5′-NGN-3′ PAMs, allowing broader applicability in therapeutic and gene-editing applications^26–28^.

CRISPR-Cas9 systems are now widely employed in bacteria to select against the wild-type (WT) sequence after targeted sequence modification^20,22,29^. This new counterselection method replaces conventional selection methods based on the insertion of an antibiotic resistance cassette into the chromosome^21,29^ or conditionally lethal markers, such as levansucrase^30^. An sgRNA-directed, Cas9-mediated DSB is lethal in many Gammaproteobacteria, including *Kp*, as they lack non-homologous end joining (NHEJ) mechanisms^8,15^. By specifically targeting the genome sequence of the non-edited cells, efficient recovery of the edited cells is possible. Thus, this counterselection provides a flexible method to distinguish different types of cells depending on their genome sequence^8,21,22^.

Lambda Red recombineering and CRISPR-Cas9 systems have previously been used for genome editing in *Kp*^8,15,31^. Wang et al. introduced a two-plasmid system and demonstrated its use for targeted gene deletions and integrations^15^. However, relying on the coordinated delivery and maintenance of two plasmids in *Kp* is complicated by low transformation efficiencies^32,33^ and MDR of this pathogen^5,6^. McConville et al. reported a single-plasmid system incorporating all components, and using the P_BAD_ promoter to synchronously induce expression of the *cas9* and lambda Red genes^8^. In this study, a 14-days protocol from plasmid construction to the genetically engineered strain was established and demonstrated for a gene deletion, with seven days required for genetic target-specific plasmid construction as a major time-consuming step. Both genome editing systems were a great step forward in the abilities to edit the *Kp* genome.

The aim of our study was to devise a lambda Red recombineering/CRISPR-Cas9-based counterselection genome editing system with improved efficiency (rate of positive clones, workload, and time required) and design flexibility that allows generating targeted deletions, integrations, and point mutations as well as multiplexing of editing targets. To this end, we pursued the following approaches: (i) stepwise control of lambda Red operon and *cas9/sgRNA* expression to enable sequential induction of recombination and counterselection, (ii) effective inhibition of Cas9 in phases counterselection is not desired, (iii) optimization of expression levels of all system components, (iv) expansion of the target spectrum by using a Cas9 variant with broader PAM acceptance range to achieve more flexibility for the design of point mutations and DNA integrations, and (v) a single plasmid platform in a modular cloning standard to shorten the genetic construction phase as well as enhance the scalability and flexibility of the design.

Here, we report the RECKLEEN system (**Re**combineering/**C**RISPR-based ***KL****ebsiella* **E**ngineering for **E**fficient **N**ucleotide editing) − a single plasmid platform, based on the common MoClo (modular cloning) standard, for genome editing of *Kp*. We demonstrate that RECKLEEN enables efficient, precise, markerless, and scarless genetic manipulation of *Kp*, with high design flexibility and achieving efficiencies of up to 100% for single deletions, point mutations, and DNA integrations, and up to 72% for simultaneous multi-target deletions of the surviving counter-selected clones. We introduce a workflow of only five days from the production of the editing constructs to the *Kp* strain with the desired genetic modification(s). Moreover, we show that the efficacy of RECKLEEN extends to various MDR *Kp* strains, demonstrating its broad applicability. We consider this to be a significant advancement in the ability to precisely edit the genome of MDR *Kp* strains, and thus in the technical basis for studying the molecular mechanisms underlying *Kp* pathology and resistance.

## Results and discussion

### Establishment of the RECKLEEN system as a lambda Red/CRISPR single-plasmid platform in *Kp*

#### Design of the RECKLEEN system

To establish a fast and efficient genome editing tool in *Kp*, we combined the potential of the recombineering system derived from the lambda Red bacteriophage^9,12^ with the powerful CRISPR-Cas9 system for counterselection^21,29^ in a single plasmid platform. The RECKLEEN plasmid was designed to enable a two-step workflow, where in step 1 the genome is edited through *Kp* cell transformation with short linear editing DNA and lambda Red-mediated homologous recombination, and in step 2 the cells with WT sequence are killed by induced production of Cas9/sgRNA. We employed parts from the Marburg collection^34^ to design the initial RECKLEEN plasmid vector containing two cassettes, one encoding the lambda Red and one the CRISPR-Cas9 system. The lambda Red operon (*gam, exo*, *beta*), as well as *cas9* and *sgRNA* (encoding a synthetic guide RNA combining tracrRNA and crRNA) were placed under the control of the isopropyl β-D-1-thiogalactopyranoside (IPTG)-inducible *P*_*tac*_ and the anhydrotetracycline (ATc)-inducible *P*_*tet*_ promoters, respectively. The vector backbone carries the *lacI* and *tetR* genes encoding the transcription regulators of these promoters, an apramycin resistance marker (*aac(3)-IV*) encoding an aminoglycoside 3-N-acetyltransferase type-IV enzyme for positive selection, and an pMB1 origin of replication. This plasmid was designed to be easily customizable for specific *sgRNA*-mediated sequence targeting. For this purpose, the plasmid contains a *sfgfp* transcription unit as dropout element upstream of the *sgRNA* scaffold sequence. This *sfgfp* can be replaced against a specific 20-nt guide spacer sequence by Golden Gate assembly^35,36^ using the type IIs restriction enzyme BsaI to generate a customized functional *sgRNA* gene. The initial RECKLEEN plasmid design is depicted in Supplementary Fig. 1A.

#### Anti-CRISPR mitigates the lethal effect of the CRISPR-Cas9 part of the RECKLEEN system

We observed that under non-inducing conditions (absence of ATc), presence of the initial RECKLEEN plasmid carrying *cas9* and a gene encoding an *sgRNA* targeting a chromosomal sequence in *Kp* MGH78578 (ATCC 700721) cells was a major challenge. We assembled four different plasmids that contained complete *sgRNA* genes, two with 20-nt chromosome-targeting sequences (5′ GGGTATAGCTGTAATTGGTT 3′ and 5′ GCGGAGATCTGGTACTGCCA 3′) in the coding sequence of *wzi*, encoding an outer membrane protein that anchors the capsular polysaccharide of *Kp*^37^, and two with control sequences (5′ GGATGGCGTGGGACGCGGCG 3′ and 5′ ATAAAGAAACTGTTACCCGT 3′) which have no target in the *Kp* chromosome (Supplementary Tables 1 and 3). No plasmid-positive colonies were obtained after electroporating *Kp* with plasmids coding for *sgRNAs* that target chromosomal sequences, while an empty control plasmid and the plasmids encoding the non-targeting control *sgRNAs* yielded tens of plasmid-positive colonies. It was likely that trace levels of Cas9 and chromosome-targeting sgRNA caused toxicity and cell death, possibly due to the introduction of the expected DNA DSB^8,21^. This could be explained with a basal activity of the *P*_*tet*_ promoters driving *cas9* and *sgRNA* expression even in absence of the inducer^38,39^. This suggests that tight control of Cas9 and sgRNA levels is necessary to avoid unintended killing of *Kp* cells.

We tested several strategies aimed at reducing the levels of Cas9 and sgRNA under non-inducing conditions (Supplementary Fig. 1B). These included (i) engineered variants of *P*_*tet*_ to reduce background activity of the *P*_*tet*_ promoter^34^, (ii) adding an SsrA consensus tag to the Cas9 C-terminus mediating degradation by the ClpP protease^21,40^, (iii) using alternative weaker ribosome binding sites (RBSs) to reduce the translation initiation rate of the *cas9* mRNA^34^, and (iv) reducing plasmid copy number by using different origins of replication (e.g., p15A and RSF1010). None of these interventions mitigated the observed toxicity under non-inducing conditions.

**Fig. 1:**
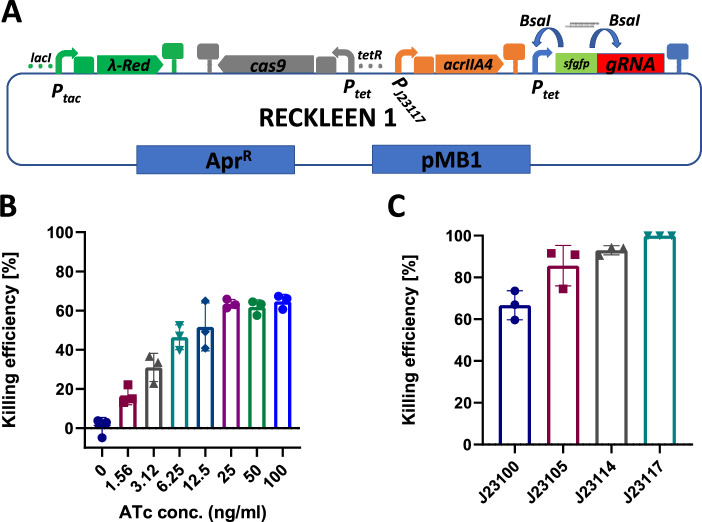
Design and optimization of the RECKLEEN system as a lambda Red/CRISPR-Cas9 single plasmid platform for efficient genome editing in *Kp*. **A** Schematic design of RECKLEEN 1 platform in *Kp*. The plasmid carries four different transcriptional units: the lambda Red operon (*gam, exo, beta*) under control of the inducible *P*_*tac*_ promoter, the *cas9* and *sgRNA* transcription units, both under control of the inducible *P*_*tet*_ promoter, as well as *P*_*J23117*_ *acrIIA4*. The vector backbone carries the *lacI* and *tetR* genes encoding the transcription regulators of these promoters, an apramycin resistance gene marker (*aac(3)-IV*), and an pMB1 origin of replication. This plasmid can be customized by replacing a *sfgfp* transcription unit in front of the sgRNA scaffold-encoding sequence with a 20-nt guide spacer sequence through Golden Gate assembly using the BsaI restriction enzyme. **B** Evaluation of the functionality of the Cas9/sgRNA part of the RECKLEEN system. The Cas9/sgRNA-mediated killing efficiency (percentage of cells killed upon induction of *P*_*tet*_ with different ATc concentrations) was determined. The killing efficiency was calculated as follows: ${Killing\; efficiency}\, [ \% ]=1-\frac{{CFU}/{mL\; in\; presence\; of\; inducer}({with\; counterselection})}{{CFU}/{mL\; in\; absence\; of\; inducer}({without\; counterselection})\,}\,$* 100. **C** Influence of constitutive promoters driving *acrIIA4* expression on the Cas9/sgRNA-mediated killing efficiency. The RECKLEEN 1 plasmid with *acrIIA4* under the control of constitutive *P*_*J23117*_ promoter achieved almost 100% killing efficiency. Data in B and C represent the mean of three biological replicates (*n* = 3), individual replicates are shown, and the error bars represent the standard deviation from the mean.

Lastly, we eliminated the hypothesized toxic effect of basal levels of Cas9 and chromosome-targeting sgRNA by co-production of the anti-CRISPR protein IIA4 (AcrIIA4). Anti-CRISPR proteins have been utilized in a variety of organisms to mitigate off-target effects and exert temporal and spatial control over CRISPR/Cas9 activity^29,41,42^. We included the *acrIIA4* transcription unit under the control of the constitutive J23100 promoter^34^ in the RECKLEEN 1 plasmid (Fig. 1A). Electroporation of *Kp* with this plasmid carrying the genes coding for the chromosome-targeting *sgRNAs* resulted in plasmid-positive colonies.

#### Optimization of the killing efficiency of the RECKLEEN system

Next, we evaluated Cas9/sgRNA-mediated killing efficiencies in the RECKLEEN system to assess its counterselection potential in *Kp*. We determined the effect of ATc concentration-dependent induction of the *P*_*tet*_ promoter, driving *cas9* and *sgRNA* expression, on the killing efficiency. RECKLEEN plasmid-containing cells, which target (5′ GGGTATAGCTGTAATTGGTT 3′) were cultured to an OD_600_ of 1, and the killing efficiency was determined after induction with ATc. We calculated the killing efficiency as follows: ${Killing\; efficiency}\left[ \% \right]=1-\frac{\frac{{CFU}}{{mL}}{in\; presence\; of\; inducer}\left({with\; counterselection}\right)}{\frac{{CFU}}{{mL}}{in\; absence\; of\; inducer}\left({without\; counterselection}\right)}\,* 100$. While the killing efficiency increased up to ~60% with increasing ATc concentrations up to 25 ng/mL (16.7 ± 3.9% at 1.5 ng/mL ATc and 63.4 ± 1.8% at 25 ng/mL ATc), further increases in ATc concentration up to 100 ng/mL did not significantly improve the killing efficiency (Fig. 1B). Based on these results, we selected 50 ng/mL ATc as an appropriate concentration for induction of the *P*_*tet*_ promoter for the expression of the *cas9* and *sgRNA*. Since more than a third of the cells survived the counterselection, further optimization of the gene expression of the RECKLEEN counterselection components was required to develop a highly efficient genome editing system.

The *acrIIA4* expression level was a starting point for optimization. The encoded anti-CRISPR protein buffers the Cas9 produced by leaky expression under non-inducing conditions. However, an excess of AcrIIA4 can reduce the maximum achievable amount of active Cas9 protein under inducing conditions^41–43^. To address this, we constructed a series of RECKLEEN plasmids that mediated varying *acrIIA4* expression levels through constitutive promoters of different strengths^34^. Specifically, the use of weaker promoters, such as *P*_*J23105*_, *P*_*J23114*_, and *P*_*J23117*_^34^, increased the killing efficiency to 85.6 ± 7.9%, 92.9 ± 1.8%, and 99.977 ± 0.01%, respectively (Fig. 1C). Thus, reducing *acrIIA4* expression significantly increased the killing efficiency. The parent plasmid construct using *P*_*J23117*_ for *acrIIA4* expression was designated as “RECKLEEN 1” (Fig. 1A). These results underscore that fine-tuning of the expression levels of *cas9*, *sgRNA*, and *acrIIA4* resulted in a reliable counterselection module in the RECKLEEN system, achieving near-complete elimination of *Kp* WT cells.

### Setting up a fast and convenient RECKLEEN-based genome editing workflow

**Fig. 2:**
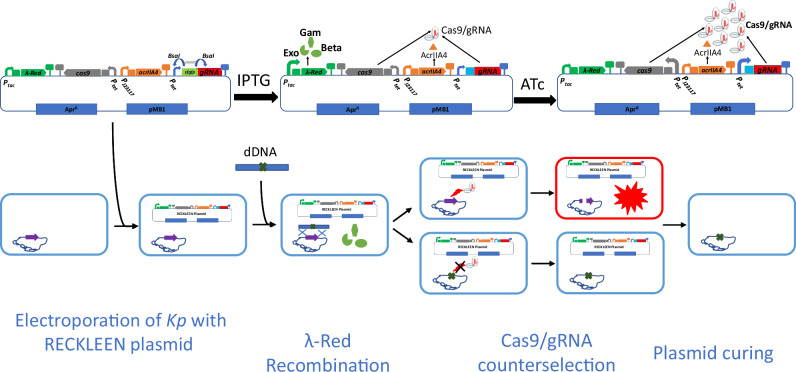
Schematic overview of the RECKLEEN system’s workflow. The RECKLEEN workflow started by inserting annealed oligonucleotides into the RECKLEEN plasmid, generating a 20-nt spacer sequence in the sgRNA that precisely targets the protospacer sequence in the target gene of *Kp* genome. This plasmid was then introduced into *Kp* by electroporation. Next, the recovered cells were cultured in presence of IPTG, to induce the *P*_*tac*_ promoter for the expression of the lambda Red operon genes. Then, these cells were employed to prepare electrocompetent cells. Subsequently, donor DNA (dDNA) template containing the homology arms for the target gene is then electroporated into these electrocompetent cells. The cells were then recovered in S.O.C. medium for 3 h. The recovered cells were then incubated overnight on plates containing the inducer ATc. During this period, some of the cells had the opportunity to undergo the recombination event, facilitated by the lambda Red components. Concurrently, ATc induces the *P*_*tet*_ promoter to overexpress the *cas9/sgRNA* components enabling the counterselection step by specifically targeting and cleaving the chromosome of the WT cells. Only cells that successfully underwent the recombination event were able to evade the lethal Cas9/sgRNA-induced DSB, allowing them to grow on the plates, as they no longer contained the target sequence. After confirming the accuracy of the edits, the RECKLEEN editing plasmid was cured, allowing the plasmid origin and resistance marker to be reused in subsequent experiments if needed. Detailed description of the RECKLEEN system’s workflow is provided in “Methods” section.

Following the optimization of the CRISPR-Cas9-mediated counterselection in the RECKLEEN system, we proceeded to implement a fast and convenient full editing protocol for the RECKLEEN system (Fig. 2). The workflow started by inserting annealed oligonucleotides into the RECKLEEN plasmid, generating a 20-nt spacer sequence in the *sgRNA* that precisely targets the protospacer sequence in the *Kp* genome. This plasmid was then introduced to *Kp* by electroporation. Next, the RECKLEEN plasmid-carrying cells were cultured in presence of IPTG to induce the *P*_*tac*_ promoter for expression of the lambda Red operon (*gam, exo*, and *beta*)^34^. These cells were then made electrocompetent, and donor DNA (dDNA) containing the homology arms for the targeted genomic sequence was introduced by electroporation. Subsequently, the cells were recovered in enriched SOC medium, and the recovered cells were cultured overnight on medium plates containing the inducer ATc (Fig. 2).

During these last two phases, some of the cells underwent homologous recombination with the dDNA, facilitated by the lambda Red components^9^. Concurrently, ATc induces the *P*_*tet*_ promoter to overexpress the *cas9* and *sgRNA* genes enabling the counterselection step by specifically targeting and cleaving the chromosome of the WT cells, which prevents further proliferation of these cells^21,22^. On another scenario, the DSB caused by the Cas9/sgRNA system in non-edited cells, still containing the lambda Red components and dDNA, may promote homologous recombination-based DNA repair^44^. In both scenarios, only cells that had replaced the target sequence against the modified sequence of the dDNA by recombination were able to evade the lethal Cas9/sgRNA-induced DSB, allowing them to grow on the ATc-containing medium plates. After confirming the accuracy of the edits, the genome-edited cells were cured from the RECKLEEN plasmid (Fig. 2). This allows the plasmid backbone and resistance marker to be reused in future experiments, making the system both efficient and recyclable.

### Evaluation of the RECKLEEN system as a genome editing tool

**Fig. 3:**
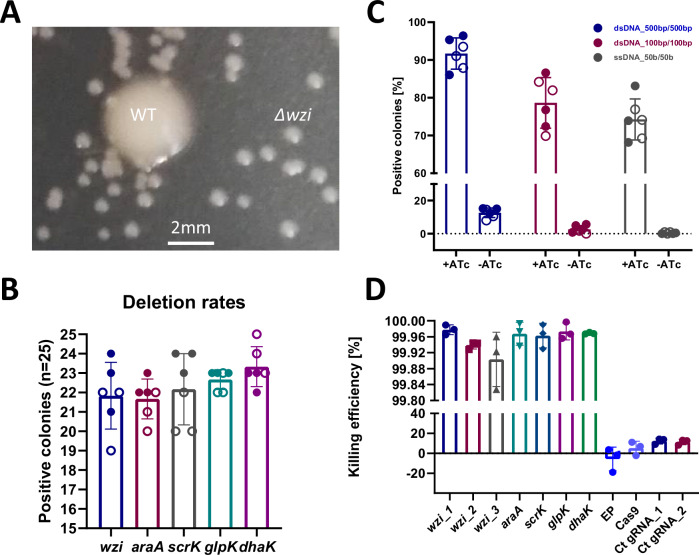
Characterization of the RECKLEEN 1-based method for gene deletions. **A** Phenotypic comparison between wild-type (WT) and Δ*wzi Kp*. Deletion of *wzi*, which encodes an outer membrane protein anchoring the capsular polysaccharide, led to observable changes in colony morphology and size. Morphological differences between WT and *wzi*-deleted colonies were used for prescreening prior to PCR validation. **B** Proof-of-concept evaluation of the RECKLEEN method for the deletion of various genes. *n* = 6 independent biological replicates (three per experiment, across two independent experiments). Individual replicates are shown as circles; filled and open symbols denote independent experiments. Error bars indicate standard deviation from the mean. **C** Effect of different donor DNA (dDNA) templates on the RECKLEEN editing efficiency. dDNA templates of varying lengths and types (dsDNA: 500 bp/500 bp, 100 bp/100 bp, and ssDNA: 50 bp) were used to assess the method’s efficiency for *wzi* deletion (by colony morphology), with (+ATc) and without (-ATc) *cas9* and *sgRNA* expression induced. *n* = 6 independent biological replicates (three per experiment, across two independent experiments). Individual replicates are shown as circles; filled and open symbols denote independent experiments. Error bars indicate standard deviation from the mean. **D** Killing efficiency for different sgRNAs targeting the sequence of various deletion targets in the *Kp* genome. EP denotes usage of the empty plasmid (Level 0* plasmid: contains pMB1 origin of replication, *aac3(IV)* apramycin resistance), Cas9 denotes usage of P_*tet*_ Cas9 plasmid (Level 1 plasmid: P_tet_ *cas9*), and Ct gRNA indicates usage of the RECKLEEN 1 plasmid with control *sgRNA*. The sequences of sgRNAs are detailed in Supplementary Table 3. The killing efficiency was calculated as follows: ${Killing\; efficiency}\left[ \% \right]=1-\frac{\frac{{CFU}}{{mL}}{in\; presence\; of\; inducer}\left({with\; counterselection}\right)}{\frac{{CFU}}{{mL}}{in\; absence\; of\; inducer}\left({without\; counterselection}\right)}* 100$ to measure the percentage of killed cells upon induction of the *cas9* and *sgRNA* expression by ATc. Data represents the mean of three biological replicates (*n* = 3), individual replicates are shown and the error bars represent the standard deviation from the mean.

To evaluate the efficacy of the RECKLEEN method, we selected *wzi* as an initial target for deletion. This gene encodes an outer membrane protein that anchors the capsular polysaccharide of *Kp*^37^. We used double-stranded dDNA template with 500 bp homology arms on each side of the target sequence (Supplementary Table 5). Following application of the editing workflow, we observed a distinct phenotypic divergence in colony morphology (size and mucoid phenotype) (Fig. 3A). Given the expected phenotypic change upon capsule loss, colonies displaying reduced size and a non-mucoid appearance were prioritized for screening. To test for loss of *wzi*, twenty-five colonies with a smaller and less mucoid appearance were selected and screened by colony PCR. One representative positive clone was further validated by Sanger sequencing (Supplementary Fig. 2B). The editing efficiency for the *wzi* deletion was 87.3 ± 6.3%, with individual values ranging from 76% to 96% (Fig. 3B). These results reflect the high efficiency of the RECKLEEN method for *wzi* deletion.

### Impact of various dDNA templates and sgRNA positions on the editing efficiency of the RECKLEEN system

Lambda Red recombination has been shown to be effective using numerous types and sizes of the dDNA templates^12,15,21^. Taking advantage of the change in colony morphology caused by a *wzi* deletion, we tested the editing efficiency of the RECKLEEN system using double-stranded dDNA with homology arms of 500 bp/500 bp and 100 bp/100 bp as well as single-stranded dDNA of 50 b/50 b flanking the deletion^29^ (Supplementary Tables 5 and 6). Editing efficiencies >70% were observed across the various types of tested dDNA (Fig. 3C). The highest efficiency of 91.7 ± 3.8% was achieved with the 500 bp/500 bp double-stranded dDNA. Notably, the use of ssDNA of 50 bases achieved an editing efficiency of 74.3 ± 5%. This demonstrates that synthetic single-stranded oligonucleotides can be used in the RECKLEEN method, eliminating the need for time-consuming preparation of dDNA templates.

In contrast, the absence of ATc-induced *cas9/sgRNA*-mediated counterselection resulted in a remarkable decrease in the editing efficiency across the various types of tested dDNA. The highest observed efficiency was 12.6 ± 2.6% using 500 bp homology arms and was negligibly low with shorter homology arms (Fig. 3C). This highlights the critical role of *cas9/sgRNA*-mediated counterselection in enhancing editing efficiency and ensuring successful genome modifications.

To assess whether the sgRNA position within a gene affects editing efficiency, we designed three distinct sgRNAs targeting the *wzi* coding sequence at nucleotide positions 359 (sgRNA1), 790 (sgRNA2), and 1311 (sgRNA3) (Supplementary Table 3). All constructs utilized the same RECKLEEN 1 backbone and identical dDNA (double-stranded dDNA with homology arms of 500 bp/500 bp) to delete *wzi* (Supplementary Table 5). Cas9-mediated killing efficiencies were uniformly high for all sgRNAs, with values of 99.978 ± 0.01% (sgRNA1), 99.944 ± 0.008% (sgRNA2), and 99.903 ± 0.056% (sgRNA3) (Fig. 3D). The editing efficiencies were 91.9 ± 3.3% and 93.26 ± 3.8% for sgRNA1 and sgRNA3, respectively (Supplementary Fig. 7B). The sites targeted by these sgRNAs for cleavage are proximal to the ends of the homology arms. In contrast, the editing efficiency of 78.64 ± 1.9% for sgRNA2 was considerably lower (Supplementary Fig. 7B). This sgRNA targeted a site near the center of the gene. Despite this variation, all three sgRNAs supported effective editing, underlining the robustness and versatility of the RECKLEEN platform. Nonetheless, these findings suggest that sgRNA positioning relative to the donor DNA homology arms plays a role in determining the overall editing efficiency, with sites proximal to homology regions providing enhanced recombination and/or selection efficacy. This observation aligns with prior findings in recombineering systems, where proximity of the DSB to the homology-directed repair (HDR) site enhances editing rates by facilitating more efficient recombination initiation^45,46^.

### Deletion of various genes in a proof-of-principle application of the RECLKEEN system

In order to test the performance of the RECKLEEN system for generating gene deletions more broadly, we selected four genes as deletion targets that are located at different positions across the *Kp* chromosome. The selected genes are involved in the catabolic pathway of several carbon sources in *Kp*, including arabinose (*araA*)^47^, sucrose (*scrK*)^48^, and glycerol (*dhak* and *glpK*)^29,49,50^. We applied the complete RECKLEEN method workflow using phosphorothioate-modified single-stranded oligonucleotides targeting the lagging strand and representing 50 bases homology arms on each side of the sequence to be deleted (Supplementary Table 6). Initially, the recovered colonies were screened for growth on minimal medium containing a single carbon source, glucose as control, and either arabinose, sucrose or glycerol for the experiment^50^, which allowed to assess the percentage of mutant colonies by growth phenotype (Supplementary Figs. 3A–5A). The desired gene deletions were further confirmed by Sanger sequencing for one randomly selected mutant per experiment (Supplementary Figs. 3B–5B).

Killing efficiencies of 99.967 ± 0.024%, 99.963 ± 0.025%, 99.973 ± 0.017%, and 99.969 ± 0.002% were achieved for *araA, scrK, glpK*, and *dhak*, respectively (Fig. 3D). Among the randomly selected colonies, a deletion efficiency of 89.8 ± 1.7% was achieved for the target genes, as assessed by the growth phenotype, with the highest editing efficiency for the deletion of *dhak* (93.3 ± 3.7%) and the lowest for *araA* (86.6 ± 3.7%) (Fig. 3B). These results demonstrate the high efficiency of the RECKLEEN method for gene deletion across different loci in the *Kp* genome.

### Optimization of the RECKLEEN method for generating point mutations and DNA integrations

To cleave DNA at its target site the Cas9/tracrRNA:crRNA complex requires both the recognition sequence where the DSB is introduced and 5′-NGG-3′ as a PAM sequence^18,23,26,27^. This limits the design space for the spacer guide sequence^28^. In the case of deletions, the removal of the entire coding sequence provides numerous options for design of this sequence, enabling straightforward differentiation between edited and WT cells^22^. However, differentiating WT from edited sequence in case of point mutations and DNA integrations is hampered by the need of a nearby 5′-NGG-3′ PAM sequence^27^.

**Fig. 4:**
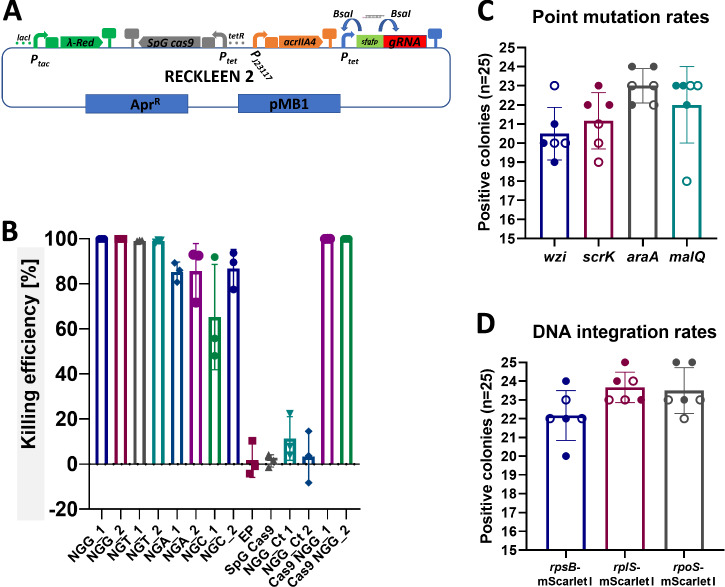
RECKLEEN 2-based platform for generating point mutations and DNA integrations using SpG Cas9 variant. **A** Design of the RECKLEEN 2 plasmid. This plasmid retains the same overall design as RECKLEEN 1 but utilizes the SpG Cas9 variant instead of Cas9, incorporating six specific amino acid mutations in the *cas9* coding sequence (D1135L, S1136W, G1218K, E1219Q, R1335Q, and T1337R). **B** Killing efficiency of various plasmids assembled using the RECKLEEN 2 platform with different sgRNAs targeting all possible PAM sequences (two of each possible PAM sequences), EP denotes usage of the empty plasmid (Level 0* plasmid: contains pMB1 origin of replication, *aac3(IV)* apramycin resistance), SpG Cas9 denotes usage of P_*tet*_ SpG *cas9* plasmid (Level 1 plasmid: P_*tet*_ SpG *cas9*), and Ct gRNA indicates usage of the RECKLEEN 2 plasmid with control sgRNA. The sequences of sgRNAs are detailed in Supplementary Table 3. Data represent the mean of three biological replicates (*n* = 3), individual replicates are shown with error bars representing the standard deviation from the mean. Evaluation of the RECKLEEN 2 platform for point mutations (**C**) and DNA integrations (**D**) across various targets in *Kp* genome. *n* = 6 independent biological replicates (three per experiment, across two independent experiments). Individual replicates are shown as circles; filled and open symbols denote independent experiments. Error bars indicate standard deviation from the mean.

To address this challenge, we harnessed an engineered variant of Cas9, SpG Cas9^28^. This variant has previously shown its functionality with a broader range of PAM sequences, requiring only an NGN sequence, where a single G nucleotide at the mutation site is sufficient for sgRNA design^27,28^. Based on the same design as the RECKLEEN 1 plasmid, we constructed the RECKLEEN 2 plasmid (Fig. 4A), incorporating an SpG Cas9 encoding gene instead of *cas9*. This was achieved, as previously described^27,28^, by introducing mutations in the *cas9* coding sequence leading to six specific amino acid exchanges (D1135L, S1136W, G1218K, E1219Q, R1335Q, and T1337R).

We used RECKLEEN 2 to construct plasmids encoding sgRNAs targeting various sequences of the *wzi* coding sequence with all possible PAM sequences (NGA, NGT, NGC, NGG) (Supplementary Table 3). We demonstrated the functionality of the SpG Cas9 variant with the different PAM sequences in *Kp*. It achieved killing efficiency of 99.904 ± 0.019% and 99.052 ± 0.360% with sgRNAs using NGG and NGT as PAM sequences, respectively. Lower killing efficiencies were observed with sgRNAs using NGA (85.531 ± 7.457%) and NGC (76.076 ± 17.974%) as PAM sequences (Fig. 4B). These results show that the SpG Cas9 variant significantly expands the pool of sgRNAs that can be designed for Cas9/sgRNA-mediated DNA cleavage for counterselection, using not only NGG, but also NGT, NGA, and NGC PAM sequences (Fig. 4B).

#### Point mutations are possible using RECKLEEN 2

To assess the capacity of RECKLEEN 2 for generating point mutations, we selected four genes (*wzi*, *scrK*, *araA*, and *malQ*) and introduced single-nucleotide substitutions that created early stop codons. The specific codons—C66 in *wzi* (Tyr22), T222 in *scrK* (Tyr74), C57 in *araA* (Tyr19), and T57 in *malQ* (Tyr19)—were strategically chosen based on their potential to be converted into a TAA nonsense codon through a single base change. These premature termination codons were placed early in the coding sequences to ensure functional inactivation of the respective gene products, thereby facilitating phenotypic screening of mutant clones. For instance, *wzi* mutants exhibit altered capsule-associated colony morphology, whereas *scrK*, *araA*, and *malQ* mutants display impaired growth on selective carbon sources. Importantly, these sites were also selected to demonstrate the extended targeting capabilities of the SpG Cas9 variant, as none of them had an adjacent canonical 5′-NGG-3′ PAM. Instead, alternative PAM sequences were employed: GGT for *wzi* and *scrK*, GGC for *araA*, and GGG for *malQ* (Supplementary Table 3). The use of SpG Cas9 allowed robust targeting at these non-NGG sites, resulting in high killing efficiencies of 99.965 ± 0.006% (*wzi*), 99.995 ± 0.001% (*scrK*), 96.651 ± 0.205% (*araA*), and 99.991 ± 0.01% (*malQ*) (Supplementary Fig. 7).

By applying the complete editing protocol in three biological replicates and two independent experiments, we successfully introduced the desired point mutation in *scrK* (Supplementary Fig. 9) and *araA* (Supplementary Fig. 10). The editing efficiency for *scrK* was 84 ± 5.37% and ranged from 76% to 92%, while for *araA* it was 92 ± 3.3% and ranged from 88% to 96% (Fig. 4C). The presence of the desired mutations was confirmed via Sanger sequencing of a PCR fragment derived from at least one positive clone per experiment (Supplementary Figs. 8B and 9B). However, we were unable to introduce the point mutations in *wzi* and *malQ*. Only a few clones were recovered, all of which contained the WT sequence. A potential factor influencing the stability of mismatches introduced to the genomic DNA is the mismatch repair (MMR) mechanism that maintains genomic stability by correcting base-base mismatches generated during DNA replication and recombination^51–53^. To overcome this issue, we used a strategy in which the dDNA introduced not only the desired mutation but also another silent mutation, creating a mismatch that could inhibit the repair mechanism, as previously demonstrated in several studies^29,52,54^. Specifically, we selected G57 in *wzi* and G66 in *malQ* to be edited into cytosine (C), which would generate a CC mismatch producing a silent mutation of an alanine codon alongside the previously designed early stop codon (Supplementary Table 6). This approach resulted in clones carrying the desired point mutations with success rates of up to 92% for both targets. The editing efficiencies were 82 ± 5.03% and 88 ± 7.3% for *wzi* and *malQ*, respectively (Fig. 4C and Supplementary Figs. 8 and 11).

#### DNA integrations are possible using RECKLEEN 2

Next, we applied the RECKLEEN 2 system for DNA integrations, aiming to fuse the mScarlet-I protein to various targets whose expression levels are known to change during different growth stages in various organisms, for example, *E. coli* and *V. natriegenes*^29,55–57^. We selected RpsB and RplS, two ribosomal proteins with levels known to increase during the exponential phase of bacterial growth^56^. Additionally, we targeted the stress sigma factor RpoS, a key regulator of the general stress response, which is primarily produced during the stationary phase^55,57^. This allowed us to monitor fusion protein levels using mScarlet-I as a reporter signal.

To achieve this, we designed a fusion of the *mScarlet-I* coding sequence^34^ to the 3′ end of each gene’s coding sequence, directly before the stop codon. We prepared dDNA templates containing the mScarlet-I coding sequence flanked by 500 bp homology arms for each gene (Supplementary Tables 4 and 5). Using SpG Cas9, we performed the counterselection against the WT cells with *sgRNAs* using as PAM sequences CGA for *rpsB*, CGC for *rplS*, and TGT for *rpoS* (Supplementary Table 3). The killing efficiencies were 99.553 ± 0.018%, 99.627 ± 0.033%, and 99.979 ± 0.019% for *rpsB*, *rplS*, and *rpoS*, respectively (Supplementary Fig. 7). By applying the complete editing protocol in three biological replicates and two independent experiments, we successfully recovered numerous colonies of clones with the integration mutations as designed (Supplementary Figs. 13–15). The integration efficiencies were 88.6 ± 4.9% for *rpsB*, 94.67 ± 3% for *rplS*, and 94 ± 4.5% for *rpoS* (Fig. 4D).

The integration of mScarlet-I did not affect the growth of *Kp* (Supplementary Fig. 12A), consistent with previous studies in *E. coli*, *V. natriegens*, and other species^29,55,56^. We monitored the mScarlet-I reporter signal in both WT and the strains with RpsB-mScarlet-I, RplS-mScarlet-I, and RpoS-mScarlet-I fusions throughout bacterial growth. The strains with the mScarlet-I fusions to the ribosomal proteins showed an increasing signal after 2 h until 8 h of growth, after which the signal reached a steady state (Supplementary Fig. 12B, E–F). In contrast, the strain with the RplS-mScarlet-I fusion showed a significant increase in signal during the stationary phase after almost 8 h of bacterial growth (Supplementary Figs. 12B, D).

### Optimization of the RECKLEEN method for simultaneous multi-editing

**Fig. 5:**
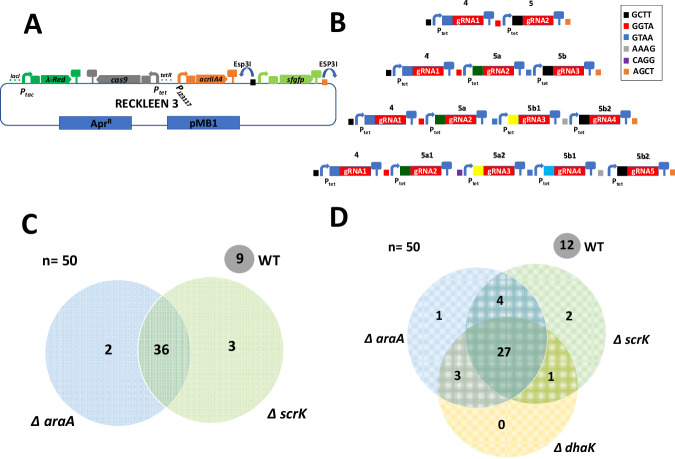
RECKLEEN 3 platform for simultaneous multi-target genome editing. **A** Design of the RECKLEEN 3 plasmid containing the main transcription units of the lambda Red components, Cas9, ACRIIA4, as described for RECKLEEN 1 and a *sfgfp* dropout part where two to five different *sgRNA* cassettes can be introduced using eight sgRNA helper plasmids (RECKLEEN 3plus_helper sgRNA plasmids). Customizing of the RECKLEEN 3 plasmid for simultaneous editing of different targets is done by two Golden Gate Cloning steps as detailed in the method section. **B** A scheme of the sgRNA helper plasmids showing the different plasmids which can be combined to assemble plasmids with *sgRNA* modules for targeting 2, 3, 4, and 5 different targets simultaneously. Fusion sites (5′→3′ direction) are introduced based on predicted assembly efficiency^36^ and are indicated in the inlay box. Evaluation of the RECKLEEN 3 platform for simultaneous deletion of two genes (**C**) and three genes (**D**) using a single editing plasmid.

Taking advantage of the high editing efficiency of the RECKLEEN system, we developed the RECKLEEN 3 platform, designed for simultaneous gene editing. RECKLEEN 3 contains the main transcription units of the RECKLEEN 1 system, with the coding sequences for the lambda Red operon (*gam, exo*, *beta*), *cas9*, and *acrIIA4*, along with a *sfgfp* dropout module (Fig. 5A). This setup allows for the introduction of two to five different *sgRNA* genes via eight sgRNA helper plasmids (Fig. 5B). The multi-target plasmid can be assembled through two straightforward Golden Gate cloning steps using BsaI and Esp3I, as illustrated in Fig. 5A, B and detailed in the methods section.

We constructed two multi-target plasmids to test the system’s capability for simultaneous deletion of two genes (*araA* and *scrK*) and three genes (*araA*, *scrK*, and *dhaK*) (Supplementary Tables 1 and 3). The killing efficiencies were 99.997 ± 0.001% for the construct designed for *araA* & *scrK* deletion, and 99.998 ± 0.001% for that designed for *araA* & *scrK* & *dhaK* deletion (Supplementary Fig. 7). Upon completion of the full editing protocol, we tested fifty randomly selected colonies for the designed mutations by growth phenotype on minimal medium with respective carbon sources. We achieved the desired gene deletions in 72% and 54% of the randomly selected cells for the two-gene and three-gene deletions, respectively (Fig. 5C, D). In the two-gene deletion experiment, approximately 4% of the selected clones exhibited deletion of only *araA* but not *scrK*, while 6% showed deletion of only *scrK* but not *araA* (Figs. 5C and S15). In the three-gene deletion experiment, around 6% of the selected clones displayed deletion of just one gene (2% for *araA* and 4% for *scrK*). Additionally, 16% of the selected clones exhibited deletion of two genes but not the third, with 8%, 6%, and 2% showing deletions of *araA* & *scrK*, *araA* & *dhaK*, and *scrK* & *dhaK*, respectively (Figs. 5D and S16).

### Curing of RECKLEEN plasmids after genome editing

Finally, we established a procedure for curing the genome-edited cells from the RECKLEEN editing plasmid. Curing the plasmid not only allows reusing key components, such as the plasmid origin and resistance marker, in future experiments if needed. It also avoids unintended influence of the plasmid on phenotypes and molecular processes to be analyzed in the edited cells. We pursued curing of the plasmid by eliminating the selection pressure provided by the antibiotic^58^. Colonies obtained after genome editing using the RECKLEEN method were inoculated into antibiotic-free LB and incubated overnight at 37 °C. A 10⁻⁶ or 10⁻⁷ dilution of the overnight culture was then plated on agar without apramycin to isolate single colonies. The following day, individual colonies were streaked onto plates with and without apramycin. Cells with cured plasmids failed to grow on apramycin-containing plates but grew normally on apramycin-free agar, allowing for their recovery and storage. Using this approach, we achieved 98 ± 1.3% efficiency in curing edited cells from the plasmid.

**Fig. 6:**
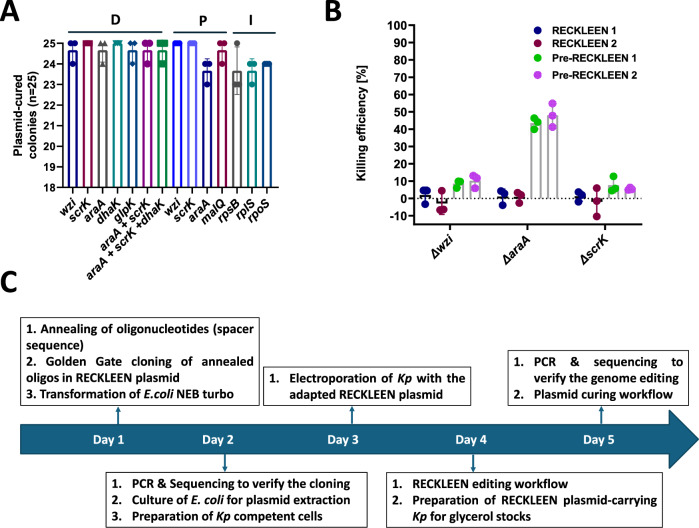
Plasmid curing efficiency, on-target specificity, and streamlined workflow of the RECKLEEN system. **A** Plasmid curing efficiency. Evaluation of RECKLEEN plasmid curing of the cells with the different targets edited. “D” denotes deletions, “P” point mutations, and “I” DNA integrations. Data represent the mean of three biological replicates (*n* = 3), individual replicates are shown, and the error bars represent the standard deviation from the mean. **B** Validation of on-target specificity and anti-CRISPR (AcrIIA4) suppression of background killing post-ATc induction in RECKLEEN-edited strains. Killing efficiencies under inducing conditions were determined after re-introducing RECKLEEN 1 (Cas9) or RECKLEEN 2 (SpG Cas9) plasmids into gene-edited *Kp* strains (Δ*wzi*, Δ*scrK*, and Δ*araA*). Parallel assays using equivalent constructs lacking the AcrIIA4 anti-CRISPR unit (Pre-RECKLEEN 1 and 2). Data represents the mean of three biological replicates (*n* = 3); individual replicates are shown with error bars representing the standard deviation from the mean. **C** Timeframe of the RECKLEEN workflow. Under ideal conditions, five working days are sufficient for the complete editing protocol. Verification of plasmid loss after plasmid curing can be performed in the next steps. All individual steps are described in detail in the Methods section.

Curing efficiency ranged from 94.7 ± 1.8% for strains with the *araA* point mutation and those with *mScarlet-I* integration at the 3′ end of *rplS*, to 100% for strains with deletions of *scrK* and *dhak*, as well as point mutations in *wzi* and *scrK* (Fig. 6A). This demonstrates that the cells tend to efficiently lose the plasmid once the selection pressure for the antibiotic resistance cassette is removed. The possibility to easily cure cells of the plasmid makes the RECKLEEN system even more user-friendly for genome editing and subsequent experimental reuse.

### The RECKLEEN system can edit the genome of various MDR *Kp* strains

Next, we tested the applicability of the RECKLEEN system as a genome editing tool in various MDR *Kp* strains. Under ideal conditions, five working days are sufficient for the complete editing protocol, starting from customizing the RECKLEEN plasmid with the specific 20-nt guide spacer sequence to the genome-edited *Kp* cells (Fig. 6C). To this end, we aimed to delete the coding sequences of *wzi* from the chromosome of the MDR *Kp* strains ATCC BAA-1705 and ATCC 700603. We applied the workflow of the RECKLEEN editing protocol as summarized in Figs. 2 and 6C with dDNA sequences detailed in Supplementary Table 6. The observed killing efficiencies were 99.929 ± 0.037%, and 99.993 ± 0.002% for for *Kp* ATCC BAA-1705 and *Kp* ATCC 700603, respectively (Supplementary Fig. 7), which is comparable to the killing efficiency of 99.938 ± 0.008% obtained when this gene was deleted from the *Kp* ATCC 700721 genome (Fig. 3D). Both, in *Kp* ATCC BAA-1705 and ATCC 700603, out of ten selected colonies, nine colonies showed the targeted deletion of *wzi* (Supplementary Fig. 18). The desired mutation was further confirmed by Sanger sequencing of a PCR product derived from one of the positive clones (Supplementary Fig. 18). These results suggest that the RECKLEEN system can be used to edit the genome of various MDR *Kp* strains, paving the way for its application to a broader range of clinically relevant pathogens.

### RECKLEEN-mediated genome editing is highly specific

Our pilot experiments demonstrated that the RECKLEEN platform allows highly efficient, targeted genome editing in *Kp*. Killing efficiencies of almost 100% were consistently observed across different target loci, ranging from 99.938 ± 0.007% to 99.998 ± 0.001% (Figs. 3D, 4B and Supplementary Fig. 7). Since the use of the PAM-flexible SpG Cas9 variant can lead to more off-target effects upon gRNA mismatches^59^, we tested the specificity of the Cas9- (RECKLEEN1) and the SpG Cas9 (RECKLEEN2)-based systems in *Kp*. To quantify potential off-target toxicity or background killing, we tested two sets of control plasmids for their killing efficiencies upon ATc induction in *Kp*. In the assays of the Cas9-based system, we obtained killing efficiencies of −5.69 ± 9.69% for the empty RECKLEEN plasmid (no Cas9 and no gRNA), 5.25 ± 5.61% for the plasmid encoding Cas9 alone but no gRNA, and 12.3 ± 2.4% and 11.3 ± 2.2% for two plasmids encoding Cas9 and each a different control non-targeting sgRNAs (Supplementary Table 3 and Fig. 3D). Similarly, in the assays of the SpG Cas9-based system, we observed killing efficiencies of 1.7 ± 6.2% for the empty RECKLEEN plasmid, 1.4 ± 2.2% for the plasmid encoding SpG Cas9 alone, and 8.8 ± 7.9% and 3.3 ± 9.3% for the plasmids encoding SpG Cas9 and the control non-targeting sgRNAs (Fig. 4B). Thus, in all cases, background killing was negligible compared to that observed when expressing the target sgRNA genes. This indicates that the specific sgRNA on-target effect was responsible for the majority of the cells killed.

To analyze possible off-target effects of the system with sgRNAs that had been used for genome editing, the RECKLEEN 1 (Cas9) and RECKLEEN2 (SpG Cas9) plasmids expressing sgRNAs that target the deleted regions of previously edited strains (Δ*wzi*, Δ*scrK*, and Δ*araA*) were reintroduced to the respective deletion mutants. In all cases, the strains exhibited significant resistance to Cas9/SpG Cas9-mediated killing post-ATc induction, consistent with the loss of the targeted DNA sequences required for DNA cleavage. The killing efficiencies were reduced from 99.98 ± 0.01% (*wzi*), 99.97 ± 0.02% (*araA*), and 99.96 ± 0.03% (*scrK*) (Fig. 3D) to 1.93 ± 3.7% (Δ*wzi*), 1.05 ± 3.6% (Δ*araA*), and 1.23 ± 2.4% (Δ*scrK*) (Fig. 6B) in case of RECKLEEN 1. Similarly, very low RECKLEEN 2 plasmid-mediated killing efficiencies were observed in the deletion mutants (−2.9 ± 5.2% for Δ*wzi*, 0.9 ± 2.5% for Δ*araA*, and −1.9 ± 6.7% for Δ*scrK*) (Fig. 6B). These findings further confirm a high specificity for the on-target sequence. We also compared these results with the killing efficiencies of equivalent constructs lacking the anti-CRISPR AcrIIA4 transcription unit (Pre-RECKLEEN 1 and Pre-RECKLEEN 2). AcrIIA4 inhibits Cas9 post-cleavage, enabling precise temporal control over nuclease activity. Interestingly, elevated killing post-ATc induction was observed with the Pre-RECKLEEN constructs, particularly in the Δ*araA* strain. The calculated killing efficiencies in case of the Δ*araA* strain were 43.45 ± 2.6% and 47.95 ± 5.56% for Pre-RECKLEEN 1 and 2, respectively. This unintended activity was nearly abolished to 1.05 ± 3.6% and 0.89 ± 2.5% for RECKLEEN 1 and 2, respectively, both including *acrIIA4* (Fig. 6B). This highlights the functional importance of AcrIIA4 as a built-in safety switch in our system that buffers background production of Cas9 under non-inducing conditions and mitigates off-target Cas9 activity while preserving high on-target efficiency under non-inducing and inducing conditions.

Furthermore, we complemented the Δ*scrK*, Δ*araA*, and premature stop codon mutants (*scrK*(T222A) and *araA*(C57A)) with plasmids expressing the respective WT genes (Supplementary Tables 1 and 2). Growth on sole carbon sources (sucrose or arabinose for *scrK* and *araA* mutants, respectively) was restored only in the complemented strains. This confirms that the observed phenotypes result from the intended targeted gene disruptions rather than from off-target effects (Supplementary Figs. 19 and 20).

To evaluate potential off-target effects more comprehensively, we re-sequenced the genome of the WT strain and sequenced the genome of the Δ*wzi* strain that had been generated using the RECKLEEN system. Analysis of the sequencing data revealed no off-target single nucleotide variants (SNVs), indels, or structural variations associated with Cas9 activity (Supplementary Table 7), supporting the precision of the system.

Taken together, our data indicate that RECKLEEN-mediated genome editing is highly specific. These findings are consistent with prior reports of high specificity and low off-target activity of Cas9/spG Cas9 under appropriate control^27–29,42,43,60^. Our NT-CRISPR system including ACRII A4 and optimized for tightly controlled expression in *Vibrio natriegens* has demonstrated high targeting specificity^29^. In their landmark study, Walton et al. engineered *Streptococcus pyogenes* Cas9 variants and rigorously quantified these variants for genome-wide specificity using GUIDE-seq, a high-sensitivity method for detecting off-target cleavage^27^. They observed off-target effects for engineered variants like SpG (NGN PAMs) and SpRY (NRN and to a lesser extent NYN PAMs) at levels similar to those of the wild-type enzyme. Liang et al. reported that SpG Cas9 exhibited minimal off-target effects in zebrafish, with off-target mutation rates of 0 to ≤0.5% across multiple gRNAs, as determined by CRISPResso2 through high-throughput next-generation sequencing^28^.

### Long-term stability and fitness of edited strains

To assess the genetic stability of RECKLEEN-generated mutations over time, we subjected three representative RECKLEEN plasmid-cured strains to serial passaging for ten consecutive generations under conditions non-selective for the respective mutation: (i) a gene deletion mutant (*Δwzi*), (ii) a point mutation strain (*wzi C66A*), and (iii) a chromosomal insertion strain (*rpoS-mScarletI*). Genomic DNA was extracted at passages five and ten, and the corresponding edited loci were PCR-amplified and subjected to Sanger sequencing. The intended mutations remained stable in all studied populations, with no evidence of reversion or mutant phenotype-suppressing secondary mutations (Supplementary Figs. 21–23).

To evaluate potential fitness costs associated with these edits, we monitored growth kinetics of the WT and the edited strains at passages 1, 5, and 10. Compared to the WT strain, the growth kinetics of all mutants remained unchanged, showing no significant difference in doubling times or overall growth profiles (Supplementary Fig. 24). These findings confirm that the RECKLEEN-edited genotypes tested here are stably maintained and do not compromise cell viability or fitness under standard laboratory conditions.

In summary, we engineered the RECKLEEN system as a single plasmid platform for fast, precise, efficient, markerless, and scarless genome editing in *Kp*. The RECKLEEN system combines the potential of lambda Red recombineering and the powerful Cas9/sgRNA system as a counterselection approach. Compared to previously reported traditional as well as lambda Red/CRISPR-Cas9-based tools, we are convinced that the RECKLEEN system offers significant advantages for precise genome engineering in various MDR *Kp* strains. Many traditional methods^12,13,21,30^ and a previously reported lambda Red/CRISPR-Cas9-based system^16^ for genetic manipulation of *Kp* use multiple antibiotic resistance markers, whereas the single-plasmid RECKLEEN system requires only a single antibiotic resistance marker. This is a significant advantage for manipulating the genome of MDR strains, where resistance to multiple antibiotics often limits the selection options. A single-plasmid design also simplifies the delivery of the system, reduces the burden on the host, and facilitates curing of the edited cells from the system.

The RECKLEEN system is considerably improved in several aspects over the lambda Red/CRISPR-Cas9 tools previously developed for *Kp*^8,15,31^. These earlier tools provided a valuable foundation for scarless editing via two-plasmid systems and transient λ-Red/Cas9 expression. Yet, RECKLEEN introduces several key innovations that enhance flexibility, efficiency, and specificity. First, RECKLEEN consolidates all essential editing components—λ-Red recombinase, Cas9 (or SpG Cas9), sgRNA, and an anti-CRISPR ACRIIA4 safety module—into a single plasmid. This design eliminates the need for multi-step transformations, helper plasmids, multi-curing steps or chromosomal integrations. Second, RECKLEEN expands the editing capability by employing the SpG Cas9 variant, which recognizes a broad NGN PAM range. This enables access to AT-rich regions of the genome that are often not targetable with NGG-restricted Cas9 used in earlier systems. This increased PAM flexibility was critical in enabling efficient editing at sites where an NGG PAM sequence is not available (e.g., in case of point mutations *araA (C57A)* and *scrK (T222A)*, and DNA integrations *rpoS-mScarlet-*I, *rplS-mScarlet-*I, and *rpsB-mScarlet-*I generated in this study). Third, RECKLEEN incorporates AcrIIA4, an anti-CRISPR protein that suppresses Cas9/SpG Cas9 activity prior to induction, minimizing off-target cleavage and leaky toxicity. This safety switch is a major advantage over previous constructs, as demonstrated by the significantly reduced background killing in RECKLEEN versus Pre-RECKLEEN controls lacking AcrIIA4 (Figs. 4B and 6B). Fourth, RECKLEEN also supports simultaneous multiplexed editing through modular gRNA assembly, expanding its utility for synthetic biology applications.

Within a protocol of 5 days (7 days including final plasmid curing), the RECKLEEN system could introduce the desired mutation to *Kp*, which is significantly faster than time-consuming traditional methods^12,13,21,30^ and the 14-days protocol of the CRISPR-Cas9 system previously reported by McConville et al.^8^. This was achieved by a streamlined process and a modular design concept of the RECKLEEN system based on the widely used MoClo standard, which enables efficient, flexible and scalable assembly of plasmids for genome editing. This modularity allows straightforward customization of the base plasmid for the genetic target(s) in just two days and significantly reduces the resources required. It also facilitates replacing the apramycin resistance gene used to select for plasmid presence against another selection marker gene if required.

These advantages are not limited to the *Kp* MGH78578 strain used to establish the RECKLEEN system, as we have successfully applied our tool to genome editing in several MDR *Kp* strains, demonstrating its potential for broader applications in clinically relevant pathogens. Thus, RECKLEEN provides a robust, modular, highly specific, and broadly applicable editing tool. We believe that the RECKLEEN system will enable studies toward a deeper understanding of *Kp* pathology and thereby contribute to the efforts to combat these critical public health threats.

## Materials and methods

### Bacterial strains and culture conditions

The *Kp* strains used in this study, including *Kp* ATCC 700721 (MGH78578), *Kp* ATCC 700603, and *Kp* ATCC BAA-1705, were obtained from the American Type Culture Collection (ATCC, Rockville, MD, USA). In addition, *Escherichia coli* NEB Turbo and *E. coli* DH5α were used for cloning purposes. All strains were routinely cultured in lysogeny broth (LB) medium, consisting of 5 g/L yeast extract, 10 g/L tryptone, and 10 g/L NaCl (pH 7.2–7.4). Solid medium contained 15 g/L agar. For screening of *Kp* carbon utilization mutants, M9 agar plates were prepared (for 100 mL) by autoclaving H_2_0 (35.7 mL) together with 1.5 g agar. Subsequently all remaining components were prewarmed to 60 °C and added to the autoclaved components as follows: 50 mL 2 x M9 salts (Na_2_HOP_4_ (17 g/L), KH_2_PO_4_ (6 g/L), NaCl (1 g/L), NH_4_Cl (2 g/L), sterile filtered), 10 mL NaCl (20%), 4 mL carbon source (10%), 200 µL MgSO_4_ (1 M), 100 µL (CaCl_2_). Where required, antibiotics were added at the following concentrations: apramycin (50 µg/mL), kanamycin (50 µg/mL), and chloramphenicol (30 µg/mL). For long-term storage, bacterial cultures were grown overnight at 37 °C, and 700 µL of the culture was mixed with 700 µL of 50% glycerol before freezing at −80 °C.

### Construction of RECKLEEN plasmids

The RECKLEEN toolbox consists of eleven plasmids that were used to generate the deletions, point mutations, and DNA integrations: three RECKLEEN plasmids and eight sgRNA helper plasmids (RECKLEEN_plus_helper plasmids), designed for the construction of multi-target plasmids (Supplementary Table 1). Plasmid maps are provided in the Supplementary Data 1. All plasmids were assembled within the framework of the Marburg Collection, a recently developed Golden Gate cloning-based toolkit^34^.

Briefly, a Level 0* plasmid containing a pMB1 origin of replication and an apramycin resistance cassette was constructed from individual parts in the Marburg Collection plasmid toolbox. The Level 1 plasmids carry either *P*_*tac*_ promoter-driven lambda Red operon (*gam, exo*, and *beta*), *P*_*tet*_ promoter-driven *cas9* or *SpG cas9*, *P*_*tet*_ promoter-driven s*gRNA*, and constitutive promoters (J23117, J23105, or J23114)- driven *acrIIA4* (Supplementary Table 1). These Level 0* and Level 1 plasmids were used to assemble the final Level 2 RECKLEEN plasmids. Plasmid assembly was carried out in *E. coli* NEB Turbo, following previously established protocols^34,61^.

The sgRNA helper plasmids were similarly constructed using the Marburg Collection framework. These plasmids feature a ColE origin of replication, a kanamycin resistance cassette, and a *sfgfp* dropout module positioned upstream of the *sgRNA* scaffold under the control of the *P*_*tet*_ promoter. They are designed to enable the simultaneous targeting of up to five genes. The construction strategy relies on the relative positions within the Level 2 RECKLEEN 3 plasmid assembly^34,61^ (Fig. 5B).

### Preparation of competent cells and electroporation

Electrocompetent cells of *Kp* were prepared following previously established protocols^8^. Briefly, a 1 mL overnight culture derived from a fresh single colony was diluted into 120 mL of LB broth and incubated at 30 °C. The culture was grown to an optical density at 600 nm (OD_600_) of ~0.4 to 0.6, after which the cells were immediately chilled on ice for 20 min. The cells were then harvested by centrifugation at 7200 × *g* for 5 min, the supernatant was discarded, and the pellet was resuspended in 15 mL of sterile ice-cold 10% glycerol. The centrifugation and resuspension steps were repeated three times. Finally, the cells were resuspended in 1 mL of ice-cold 10% glycerol. Aliquots of 50 μL were frozen in liquid nitrogen and stored at −80 °C.

For electroporation, 100 μL of thawed electrocompetent cells were mixed with no more than 5 μL of plasmid DNA (at least 500 ng). This mixture was transferred to a 2-mm electroporation cuvette (Bio-Rad) and subjected to electroporation at 2.5 kV, 200 Ω, and 25 μF. Following the pulse, 1 mL of antibiotic-free SOC medium (0.5% yeast extract, 2% tryptone, 10 mM NaCl, 2.5 mM KCl, 10 mM MgCl₂, 10 mM MgSO₄, and 20 mM glucose) was added to the cuvette, and the cells were incubated at 30 °C for 3 h. The cells were subsequently plated onto LB agar containing the appropriate antibiotics and incubated overnight at 30 °C.

### Detailed description of the RECKLEEN system workflow

#### Design of *sgRNAs* for the different targets

*sgRNAs* were designed primarily using the CRISPOR.org web tool for genome editing experiments with the CRISPR–Cas9 system^62^. CRISPOR identifies potential sgRNA sequences in an input target sequence and ranks them based on multiple scoring metrics, including predicted off-target effects within the genome and anticipated on-target activity. The design process followed the algorithm outlined by Concordet & Haeussler^62^. From the list of predicted sgRNAs, those with the highest predicted activity scores were selected. We chose a 20 bp-spacer sequence before a PAM site (5′-NGG-3′ or 5′-NGN-3′ in case of *cas9* or *SpG cas9*, respectively) in the target locus. The two oligos were designed in the following forms:

Spacer-F: 5′-**GTCC**NNNNNNNNNNNNNNNNNNNN-3′

Spacer-R: 3′-NNNNNNNNNNNNNNNNNNNN**CAAA**-5′

The oligonucleotides were annealed by mixing 1.5 µL of each oligonucleotide (100 µM) with 5 µL of T4-DNA ligase buffer (10X) (Thermo Scientific) in a total reaction volume of 50 µL. The mixture was incubated at 95 °C for 15 min, after which the heating block was turned off, allowing the samples to cool gradually to room temperature over approximately 1 h. All oligonucleotide sequences used in *sgRNA* assembly are provided in Supplementary Table 3.

#### Customizing the RECKLEEN plasmids for specific targets (spacer cloning in RECKLEEN plasmids)

Adaptation of the RECKLEEN plasmids for editing of different target sequences was achieved by replacing a *sf*gfp dropout fragment with a designed *sgRNA* spacer sequence through the annealed complementary oligonucleotides as described earlier. Unless otherwise specified, RECKLEEN 1 was used for plasmids carrying single *sgRNAs* with *cas9*, and RECKLEEN 2 was used when *SpG cas9* was required.

For spacer cloning into RECKLEEN plasmids, the reaction was set up with ~150 ng of the respective plasmid, 5 µL of annealed oligonucleotides, 0.5 µL of T4-DNA ligase (5 Weiss U/µL, Thermo Scientific), 1 µL of T4-DNA ligase buffer (10X), and BsaI (10 U/µL) in a final volume of 10 µL. The thermocycler was programmed for 30 cycles at 37 °C for 2 min and 16 °C for 5 min, followed by a final digestion step at 37 °C for 30 min, and enzyme inactivation at 80 °C for 10 min^63^. Transformation of *E. coli* NEB Turbo was performed using 5 µL of the cloning reactions via heat shock transformation. Three colonies from each transformation plate were used as biological replicates in each experiment. The sequence was confirmed via Sanger sequencing and the plasmids were finally introduced into *Kp* electrocompetent cells by electroporation as described earlier.

For RECKLEEN plasmids containing multiple *sgRNA* genes, each *sgRNA* expression cassette was first constructed individually on RECKLEEN 3plus_helper sgRNA plasmids (carrying a kanamycin resistance marker; Supplementary Table 1 and plasmid maps in Supplementary Data 1). Depending on the number of target loci, different combinations of these plasmids can be utilized, as illustrated in Fig. 5B. Spacer insertion into these sgRNA helper plasmids followed the procedure outlined above. Subsequently, the *sgRNA* cassettes were integrated into the RECKLEEN 3 plasmid in a Golden Gate reaction using Esp3I (10,000 U/mL, NEB) following the protocol detailed above.

To assemble a plasmid targeting two genes (*araA* and *scrK*), we utilized RECKLEEN 3plus_helper sgRNA plasmids with positions 4 and 5 to target recognition sites of *araA* and *scrK*, respectively. For targeting three genes (*araA, scrK, and dhaK*), the RECKLEEN 3plus_helper gRNA plasmids with positions 4, 5a, and 5b were utilized, as illustrated in Fig. 5B. Subsequently, the *sgRNA* cassettes were integrated into the RECKLEEN 3 plasmid in a Golden Gate reaction using Esp3I (10,000 U/mL, NEB). All assemblies, including the separate *sgRNA* expression cassettes and the multi-sgRNA RECKLEEN plasmids, were carried out using *E. coli* NEB Turbo. The correct sequences were verified via Sanger sequencing, and the plasmids were finally introduced into *Kp* through electroporation as described earlier.

#### Preparation of dDNA template (dDNA)

The dDNA templates used for the lambda Red recombineering were prepared by first assembling a dDNA template plasmid using Gibson Assembly^64^. These plasmids were subsequently used as templates in PCR reactions to generate the required dDNA fragments. All dDNA template plasmids contained ~1 kbp homology arms flanking the target sequence. The part entry vector of the Marburg Collection, pMC_V_01, was used as a vector for most dDNA template plasmids^34^. Primer sequences used for assembling the dDNA template plasmids and those used in subsequent PCR reactions are provided in Supplementary Tables 4 and 5, respectively.

Following PCR amplification, residual template plasmid DNA was eliminated by adding 1 µL of DpnI (10,000 U/mL, NEB) to the 25-µL PCR reactions and incubating the mixture at 37 °C for at least 1 h. A final PCR cleanup was performed using the E.Z.N.A Cycle Pure Kit (Omega Bio-Tek), according to the manufacturer’s protocol.

#### Design of single-stranded donor DNAs

For experiments utilizing synthesized ssDNA oligonucleotides (50 b/50 b homology arms), the oligos were ordered from Merck, Germany. Single-stranded dDNAs were carefully designed to optimize the editing efficiency, based on the findings of previous studies^21,65,66^. To enhance editing efficiency, the oligonucleotides were designed to anneal to the lagging strand of the replication fork, which has previously been shown to result in up to a 100-fold improvement compared to templates targeting the leading strand^21,52^. In addition, modified oligonucleotides were used, where 2 to 4 phosphorothioate bonds were introduced between the last 2 nucleotides at the 5′- and 3′-end of the oligonucleotide to inhibit exonuclease degradation^67^. This modification renders the internucleotide linkage resistant to nuclease degradation and has been shown to enhance the stability of the oligonucleotides during recombination, thereby improving the overall editing efficiency^20,21,67^. All synthesized ssDNA sequences are listed in Supplementary Table 6.

#### RECKLEEN editing protocol

For *Kp* strains carrying the RECKLEEN plasmid, 1 mL of an overnight culture from a fresh single colony was inoculated into 50 mL of LB broth supplemented with 50 μg/mL apramycin. The culture was incubated at 30 °C until it reached an OD_600_ of approximately 0.2. At this point, 500 μM IPTG (Roth, CAS: 367-93-1) was added to induce the expression of the lambda Red recombineering operon encoded on the RECKLEEN plasmid. Induction was carried out for 2 h at 30 °C, after which the culture was prepared as electrocompetent cells following the same protocol used for the *Kp* WT.

Electroporation was performed by mixing 100 µL of the competent cells with no more than 5 µL of the dDNA. The cell-DNA mixture was transferred to a 2-mm electroporation cuvette (Bio-Rad) and electroporated at 2.5 kV, 200 Ω, and 25 µF. A control sample containing 100 µL of competent cells only without the dDNA template was prepared in parallel. Following the electroporation pulse, the cells were immediately recovered in 1 mL of SOC medium (0.5% yeast extract, 2% tryptone, 10 mM NaCl, 2.5 mM KCl, 10 mM MgCl_2_, 10 mM MgSO_4_, and 20 mM glucose) and incubated at 30 °C for 3 h.

After recovery, 100 µL of appropriate dilutions (from 10^−2^ to 10^−5^) were plated onto LB agar plates supplemented with 50 μg/ml apramycin and 50 ng/mL ATc (Alfa Aeser, 13803-65-1) to induce CRISPR/Cas9 counterselection. All experiments were performed with three biological replicates and two independent experiments. The biological replicates represent independent colonies obtained from electroporation of the RECKLEEN plasmid in *Kp*. These experiments were repeated twice on different days to ensure biological and experimental reproducibility.

### Quantification of killing efficiencies in the RECKLEEN workflow

To evaluate the killing efficiency in the RECKLEEN workflow, 100 µL of appropriate dilutions (from 10^−2^ to 10^−5^) of the control sample, which was not mixed with dDNA, were plated onto LB agar plates supplemented with 50 μg/ml apramycin and either 50 ng/ml ATc (for induced counterselection) or no ATc (for non-induced control). After incubation, colony-forming units (CFUs) were counted. The killing efficiency was calculated as follows:

${Killing\; efficiency}[ \% ]=1-\frac{\frac{{CFU}}{{mL}}{in\; presence\; of\; inducer}({with\; counterselection})}{\frac{{CFU}}{{mL}}{in\; absence\; of\; inducer}({without\; counterselection})\,}\,* 100$ to measure the percentage of killed cells upon induction of CRISPR/Cas9-mediated counterselection in the RECKLEEN system. These experiments were performed with three biological replicates representing independent colonies obtained from electroporation of *Kp* with the RECKLEEN plasmid.

### Verification of edits after RECKLEEN

The approach used for verification of edits following the RECKLEEN workflow varied based on the type of mutation introduced. However, for all mutation types, initial verification involved either colony PCR or phenotypic characterization, followed by further validation through Sanger sequencing (Supplementary Figs. 2–18). Sequencing was conducted by Microsynth Seqlab using the PCR fragments and a primer binding upstream or downstream of the modification site.

For the *wzi* deletion, colony PCR was conducted using primers that bind outside the deleted region, approximately 500 bp from the target area to screen for successful deletions. A total of twenty-five colonies were tested in each of the biological and experimental replicates. For each experiment, a randomly selected PCR fragment from one of the positive clones was Sanger sequenced. In total, we performed six different replicates. To assess the efficiency of the dDNA template targeting *wzi* (Fig. 3C), colonies exhibiting different morphologies were counted, and the data were used to calculate editing efficiencies.

For deletion and point mutation of *araA, scrK, glpK, dhak*, and *malQ*, successful editing was first screened phenotypically by streaking the obtained colonies on M9 minimal medium agar plates with either glucose or the alternative carbon source. Colonies that exhibited growth on M9 plates with glucose but not on plates with the respective secondary carbon source were considered successfully edited. A total of twenty-five colonies were tested in each of the biological and experimental replicates. Colony PCR was conducted for one positive colony and Sanger sequencing was performed to confirm the desired edit.

### Quantification of mScarlet-I signal of *Kp* reporter strains

Quantitative reporter experiments were performed as earlier described for the characterization of genetic parts of the Marburg Collection plasmid toolbox^34^. One colony was used to inoculate 5 mL LB medium. Cells were incubated overnight as precultures, and then their OD_600_ was adjusted to an OD_600_ of 1 in fresh LB. Precultures were diluted 1:100 in fresh LB to start the experiment in a Biotek Synergy H1 micro plate reader. Measurements were taken in 6 min intervals with a 3 min shaking step in double orbital mode and maximum speed applied between measurements. The OD_600_ was measured in “normal” mode with eight measurements per data point and 100 ms delay after plate movement. The mScarlet-I fluorescence was measured with a focal height of 6.5 mm, excitation and emission wavelengths of 579 nm and 616 nm, respectively, and a gain of 90. Sample data were first corrected by subtracting the mean values of three blank wells (LB medium) from the sample measurements. Growth curves were computationally synchronized by aligning the data points of each well. The mean of the OD_600_ values of the aligned growth curves of all tested replicates and independent experiments was plotted. The mScarlet-I fluorescence was normalized to the OD_600_ values.

### Complementation assays

For complementation of gene knockouts mutants (Δ*scrK*, Δ*araA*) and point mutants (*scrK*(T222A), *araA*(C57A)), plasmids for expression of WT copies of *scrK* and *araA* were generated (Supplementary Table 2). Briefly, oligonucleotides were used to PCR-amplify the WT copies of *scrK* and *araA* (Supplementary Table 8). Within the workflow of the Marburg collection, L1 plasmids for expression of the *scrK* and *araA* coding regions under control of the constitutive promoter J23105 were constructed using the parts described in Supplementary Table 1. Expression plasmids and empty control plasmid were introduced to the mutants. Complemented and control strains were plated on M9 minimal medium with either sucrose or arabinose as the sole carbon source to evaluate growth restoration.

### Whole-genome sequencing of the Δ*wzi* mutant and resequencing of the *Kp* MGH78578 WT

Total DNA for sequencing was purified using a DNeasy Blood and Tissue kit (Qiagen, Hilden, Germany). Quality control, DNA library preparation, and sequencing were performed by Azenta using Illumina NovaSeq (2 × 150 bp sequencing, ∼700 Mb paired-end reads). Mapping of the reads was performed using CLC Genomic Workbench (Qiagen, v.22.0.2) to *Kp* MGH78578 (ATCC 700721). The investigation for variants was carried out using the Basic Variant Detection, InDels, and Structural Variants tools of CLC Genomic Workbench (Qiagen, v.22.0.2) with a minimum coverage of 10, a minimum count of eight, and minimum frequency of 80% for mapped reads.

### Assessment of mutation stability and fitness after RECKLEEN editing

To evaluate the genetic stability of RECKLEEN-derived genome edits, representative *Kp* mutants were selected: a deletion mutant (Δ*wzi*), a point mutant (*wzi*(C66A)), and a chromosomal insertion strain (*rpoS*-mScarletI). Each strain was cultured in antibiotic-free LB medium and serially passaged for 10 consecutive generations (1:1000 dilution every 12 h at 37 °C, shaking). Genomic DNA was extracted from bacterial cultures harvested at the 5th and 10th passages using a DNeasy Blood and Tissue kit (Qiagen, Hilden, Germany), following the manufacturer’s protocol. To confirm the retention of the engineered edit, locus-specific primers flanking the target site were used to amplify the region by PCR. Amplicons were Sanger sequenced (Microsynth Seqlab) and analyzed using SnapGene® 5.0.8 software to detect any reversion events or secondary mutations. To assess growth dynamics and relative fitness, OD₆₀₀ were recorded over time using a Biotek Synergy H1 micro plate reader, as previously described. The mean of the OD_600_ values of the aligned growth curves of all tested replicates and independent experiments was plotted.

### Statistics and reproducibility

Data are shown as mean values ± SEM for at least three biologically independent replicates. Each replicate originated from an independent colony obtained after electroporation of *K. pneumoniae* with the RECKLEEN plasmid. Data, in Figs. 1B, C, 3D, 4B, 5b, and S7, originated from three biologically independent replicates (*n* = 3). Individual replicates are shown, and the error bars represent the standard deviation from the mean. For proof-of-concept applications, including deletions (Fig. 3B), dDNA type (Fig. 3C), point mutations (Fig. 4C), and chromosomal DNA integrations (Fig. 4D), six independent biological replicates were performed (*n* = 6, three per experiment, across two independent experiments to ensure both biological and experimental reproducibility). Individual replicates are shown as circles; filled and open symbols denote independent experiments. Error bars indicate standard deviation from the mean. Twenty-five colonies (sample size = 25) were screened per replicate, yielding a total of 150 colonies per target. Plasmid curing data originates from three independent biological replicates after confirming the editing. Twenty-five colonies were analyzed for each biological replicate. Individual replicates are shown, and the error bars represent the standard deviation from the mean. Prism 8.4.3 (GraphPad, La Jolla, USA) was used.

### Reporting summary

Further information on research design is available in the Nature Portfolio Reporting Summary linked to this article.

## Supplementary information

Supplementary Information

Description of additional supplementary files

Supplementary Data 1

Supplementary Data 2

Supplementary Data 3

Supplementary Data 4

Supplementary Data 5

Supplementary Data 6

Supplementary Data 7

Reporting Summary

## Data Availability

The maps of the RECKLEEN plasmids are available as Supplementary Data 1. Plasmids are available through Addgene (plasmid IDs 233456 to 233466) and are available from the authors upon reasonable request. Descriptions of plasmid assemblies are provided in Supplementary Table 1. Strains that have been used and constructed in this study are provided in Supplementary Table 2. Sequences of all oligonucleotides used in this study are provided in Supplementary Tables 3–6. All supplementary tables are available in Supplementary Data 2. Data used to generate the figures shown in this publication is provided as Supplementary Data 3–7. Bacterial sequencing data generated in this study have been deposited in the BioStudies database under the accession number E-MTAB-15474. Any other relevant data is available from the corresponding author upon reasonable request.
