# Supplementary material for: RECKLEEN is a lambda Red/CRISPR-Cas9 based single plasmid platform for enhanced genome editing in Klebsiella pneumoniae
Source: Commun Biol. 2025 Oct 30;8:1509. doi: 10.1038/s42003-025-08934-8 (PMC12575629; doi:10.1038/s42003-025-08934-8)
Supplement: Supplementary file 1 — Supplementary Information [file 42003_2025_8934_MOESM1_ESM.pdf]

Supplementary information to:

RECKLEEN is a lambda Red/CRISPR-Cas9 based single plasmid platform for enhanced genome editing in *Klebsiella pneumoniae*

Eslam M. Elsayed[1,2,3], Daniel Stukenberg[1,#], Doreen Meier[1,2], Bernd Schmeck[1,4,5], and Anke Becker[1,2*]

[1]Center for Synthetic Microbiology (SYNMIKRO), Philipps-Universität Marburg, Marburg, Germany

[2]Department of Biology, Philipps-Universität Marburg, Marburg, Germany

[3]Department of Microbiology and Immunology, Faculty of Pharmacy, Zagazig University, Zagazig, Egypt

[4]Institute for Lung Research, Universities of Giessen and Marburg Lung Center, German Center for Lung Research (DZL), Philipps-University Marburg, Marburg, Germany

[5]Department of Medicine, Pulmonary and Critical Care Medicine, University Medical Center Marburg, Universities of Giessen and Marburg Lung Center, Philipps-University Marburg, Marburg, Germany

[#]Current address: Department of Biology, Technical University Darmstadt, Darmstadt, Germany

[*]For correspondence: anke.becker@synmikro.uni-marburg.de

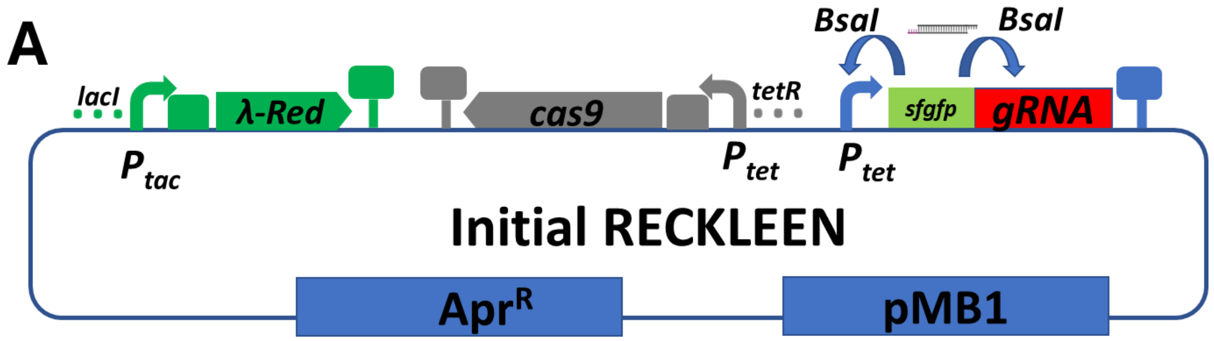

B

- Use of engineered variants of P_{tet} ( ).
- Use of different ribosome binding sites (RBS).
- Use of different origins of replication (Ori).
- Adding a SsrA consensus tag to the C-terminus of Cas9.
- Addition of Anti-CRISPR (ACRIIA4) transcription unit.

Supplementary Figure 1. A) Schematic design of the initial plasmid constructed to prototype the RECKLEEN system in *Kp*. The plasmid carries three different transcriptional units: the lambda Red operon (*gam, exo, beta*) under control of the inducible P_{tac} promoter, as well as the *cas9* and *sgRNA* transcription units, both under control of the inducible P_{tet} promoter. The vector backbone carries the *lacI* and *tetR* genes encoding the transcription regulators of these promoters and an apramycin resistance gene marker and a pMB1 origin of replication. This plasmid can be customized by replacing a sfGFP transcription unit in front of the *sgRNA* scaffold-encoding sequence with a 20-nt guide spacer sequence through Golden Gate assembly using the BsaI restriction enzyme. **B)** Applied strategies to address the possible toxic effects of a hypothesized leaky expressing the CRISPR/Cas9 part of the RECKLEEN system targeting the genomic sequence. Anti-CRISPR (AcrIIA4) mitigates the lethal effect of the CRISPR/Cas9 part of the RECKLEEN system and enables electroporation of the plasmids with *sgRNA* targeting the genomic sequence of *Kp*. The plasmid construct has been redesigned to contain an *acrIIA4* transcription unit, constitutively expressed under the control of the constitutive promoter J23100.

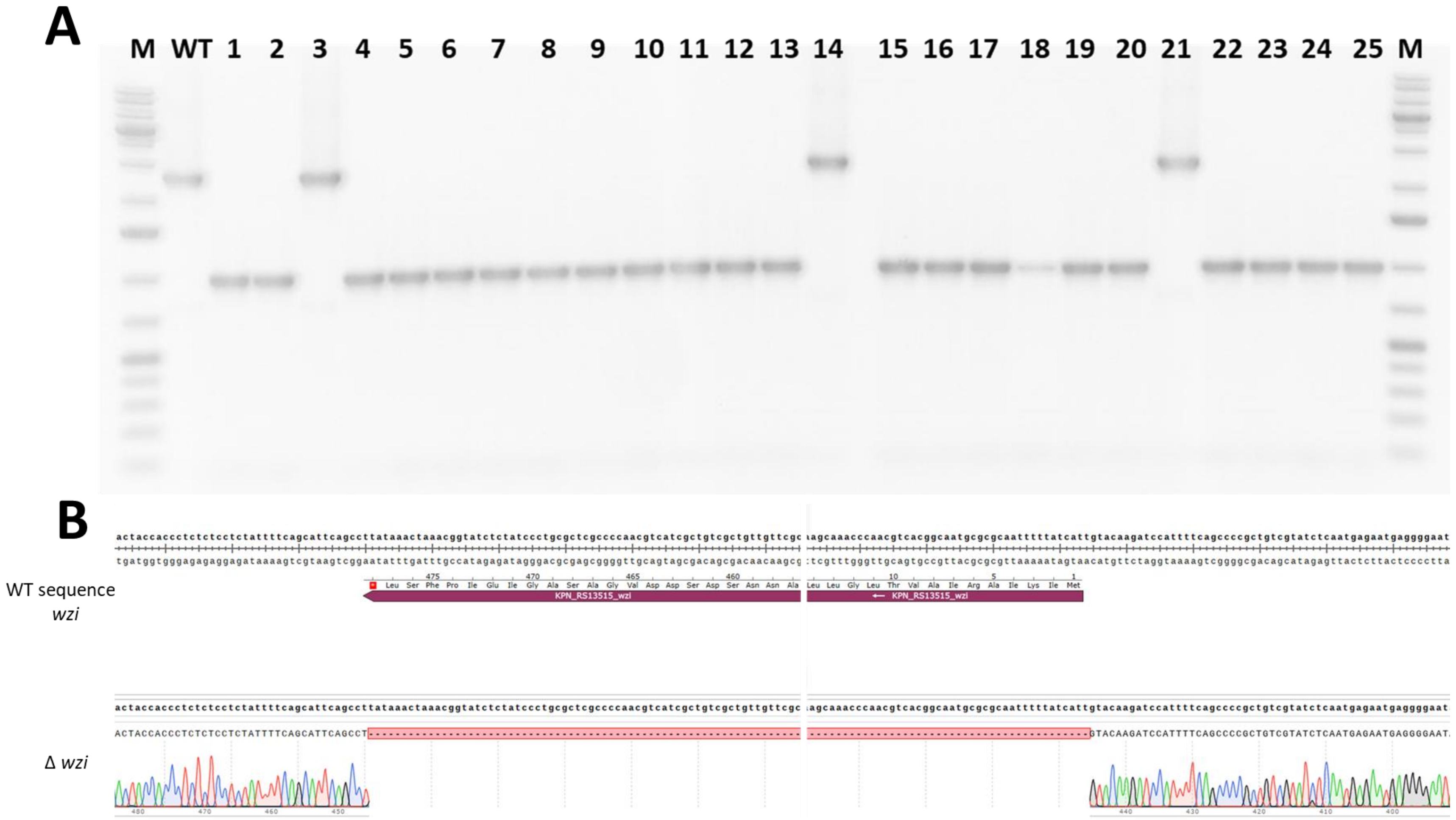

Supplementary Figure 2. Evaluation of *wzi* deletion in *Kp* ATCC700721. A) Colony PCR results demonstrating the deletion of the *wzi* gene using primers that bind approximately 500 bp outside the deleted region. Lane M represents the marker; WT corresponds to the wild-type strain, which serves as a positive control for the unmodified *wzi* locus. All relevant experiments are available in Supplementary Data 3. **B)** Sanger sequencing of PCR products confirmed the deletion of *wzi*. Sequencing was performed by Microsynth Seqlab, using PCR fragments and primers binding upstream or downstream of the targeted modification. In total, six PCR fragments were sequenced. The wild-type (WT) sequence is shown alongside the sequences of the replicates. Alignment of sequencing files was conducted using SnapGene® 5.0.8, with screenshots provided to illustrate the deletion of the *wzi* coding sequence. All relevant sequencing files are available in the Supplementary Data 6.

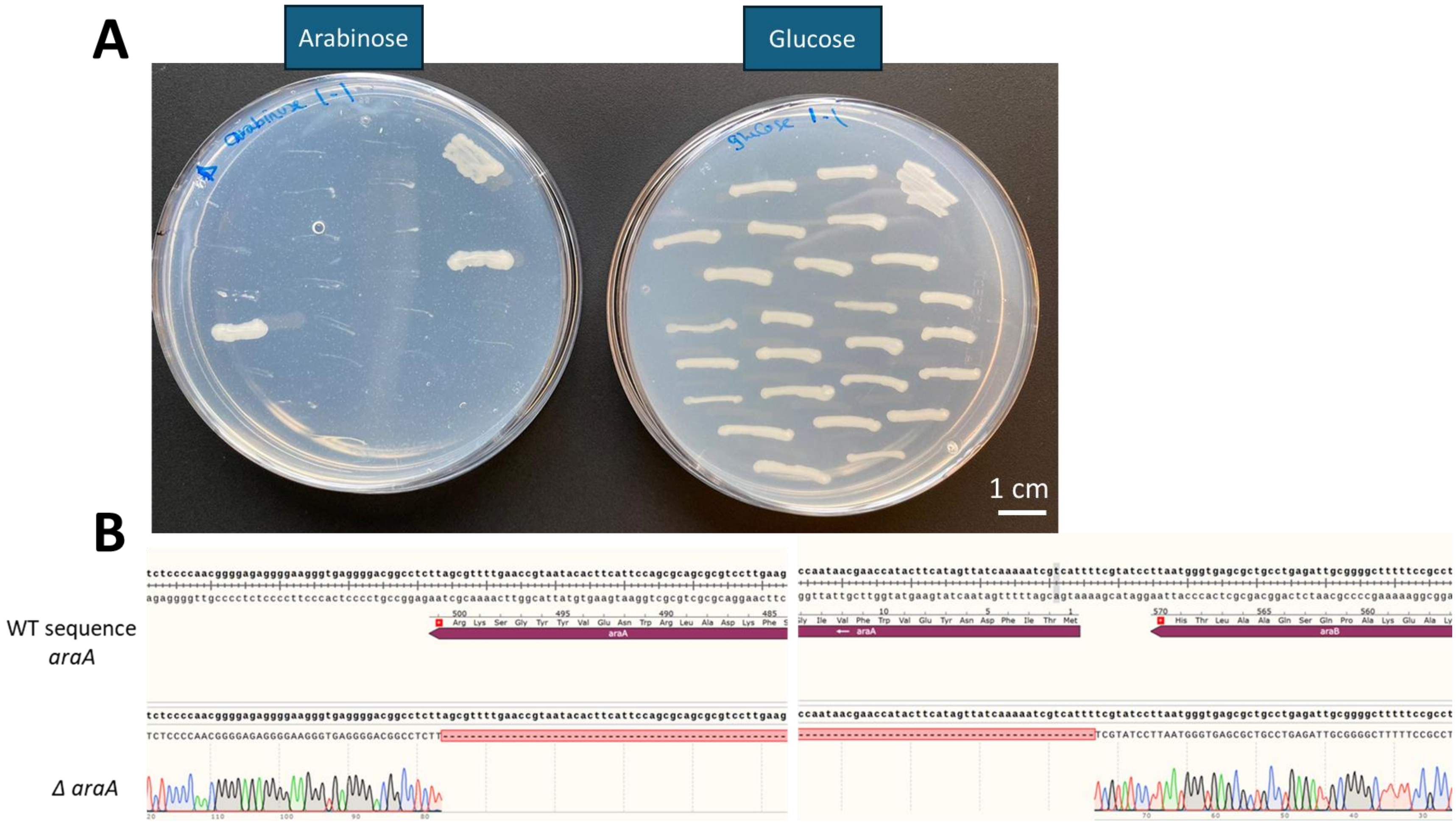

Supplementary Figure 3. Evaluation of *araA* deletion. A) Phenotypic characterization of *araA* deletion. The deletion of the *araA* gene was screened phenotypically by streaking the obtained colonies on M9 minimal medium agar plates with either glucose or the alternative carbon source (arabinose in this case). Colonies that grew on M9 plates with glucose but failed to grow on arabinose plates were classified as successfully edited. All relevant experiments are available in Supplementary Data 4. **B)** Sanger sequencing of PCR products confirmed the deletion of *araA*. Sequencing was performed by Microsynth Seqlab, using PCR fragments and primers binding upstream or downstream of the targeted modification. In total, six PCR fragments were sequenced. The wild-type (WT) sequence is shown alongside the sequences of the replicates. Alignment of sequencing files was conducted using SnapGene® 5.0.8, with screenshots provided to illustrate the deletion of the *araA* coding sequence. All relevant sequencing files are available in Supplementary Data 6.

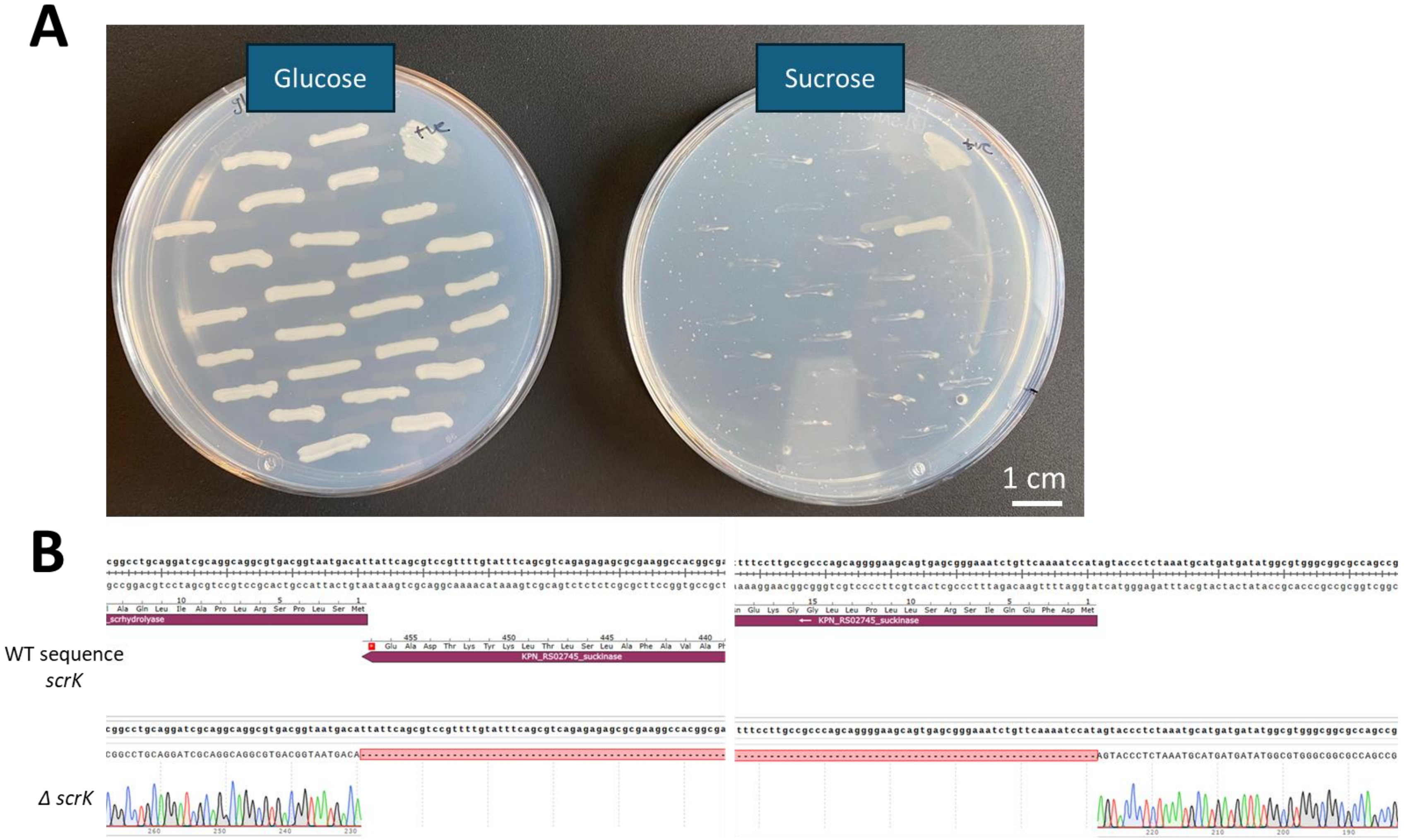

Supplementary Figure 4. Evaluation of *scrK* deletion. A) Phenotypic characterization of *scrK* deletion. The deletion of the *scrK* gene was screened phenotypically by streaking the obtained colonies on M9 minimal medium agar plates with either glucose or the alternative carbon source (sucrose in this case). Colonies that grew on M9 plates with glucose but failed to grow on sucrose plates were classified as successfully edited. All relevant experiments are available in Supplementary Data 4. **B)** Sanger sequencing of PCR products confirmed the deletion of *scrK*. Sequencing was performed by Microsynth Seqlab, using PCR fragments and primers binding upstream or downstream of the targeted modification. In total, six PCR fragments were sequenced. The wild-type (WT) sequence is shown alongside the sequences of the replicates. Alignment of sequencing files was conducted using SnapGene® 5.0.8, with screenshots provided to illustrate the deletion of the *scrK* coding sequence. All relevant sequencing files are available in Supplementary Data 6.

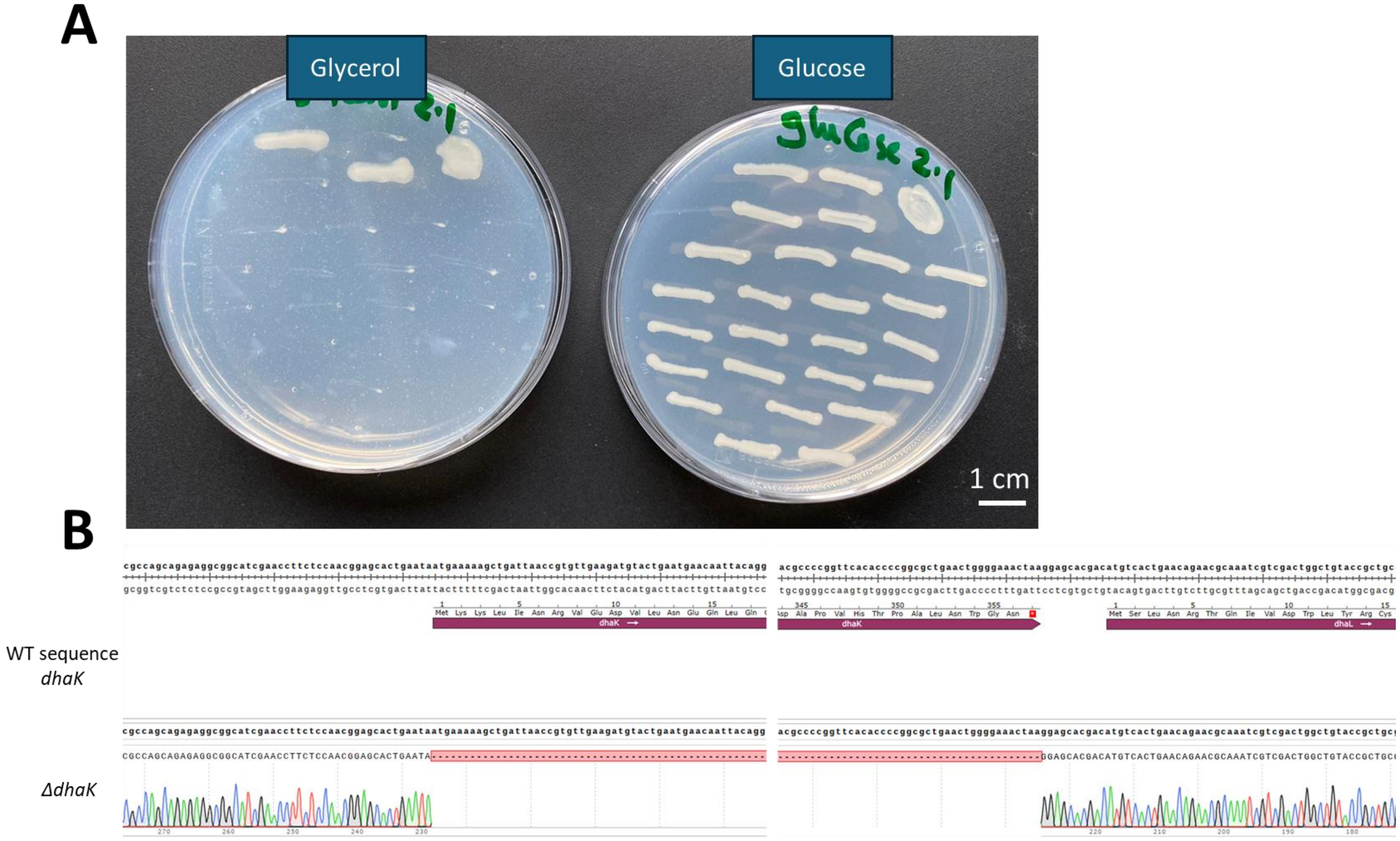

Supplementary Figure 5. Evaluation of *dhaK* deletion. A) Phenotypic characterization of *dhaK* deletion. The deletion of the *dhaK* gene was screened phenotypically by streaking the obtained colonies on M9 minimal medium agar plates with either glucose or the alternative carbon source (glycerol in this case). Colonies that grew on M9 plates with glucose but failed to grow on glycerol plates were classified as successfully edited. All relevant experiments are available in Supplementary Data 4. **B)** Sanger sequencing of PCR products confirmed the deletion of *dhaK*. Sequencing was performed by Microsynth Seqlab, using PCR fragments and primers binding upstream or downstream of the targeted modification. In total, six PCR fragments were sequenced. The wild-type (WT) sequence is shown alongside the sequences of the replicates. Alignment of sequencing files was conducted using SnapGene® 5.0.8, with screenshots provided to illustrate the deletion of the *dhaK* coding sequence. All relevant sequencing files are available in Supplementary Data 6.

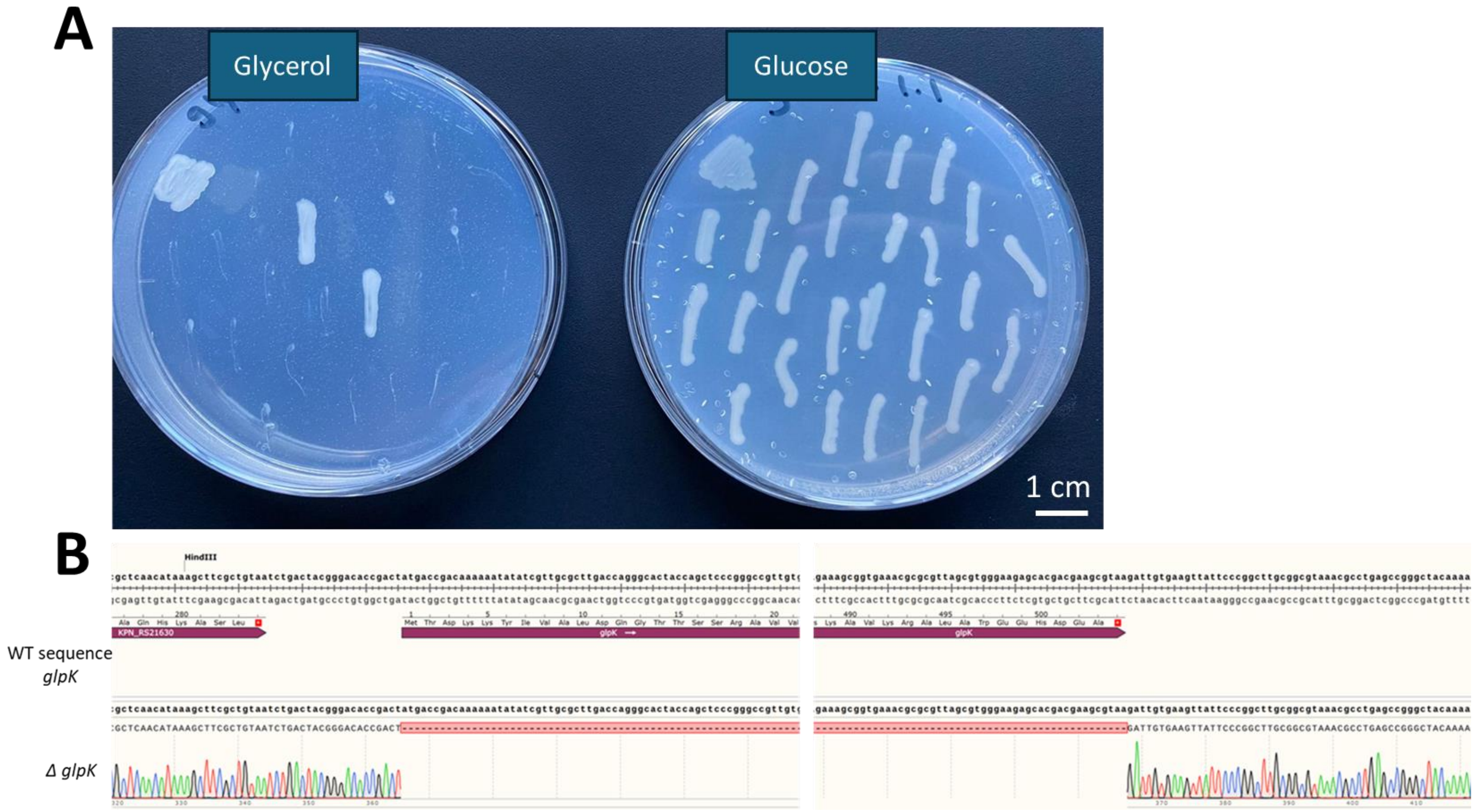

Supplementary Figure 6. Evaluation of *glpK* deletion. A) Phenotypic characterization of *glpK* deletion. The deletion of the *glpK* gene was screened phenotypically by streaking the obtained colonies on M9 minimal medium agar plates with either glucose or the alternative carbon source (glycerol in this case). Colonies that grew on M9 plates with glucose but failed to grow on glycerol plates were classified as successfully edited. All relevant experiments are available in Supplementary Data 4. **B)** Sanger sequencing of PCR products confirmed the deletion of *glpK*. Sequencing was performed by Microsynth Seqlab, using PCR fragments and primers binding upstream or downstream of the targeted modification. In total, six PCR fragments were sequenced. The wild-type (WT) sequence is shown alongside the sequences of the replicates. Alignment of sequencing files was conducted using SnapGene® 5.0.8, with screenshots provided to illustrate the deletion of the *glpK* coding sequence. All relevant sequencing files are available in Supplementary Data 6.

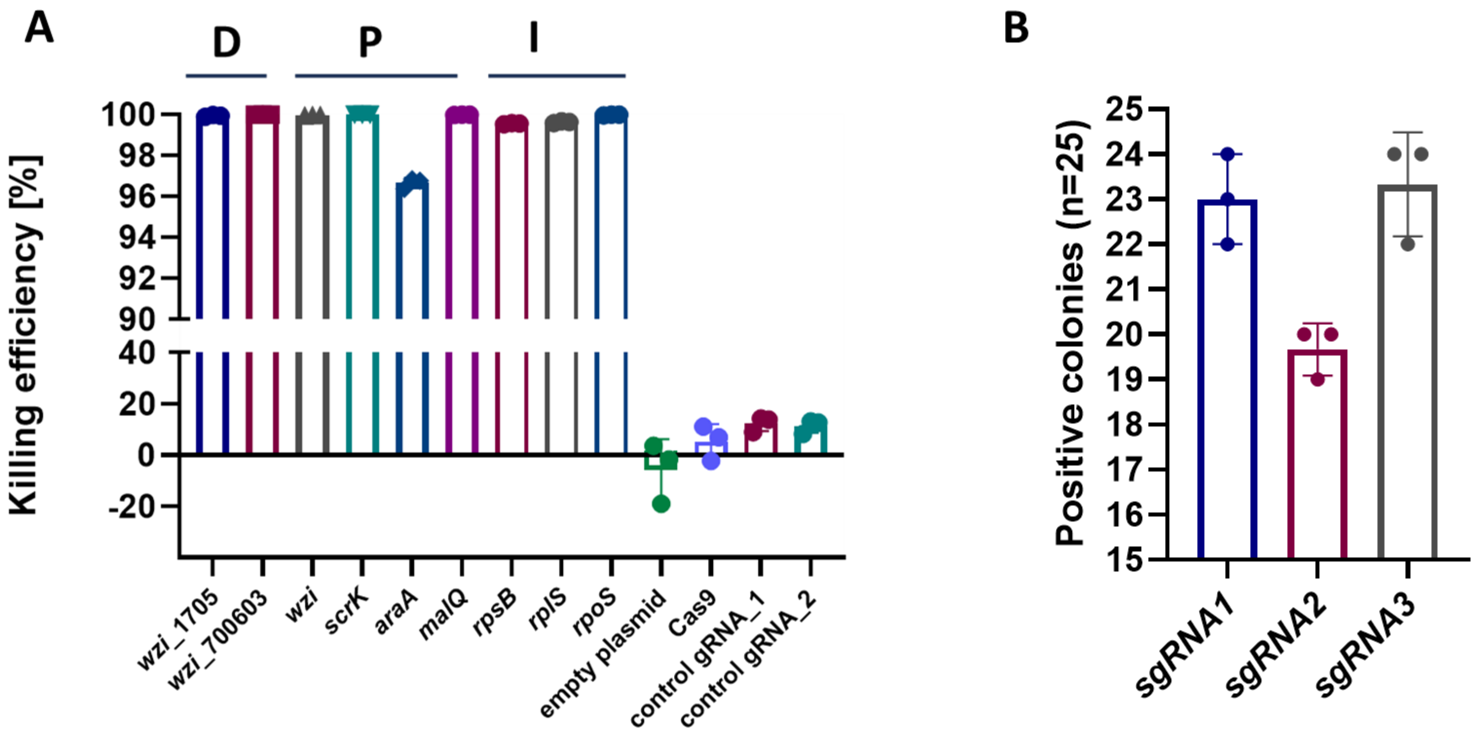

Supplementary Figure 7. (A) Killing efficiency for various *sgRNA* for the deletion (D), point mutation (P), and DNA integration (I) of different target genes. The killing efficiency was measured after induction with ATc. The killing efficiency was calculated as follows: $Killing\ efficiency\ [\%] = 1 - \frac{\frac{CFU}{mL}\ in\ presence\ of\ inducer(\ with\ counterselection)}{\frac{CFU}{mL} in absence\ of\ inducer\ (without\ counterselection)}$ * 100 to measure the percentage of killed cells upon induction of the system. **(B) Impact of various sgRNA positions on the editing efficiencies of RECKLEEN.** Three distinct sgRNAs targeting the *wzi* coding sequence at nucleotide positions 359 (sgRNA1), 790 (sgRNA2), and 1311 (sgRNA3) (Supplementary Table 3) were employed to assess the effect of different sgRNA positions on the editing efficiencies of the RECKLEEN 1 platform. Data, in A and B, represents the mean of three biological replicates (*n* = 3), individual replicates are shown, and the error bars represent the standard deviation from the mean.

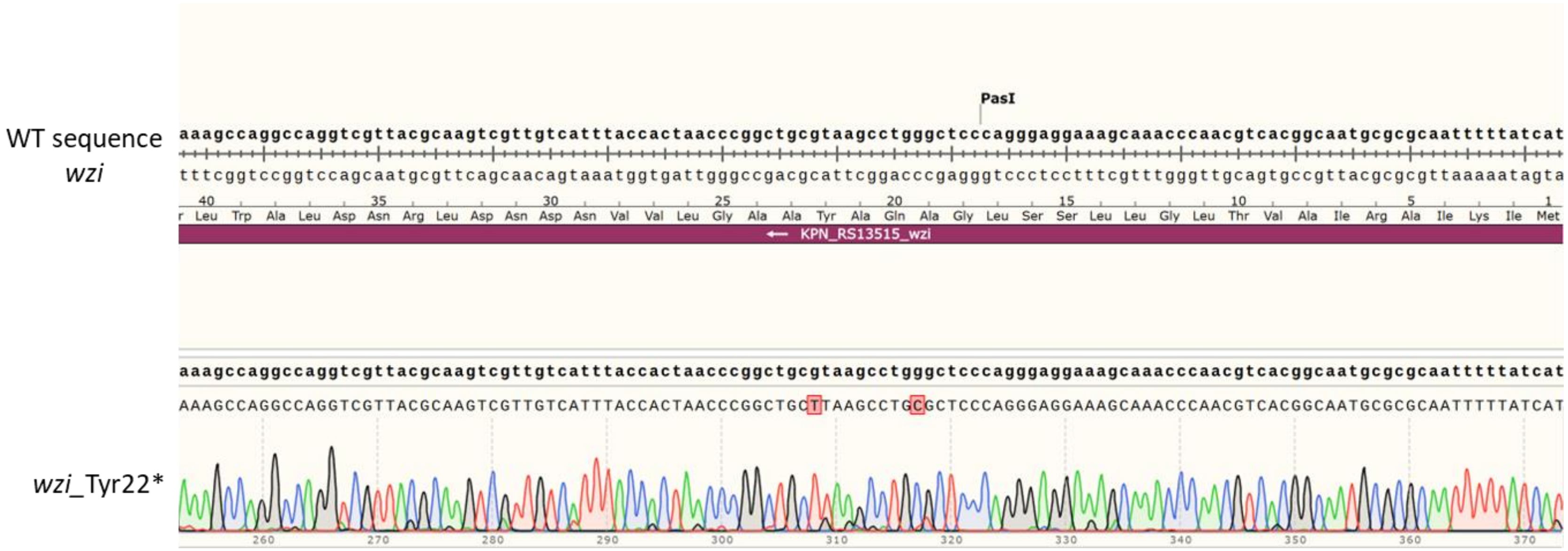

Supplementary Figure 8. Evaluation of *wzi*_C66A point mutation. Sanger sequencing was performed through Microsynth Seqlab using PCR fragments and a primer binding upstream or downstream of the respective modification. In total, six different PCR fragments were sequenced. The wild-type (WT) sequence is shown alongside the sequences of the replicates for comparison. All sequencing alignments were performed using SnapGene® 5.0.8, with screenshots demonstrating the successful incorporation of the C66A point mutation. Additionally, guanine (G57) of *wzi* was edited into cytosine (C) as a silent alanine mutation besides the previously designed point mutation to overcome the mismatch repair mechanism. The sequencing files are provided in Supplementary Data 6.

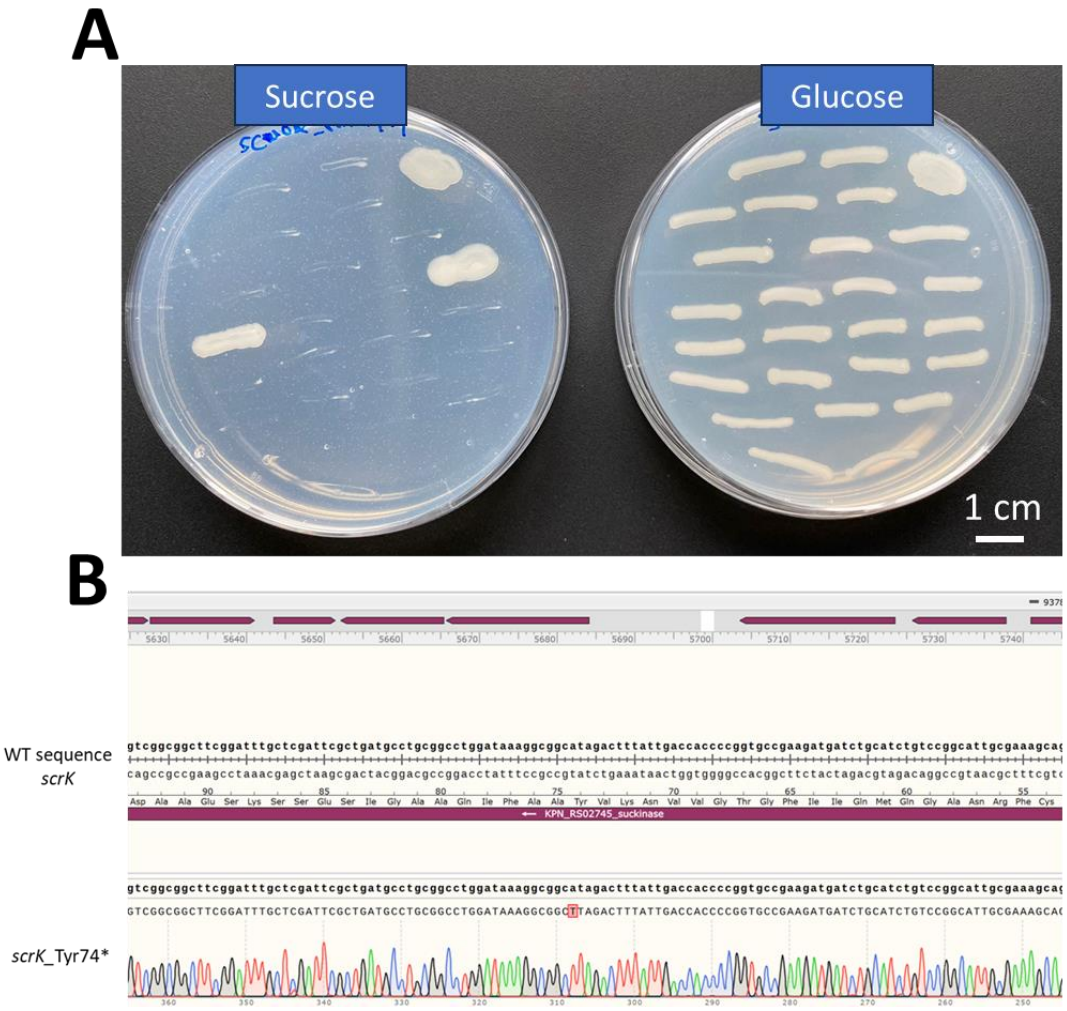

Supplementary Figure 9. Evaluation of *scrK*_T222A point mutation. A) Phenotypic characterization of *scrK*_T222A point mutation. The point mutation of the *scrK* gene was screened phenotypically by streaking the obtained colonies on M9 minimal medium agar plates with either glucose or the alternative carbon source (sucrose in this case). Colonies that grew on M9 plates with glucose but failed to grow on sucrose plates were classified as successfully edited. All relevant experimental data are available in Supplementary Data 4. **B)** Sanger sequencing of PCR products confirmed the point mutation of *scrK*. Sequencing was performed by Microsynth Seqlab, using PCR fragments and primers binding upstream or downstream of the targeted modification. In total, six PCR fragments were sequenced. The wild-type (WT) sequence is shown alongside the sequences of the replicates. Alignment of sequencing files was conducted using SnapGene® 5.0.8, with screenshots provided to illustrate the point mutation. All relevant sequencing files are available in Supplementary Data 6.

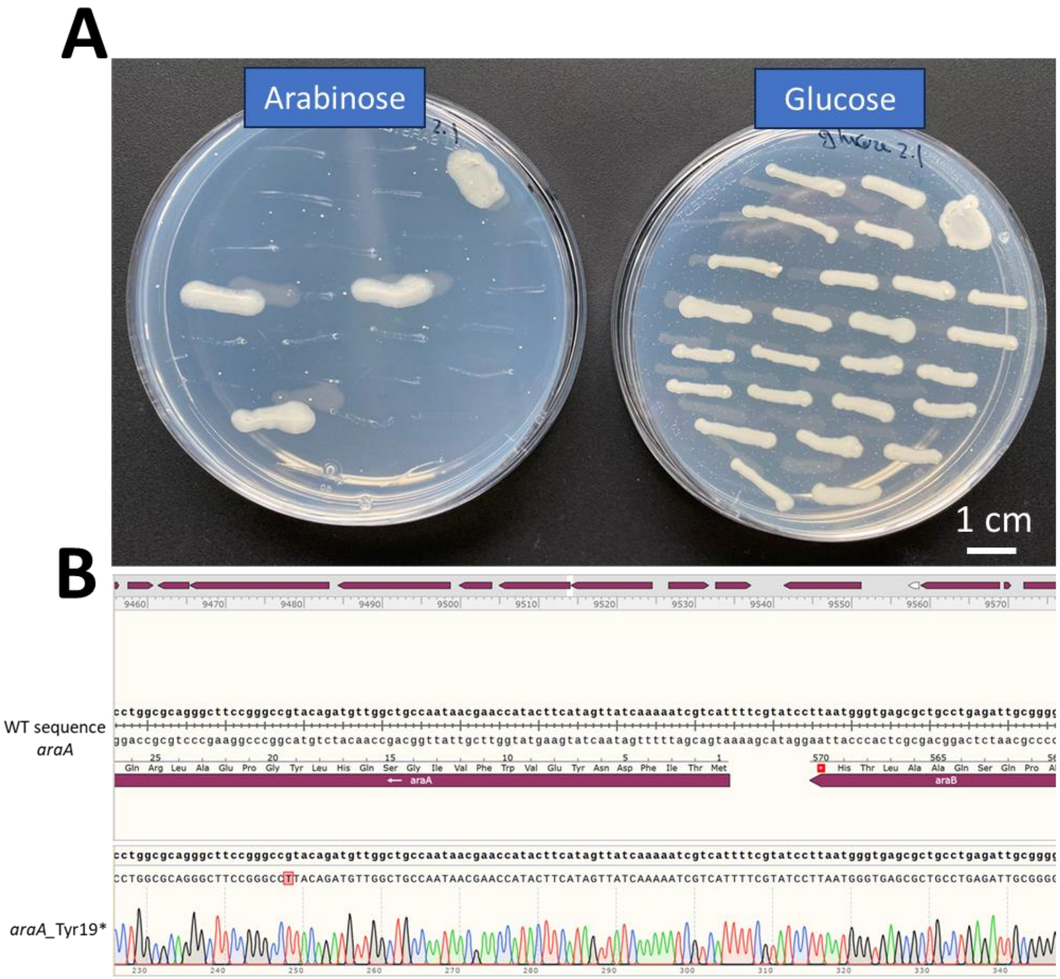

Supplementary Figure 10. Evaluation of *araA*_C57A point mutation. A) Phenotypic characterization of *araA*_C57A point mutation. The point mutation of the *araA* gene was screened phenotypically by streaking the obtained colonies on M9 minimal medium agar plates with either glucose or the alternative carbon source (arabinose in this case). Colonies that grew on M9 plates with glucose but failed to grow on arabinose plates were classified as successfully edited. All relevant experimental data are available in Supplementary Data 4. **B)** Sanger sequencing of PCR products confirmed the point mutation of *araA*. Sequencing was performed by Microsynth Seqlab, using PCR fragments and primers binding upstream or downstream of the targeted modification. In total, six PCR fragments were sequenced. The wild-type (WT) sequence is shown alongside the sequences of the replicates. Alignment of sequencing files was conducted using SnapGene® 5.0.8, with screenshots provided to illustrate the point mutation. All relevant sequencing files are provided in Supplementary Data 6.

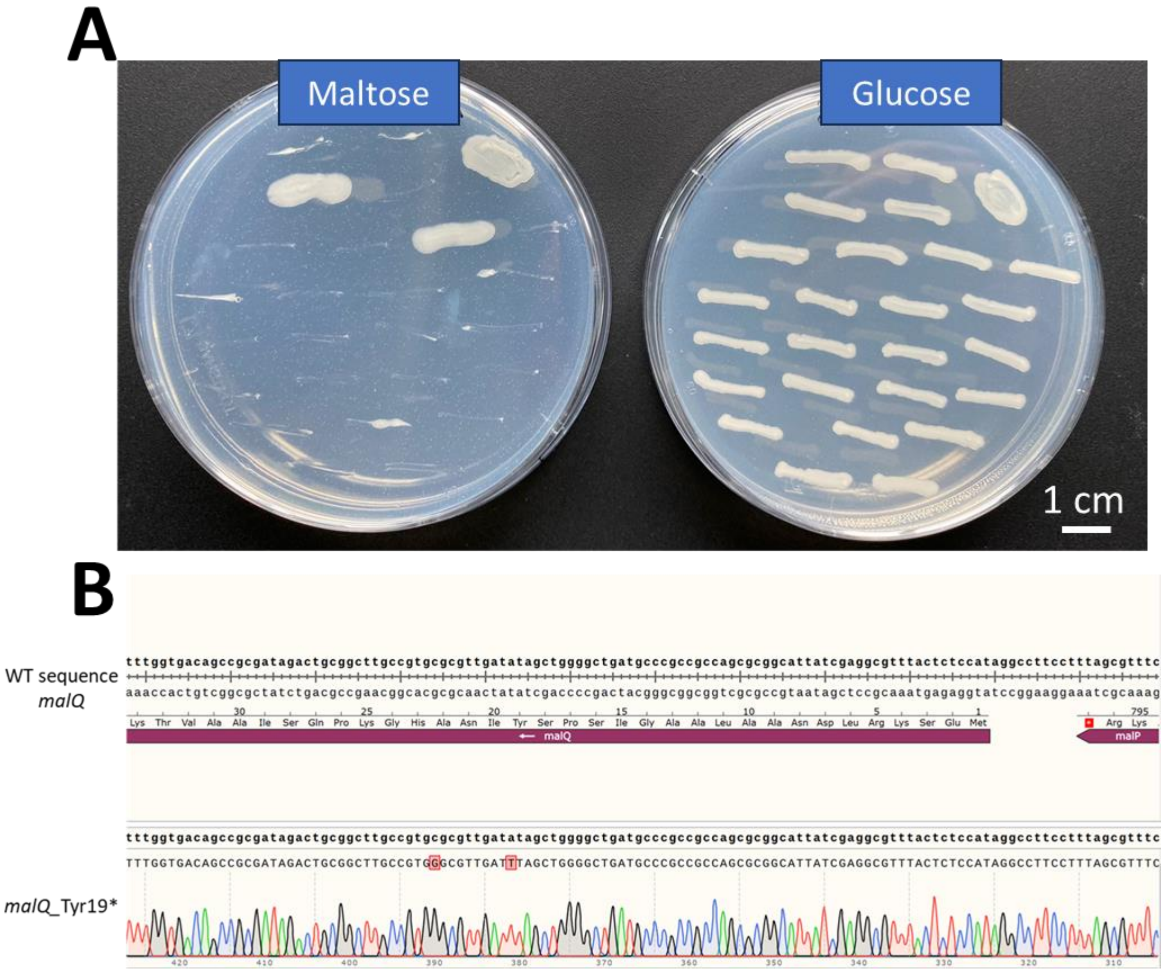

Supplementary Figure 11. Evaluation of *malQ*_T57A point mutation. A) Phenotypic characterization of *malQ*_T57A point mutation. The point mutation of the *malQ* gene was screened phenotypically by streaking the obtained colonies on M9 minimal medium agar plates with either glucose or the alternative carbon source (maltose in this case). Colonies that grew on M9 plates with glucose but failed to grow on maltose plates were classified as successfully edited. All relevant experimental data are available in Supplementary Data 4. **B)** Sanger sequencing of PCR products confirmed the point mutation of *malQ*. Sequencing was performed by Microsynth Seqlab, using PCR fragments and primers binding upstream or downstream of the targeted modification. In total, six PCR fragments were sequenced. The wild-type (WT) sequence is shown alongside the sequences of the replicates. Alignment of sequencing files was conducted using SnapGene® 5.0.8, with screenshots provided to illustrate the point mutation. Additionally, Guanine (G66) of malQ was edited into Cytosine (C) as a silent alanine mutation besides the previously designed point mutation to overcome the mismatch repair mechanism. The sequencing files are provided in Supplementary Data 6.

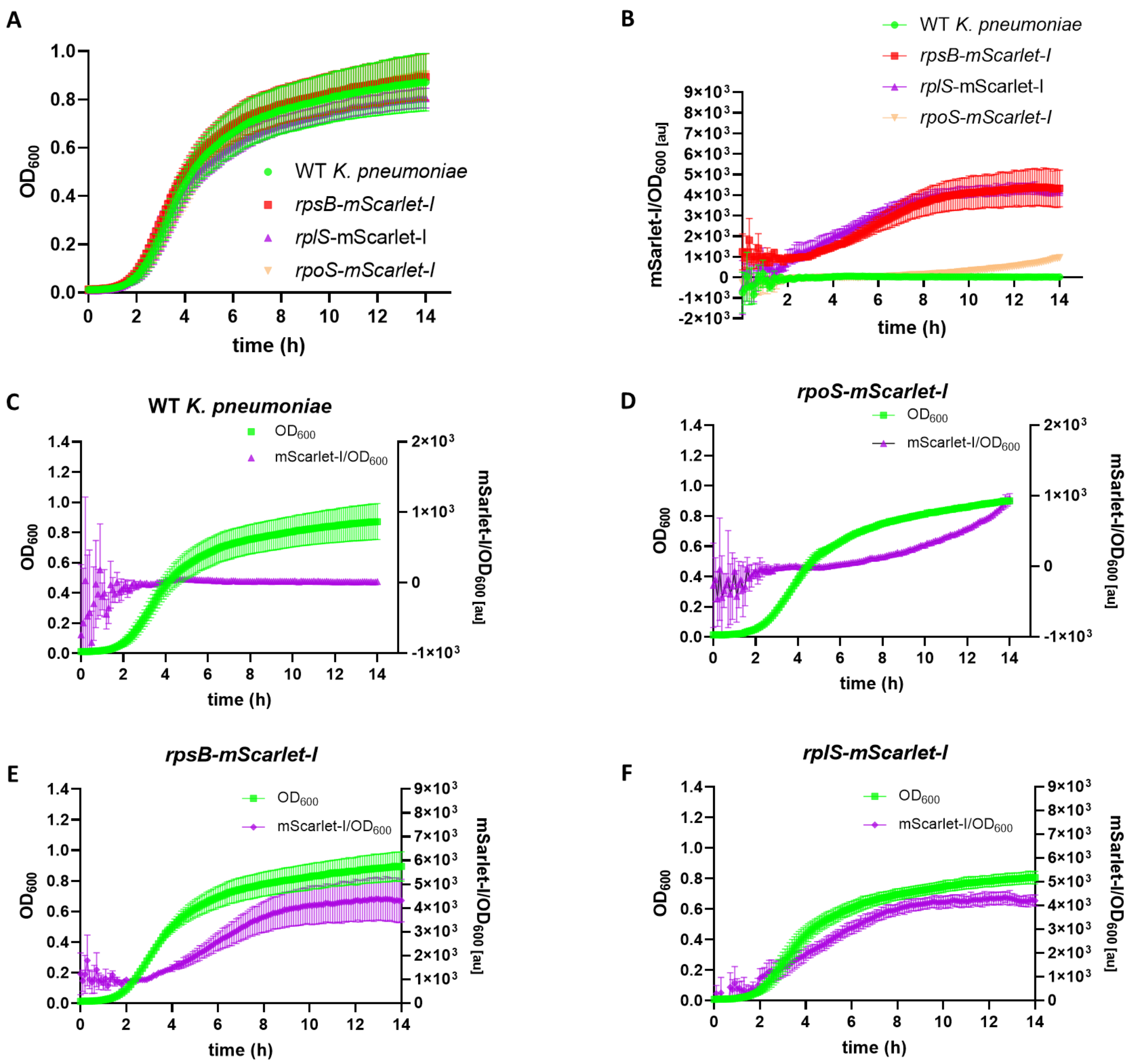

Supplementary Figure 12. Evaluation of DNA integrations. A) Growth Curves of WT *Kp* and strains with integrated mScarlet-I fused to the 3' end of *rpsB, rplS*, and *rpoS.* The integration of the mScarlet-I didn't affect the growth of the *Kp* strains**. B)** Normalized m-Scarlet-I signal of WT *Kp* and strains with integrated mScarlet-I fused to the 3' end of *rpsB, rplS*, and *rpoS.* **C)** Growth curve (shown in green) and normalized mScarlet-I signal (shown in violet) of WT *Kp***. D)** Growth curve (shown in green) and normalized mScarlet-I signal (shown in violet) of *Kp* with integrated mScarlet-I fused to the 3' end of *rpoS.* **E)** Growth curve (shown in green) and normalized mScarlet-I signal (shown in violet) of with integrated mScarlet-I fused to the 3' end of *rpsB.* **F)** Growth curve (shown in green) and normalized mScarlet-I signal (shown in violet) of with integrated mScarlet-I fused to the 3' end of *rplS*. Data represents the mean of three biological replicates and two independent experiments, and the error bars represent the standard deviation from the mean. Similar expression patterns of the two ribosomal genes (*rpsB* and *rplS*) during the bacterial growth. Both have been significantly upregulated after 2 hrs of growth. This upregulation continued till 8 hours with a steady state increase. Then, it reached a stable level after 8 hrs. On the other hand, the stress sigma factor *rpoS* was significantly upregulated during the stationary phase after almost 8 hrs of bacterial growth.

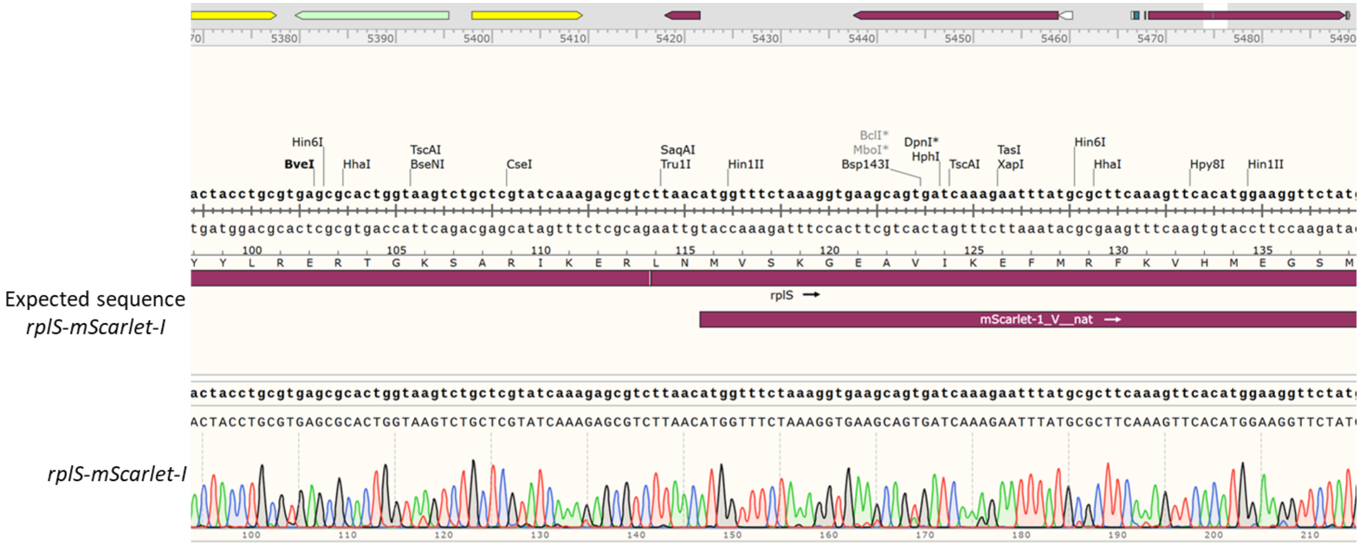

Supplementary Figure 13. Evaluation of *mScarlet-I integration fusion to* the 3' end of *rplS*. Sanger sequencing was performed through Microsynth Seqlab using PCR fragments and a primer binding upstream or downstream of the respective modification. In total, six different PCR fragments were sequenced. The expected sequence is shown alongside the sequences of the replicates for comparison. All sequencing alignments were performed using SnapGene® 5.0.8, with screenshots demonstrating the successful DNA integration. The sequencing files are provided in Supplementary Data 6.

Expected sequence
rpsB-mScarlet-I

rpsB-mScarlet-I

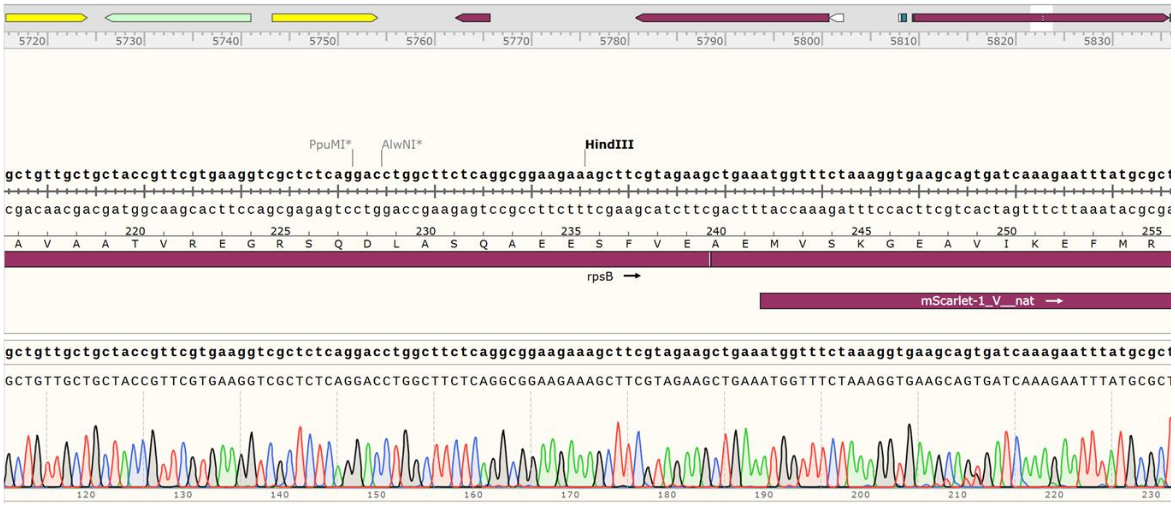

Supplementary Figure 14. **Evaluation of *mScarlet-I* integration fusion to the 3' end of *rpsB*.** Sanger sequencing was performed through Microsynth Seqlab using PCR fragments and a primer binding upstream or downstream of the respective modification. In total, six different PCR fragments were sequenced. The expected sequence is shown alongside the sequences of the replicates for comparison. All sequencing alignments were performed using SnapGene® 5.0.8, with screenshots demonstrating the successful DNA integration. The sequencing files are provided in Supplementary Data 6.

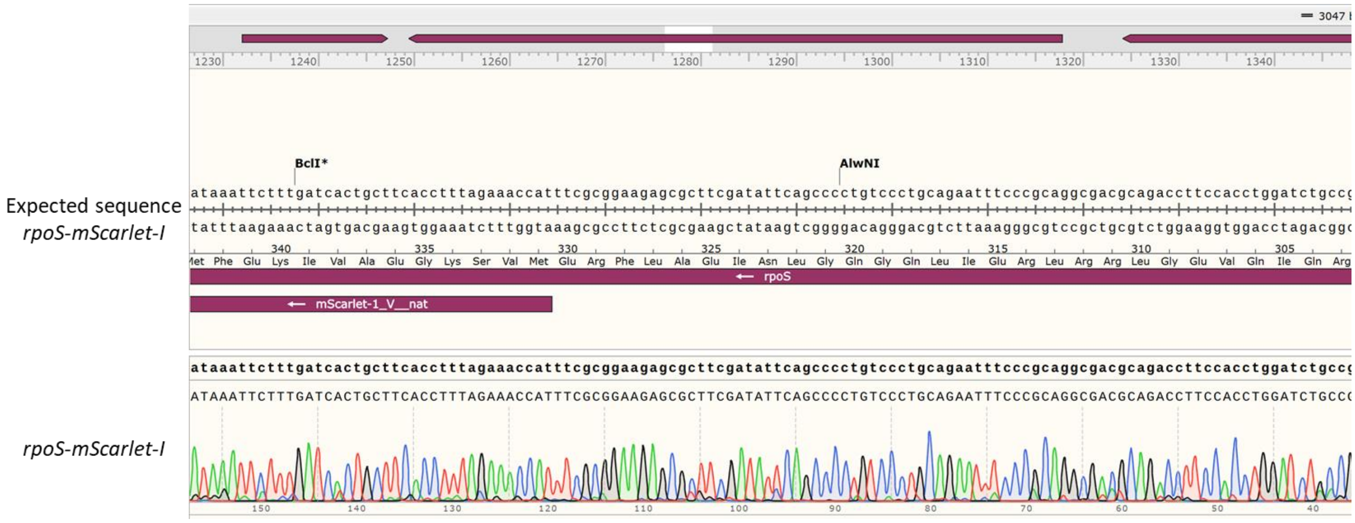

Supplementary Figure 15. Evaluation of *mScarlet-I* integration fusion to the 3' end of *rpoS*. Sanger sequencing was performed through Microsynth Seqlab using PCR fragments and a primer binding upstream or downstream of the respective modification. In total, six different PCR fragments were sequenced. The expected sequence is shown alongside the sequences of the replicates for comparison. All sequencing alignments were performed using SnapGene® 5.0.8, with screenshots demonstrating the successful DNA integration. The sequencing files are provided in Supplementary Data 6.

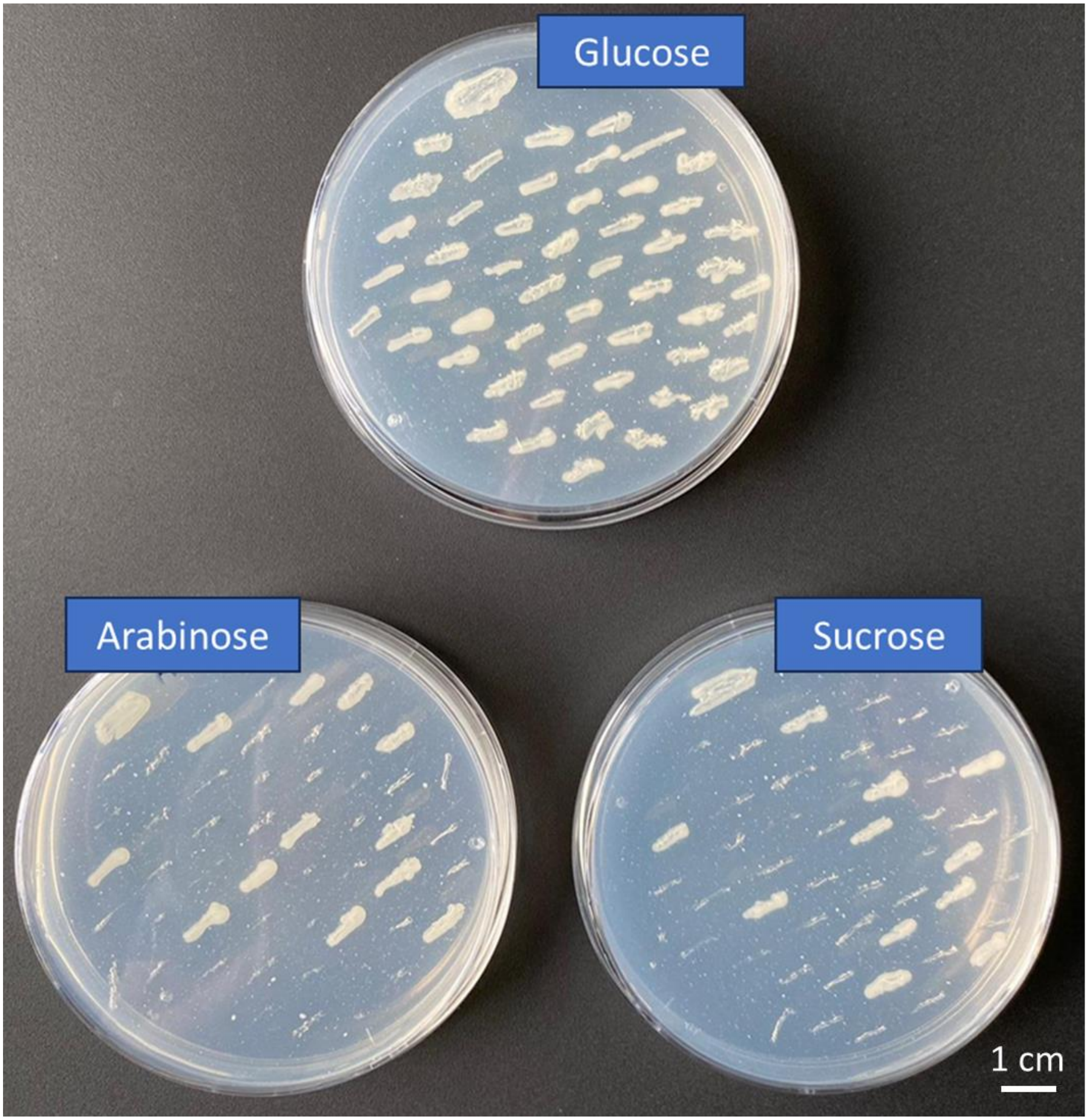

Supplementary Figure 16. Evaluation of multi-target deletions of *araA* and *scrK* using REKLEEN 3 platform. Phenotypic characterization of *araA* and *scrK* deletions. The deletion was screened phenotypically by streaking the obtained colonies on M9 minimal medium agar plates with either glucose or the alternative carbon source (arabinose and sucrose in this case). Colonies that grew on M9 plates with glucose but failed to grow on the respective secondary carbon source plates were classified as successfully edited. In total, fifty colonies were tested. Sanger sequencing was then harnessed to confirm the deletion of the two genes. The sequencing files are provided in supplementary Data 6. The desired deletion of the target genes was detected in 72% of the randomly selected colonies. Around 4% of the selected colonies had *araA* but not *scrK* deleted, while 6% showed deletion of *scrK* but not *araA*.

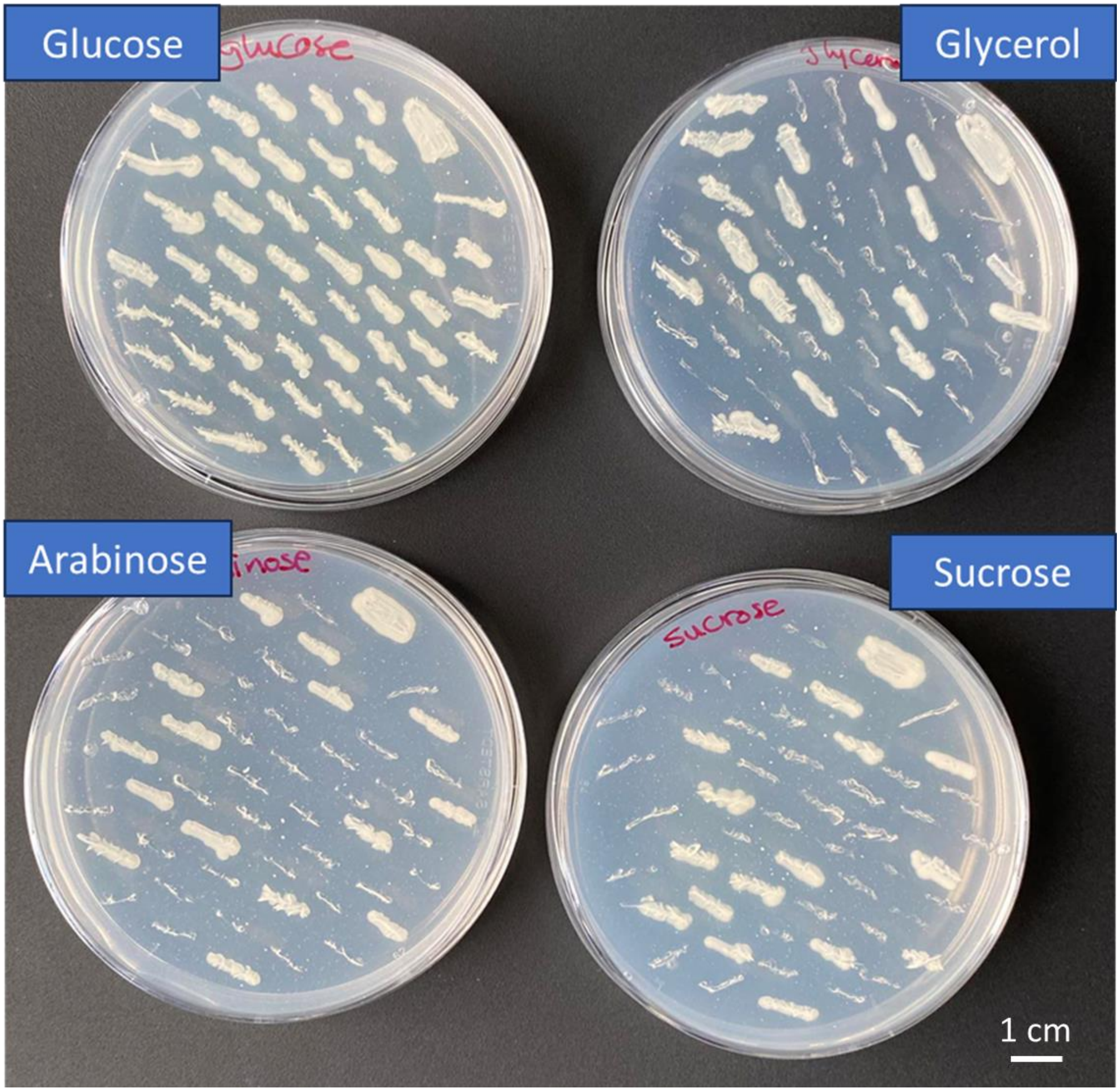

Supplementary Figure 17. Evaluation of multi-target deletions of *araA, dhaK* and *scrK* using RECKLEEN 3 platform. Phenotypic characterization of *araA, dhaK* and *scrK* deletions. The deletion was screened phenotypically by streaking the obtained colonies on M9 minimal medium agar plates with either glucose or the alternative carbon source (arabinose, glycerol, and sucrose in this case). Colonies that grew on M9 plates with glucose but failed to grow on the respective secondary carbon source plates were classified as successfully edited. In total, fifty colonies were tested. Sanger sequencing was then harnessed to confirm the deletion of the two genes. The sequencing files are provided in Supplementary Data 6. The desired deletion of the target genes was detected in 54% of fifty randomly selected colonies. Around 6% of the selected colonies showed a single deletion (2% for *araA* and 4% for *scrK*). Around 16% of the selected colonies showed deletion of two genes but not the third one (8%, 6%, and 2% showed deletion of *araA & scrK, araA & dhak, and scrK & dhak*, respectively).

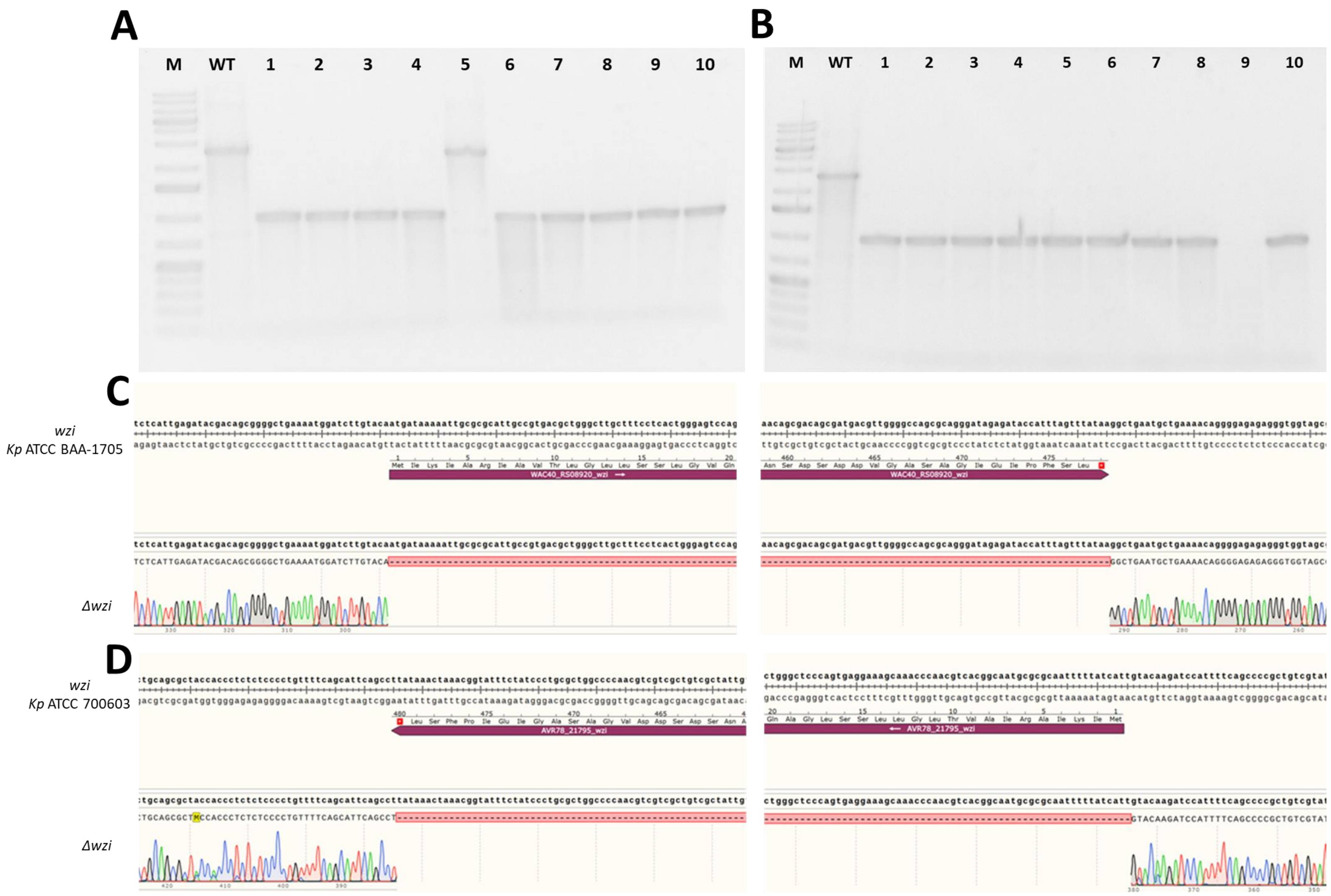

Supplementary Figure 18. Evaluation of *wzi* deletion in MDR *Kp* strains, *Kp* ATCC BAA-1705 **(A)** and *Kp* ATCC 700603 **(B)**. Colony PCR results of demonstrating the deletion of the *wzi* gene using primers that bind approximately 500 bp outside the deleted region. Lane M represents the marker; WT corresponds to the wild-type strain, which serves as a positive control for the unmodified *wzi* locus. **C) & D)** Evaluation of *wzi* deletion in *Kp* ATCC BAA-1705 and ATCC 700603 by Sanger sequencing. Screenshots showing alignment of sequencing files with the Kp genome sequence by SnapGene® 5.0.8. The sequencing files are provided in Supplementary Data 6.

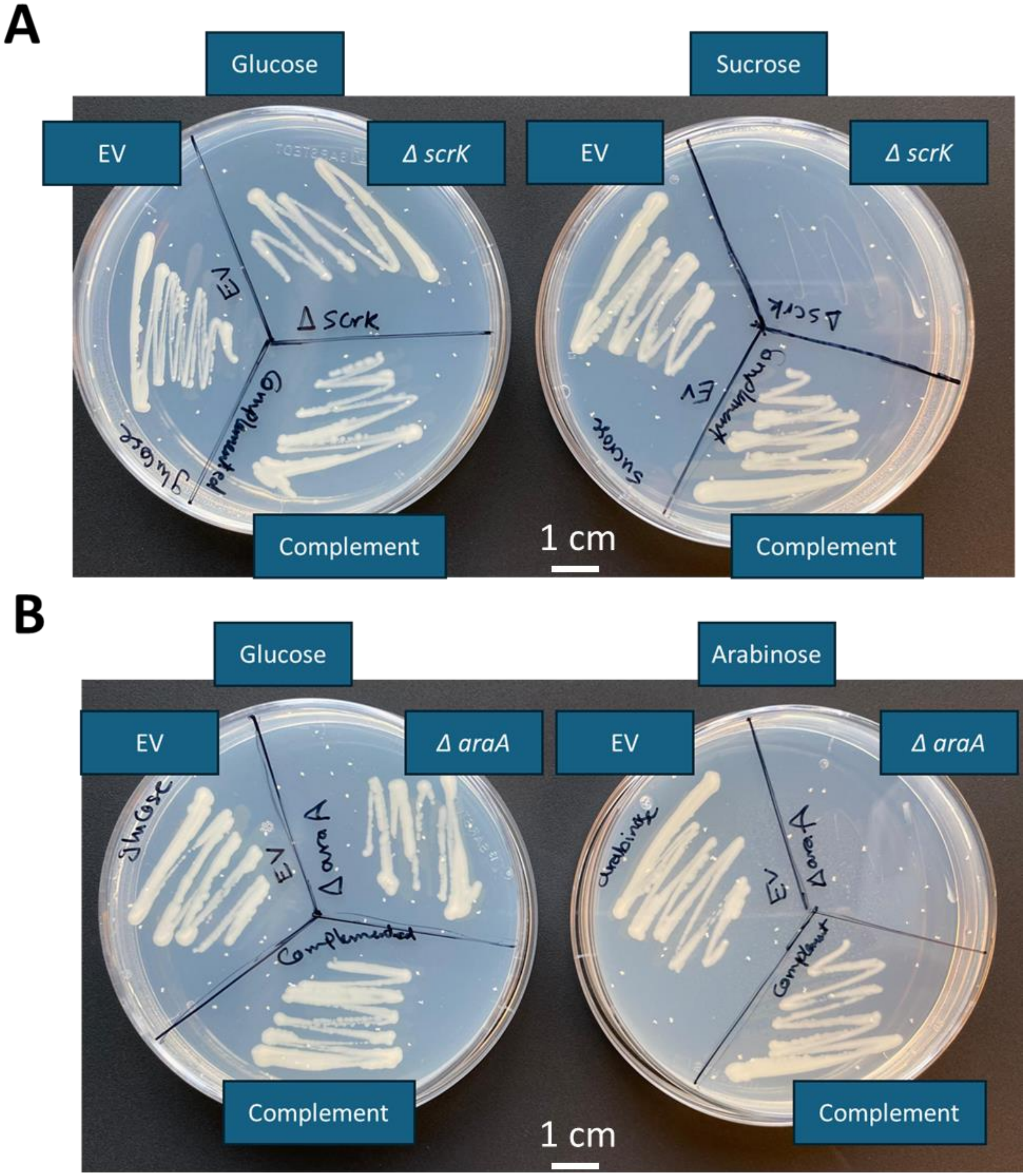

Supplementary Figure 19. Phenotypic complementation of RECKLEEN-edited mutants (Deletions) restores growth on selective carbon sources. (A) Δ*scrK and* (B) Δ*araA* mutants were complemented with plasmids expressing the corresponding wild-type genes. Restored growth on sucrose (for Δ*scrK*) and arabinose (for Δ*araA*) as sole carbon sources confirms that the observed phenotypes result from on-target gene disruption. Two biological replicates were performed for each complementation experiment. All relevant experimental data are available in Supplementary Data 4.

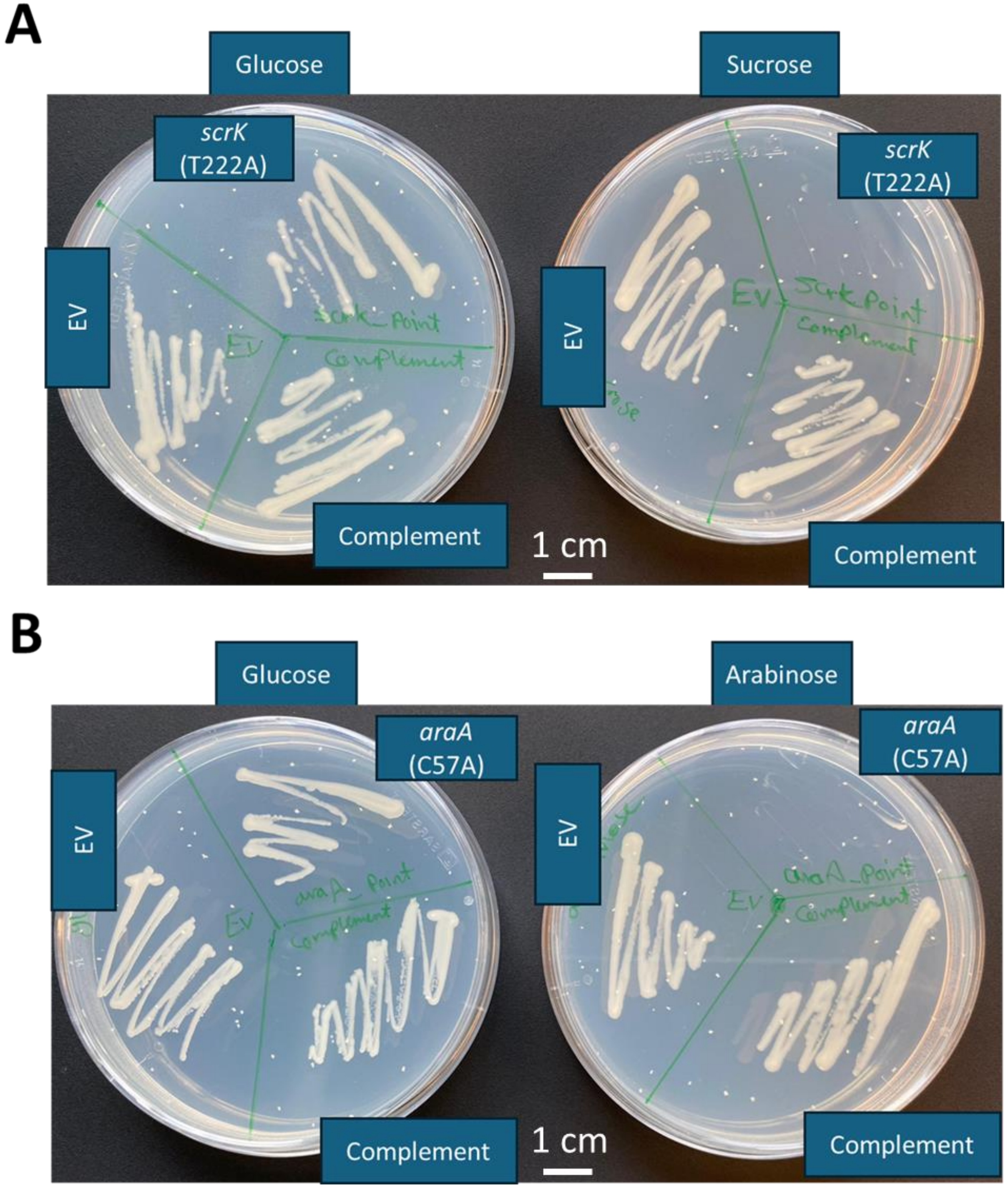

Supplementary Figure 20. Phenotypic complementation of RECKLEEN-edited mutants (point mutations) restores growth on selective carbon sources. (A) *scrK*(T222A) and **(B)** *araA*(C57A) mutants were complemented with plasmids expressing the corresponding wild-type genes. Restored growth on sucrose (for *scrK*(T222A)) and arabinose (for *araA(*C57A) as sole carbon sources confirms that the observed phenotypes result from on-target gene disruption. Two biological replicates were performed for each complementation experiment. All relevant experimental data are available in Supplementary Data 4.

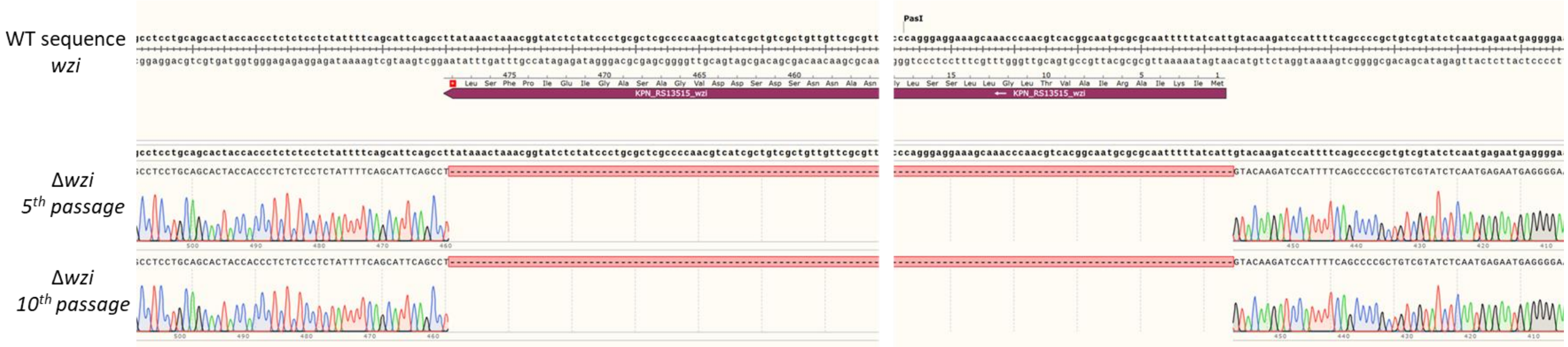

Supplementary Figure 21. Evaluation of the genetic stability of RECKLEEN-derived genome edits (deletions). Genomic DNA was extracted from a gene deletion mutant (Δ*wzi*), at passages five and ten. To confirm retention of the engineered edit, locus-specific primers flanking the target site were used to amplify the region by PCR. Amplicons were Sanger sequenced by Microsynth Seqlab, using PCR fragments and primers binding upstream or downstream of the targeted modification. The alignment of sequencing files was conducted using SnapGene® 5.0.8 software with screenshots provided. The WT sequence is shown alongside the sequences of the 5th and 10th passages to illustrate the dominance of the engineered edit in the population. All relevant sequencing files are available in Supplementary Data 6.

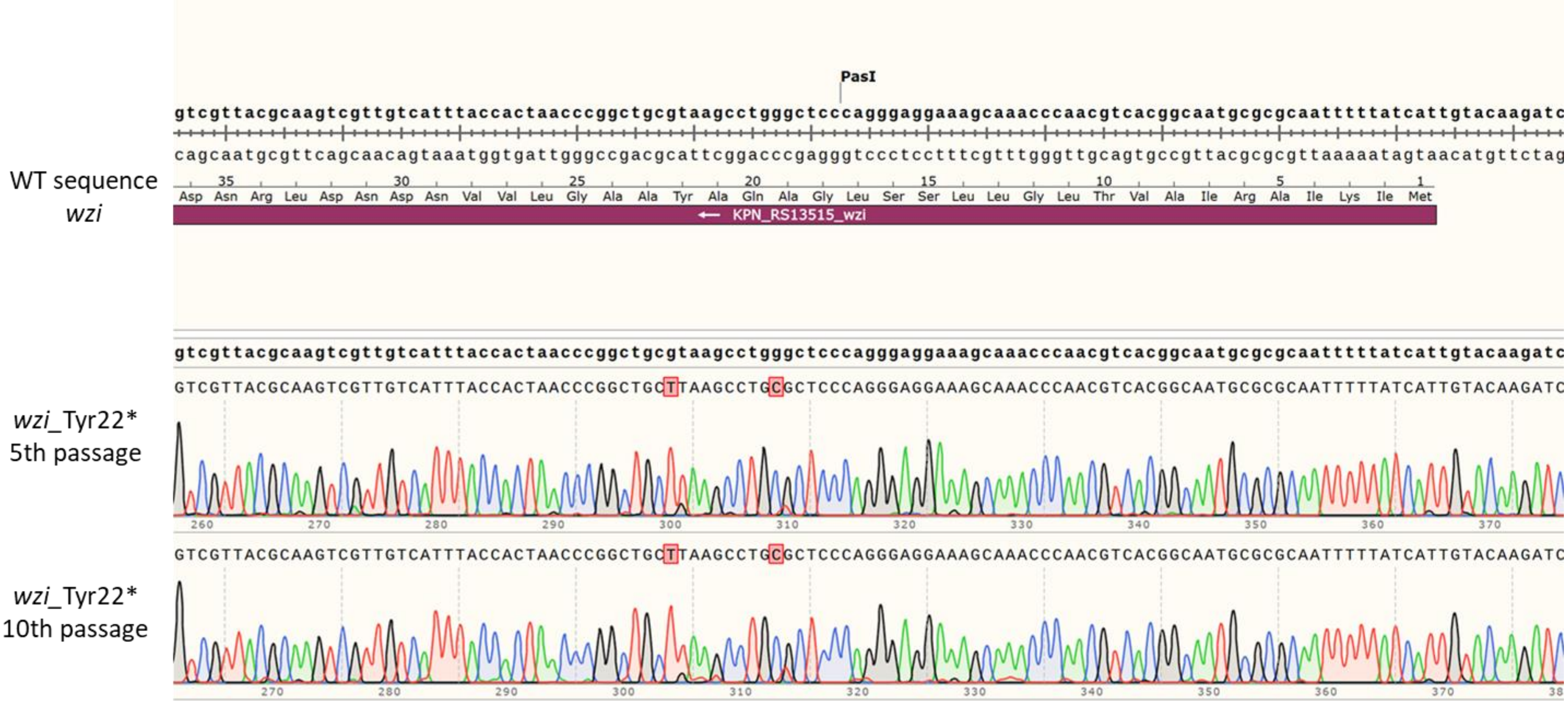

Supplementary Figure 22. Evaluation of the genetic stability of RECKLEEN-derived genome edits (Point mutations). Genomic DNA was extracted from a point mutation strain *(wzi C66A)*, at passages five and ten. To confirm retention of the engineered edit, locus-specific primers flanking the target site were used to amplify the region by PCR. Amplicons were Sanger sequenced by Microsynth Seqlab, using PCR fragments and primers binding upstream or downstream of the targeted modification. The alignment of sequencing files was conducted using SnapGene® 5.0.8 software with screenshots provided. The WT sequence is shown alongside the sequences of the 5th and 10th passages to illustrate the retention of the engineered edit. All relevant sequencing files are available in Supplementary Data 6.

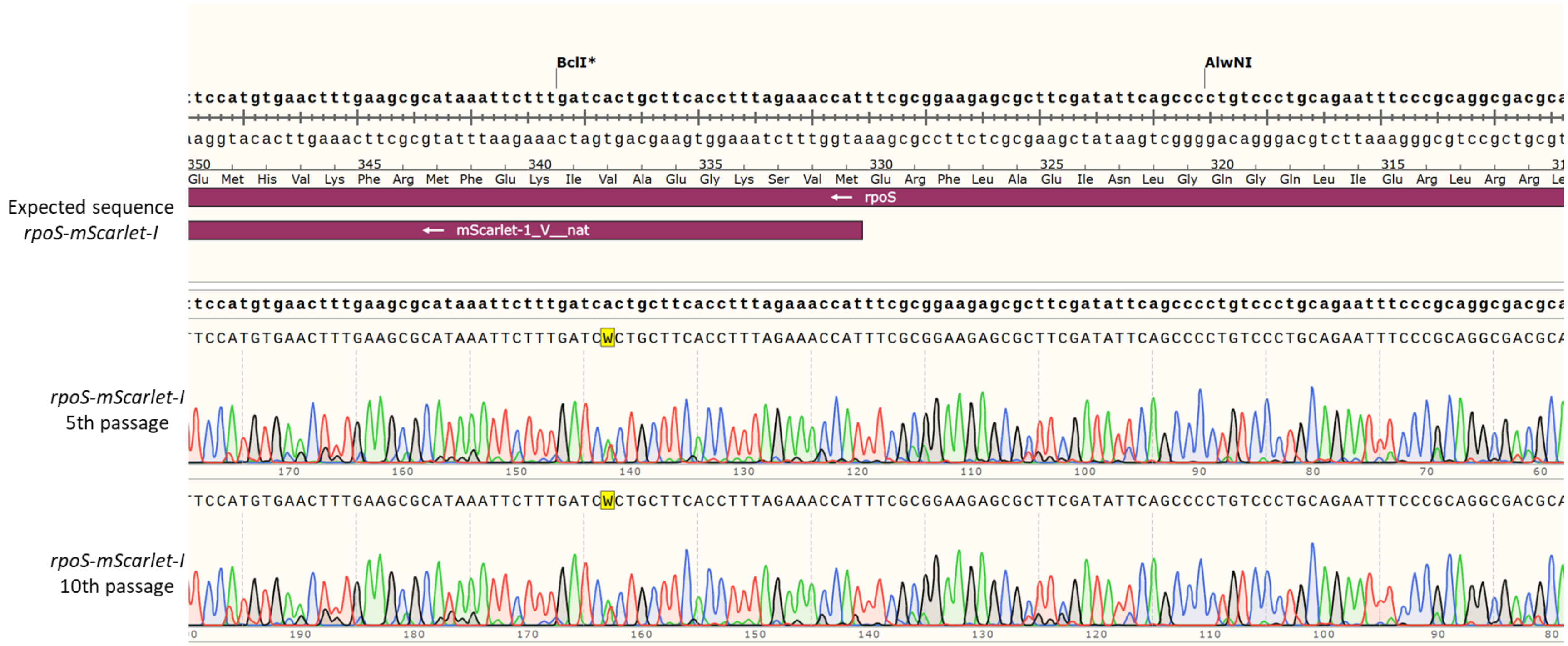

Supplementary Figure 23. Evaluation of the genetic stability of RECKLEEN-derived genome edits (DNA integration). Genomic DNA was extracted from a DNA integration strain (*rpoS-mScarletI*), at passages five and ten. To confirm retention of the engineered edit, locus-specific primers flanking the target site were used to amplify the region by PCR. Amplicons were Sanger sequenced by Microsynth Seqlab, using PCR fragments and primers binding upstream or downstream of the targeted modification. The alignment of sequencing files was conducted using SnapGene® 5.0.8 software with screenshots provided. The *rpoS-mScarletI* expected sequence is shown alongside the sequences of the 5th and 10th passages to illustrate the retention of the engineered edit. All relevant sequencing files are available in Supplementary Data 6.

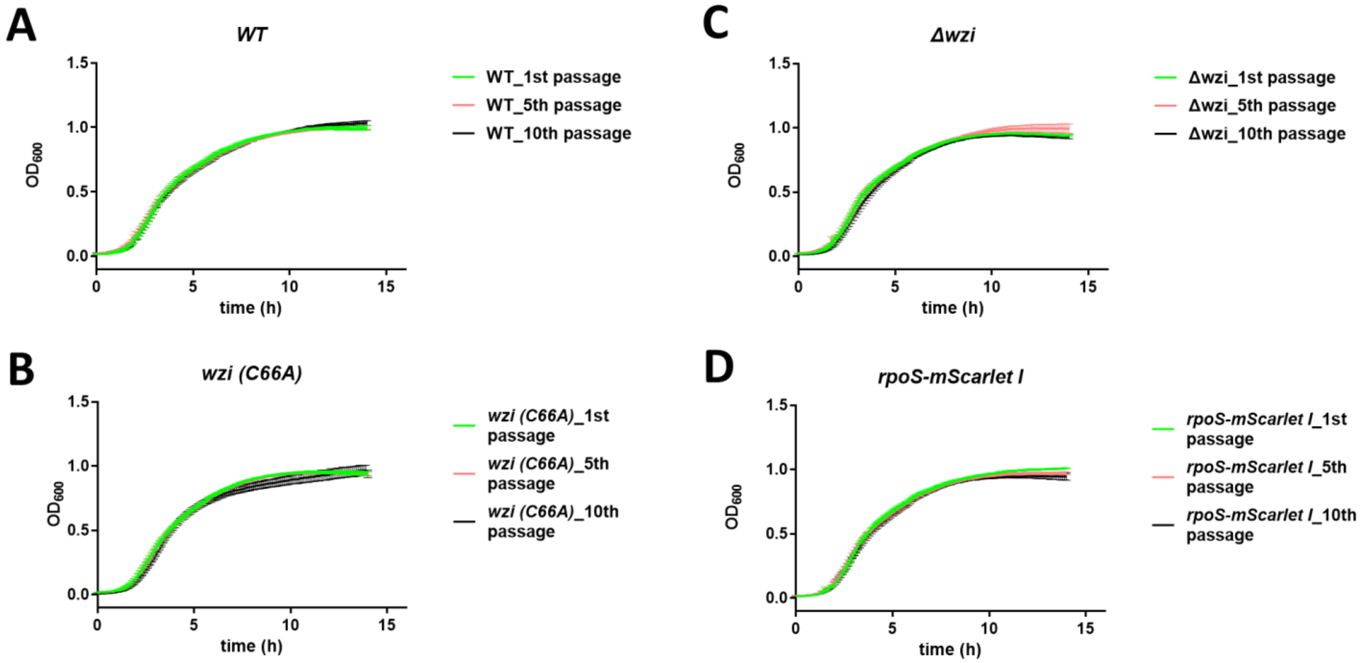

Supplementary Figure 24. Evaluation of long-term fitness of RECKLEEN-derived genome edits. Growth curves for **(A)** wildtype (WT) *Kp*, **(B)** a point mutation strain (*wzi* C66A)**, (C)** a gene deletion mutant (Δ*wzi*), and **(D)** a chromosomal insertion strain *(rpoS-mScarletI).* Compared to the wild-type strain, the growth kinetics of all mutants remained unchanged, showing no significant difference in doubling times or overall growth profiles. Data represents the mean of three replicates, and the error bars represent the standard deviation from the mean.

Supplementary Tables provided in Supplementary Data 2

Supplementary Table 1: Assembly of plasmids used in this study.

Supplementary Table 2: Bacterial strains used in this study.

Supplementary Table 3: Oligonucleotides used to assemble *sgRNA* sequences.

Supplementary Table 4: Oligonucleotides used for the construction of dDNA template plasmids.

Supplementary Table 5: Oligonucleotides to generate dDNA fragments from dDNA template plasmids.

Supplementary Table 6: ssDNA oligonucleotides to generate dDNA fragments.

Supplementary Table 7: Whole-genome sequencing analysis of Δ*wzi*.

Supplementary Table 8: Oligonucleotides to generate *scrK* and *araA* fragments from *Kp* for complementation experiments.
