# Supplementary material for: RECKLEEN is a lambda Red/CRISPR-Cas9 based single plasmid platform for enhanced genome editing in Klebsiella pneumoniae
Source: Commun Biol. 2025 Oct 30;8:1509. doi: 10.1038/s42003-025-08934-8 (PMC12575629; doi:10.1038/s42003-025-08934-8)
Supplement: Supplementary file 2 — Description of additional supplementary files [file 42003_2025_8934_MOESM2_ESM.pdf]

Description of Additional Supplementary Files

File name: Supplementary Data 1

Description: Plasmid maps of all RECKLEEN plasmids

File name: Supplementary Data 2

Description: Supplementary tables 1 to 8 (Details of supplementary tables are provided in Supplementary information).

File name: Supplementary Data 3

Description: Gels of colony PCRs used to check *wzi* deletions

File name: Supplementary Data 4

Description: Growth on M9 minimal medium agar plates for phenotypic characterization of genome-edited mutants

File name: Supplementary Data 5

Description: The source data behind the graphs in the paper

File name: Supplementary Data 6

Description: Sanger Sequencing data performed by Microsynth

File name: Supplementary Data 7

Description: Raw data used to generate growth curves in the paper
