# Supplementary figures and images for: RECKLEEN is a lambda Red/CRISPR-Cas9 based single plasmid platform for enhanced genome editing in Klebsiella pneumoniae
Source: Commun Biol. 2025 Oct 30;8:1509. doi: 10.1038/s42003-025-08934-8 (PMC12575629; doi:10.1038/s42003-025-08934-8)

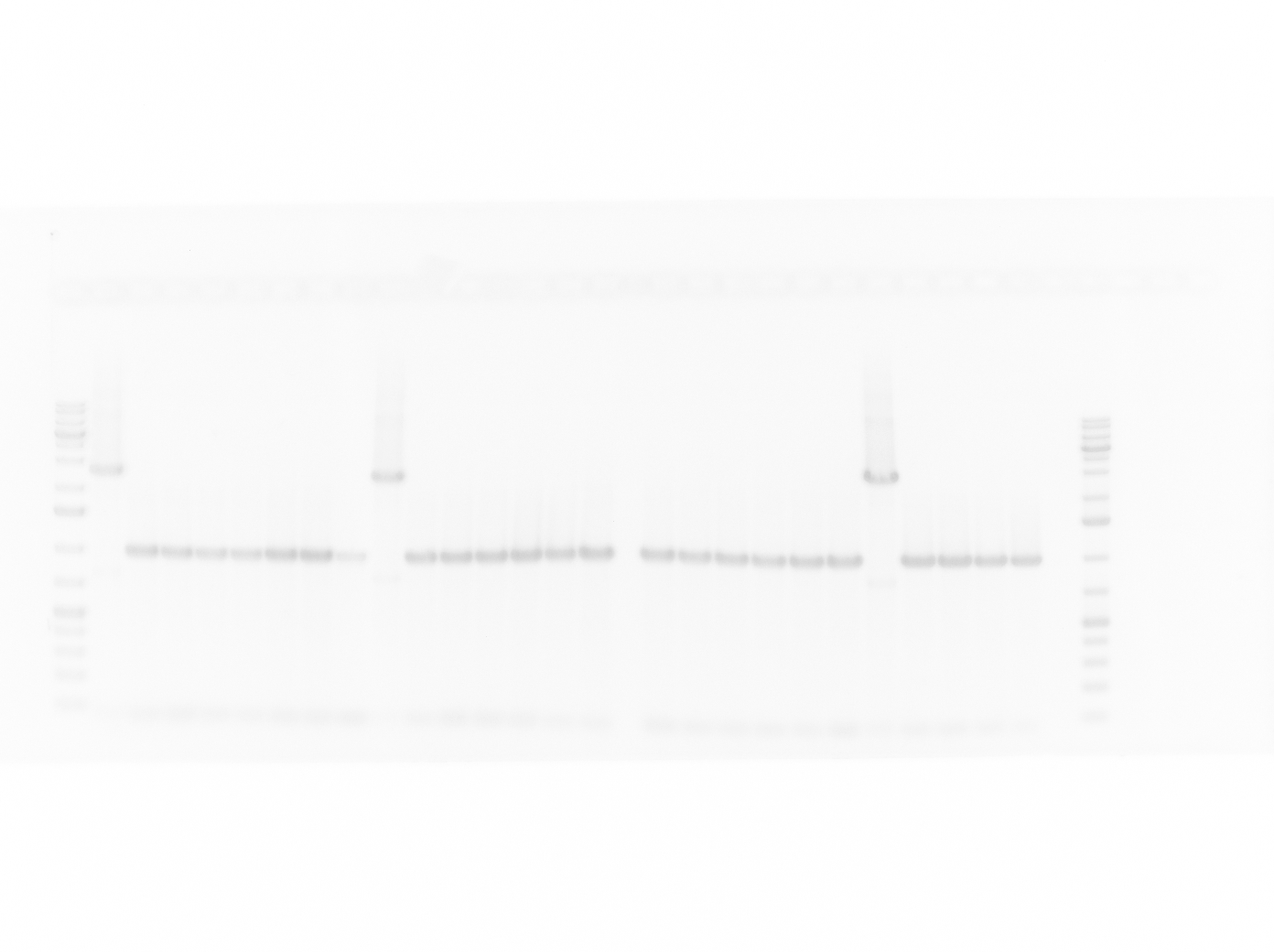

Supplement: Supplementary file 5 — Supplementary Data 3 [file 42003_2025_8934_MOESM5_ESM.zip › Supplementary Data 3/Kp_700721/colonypcrwzi_rep1.bmp]

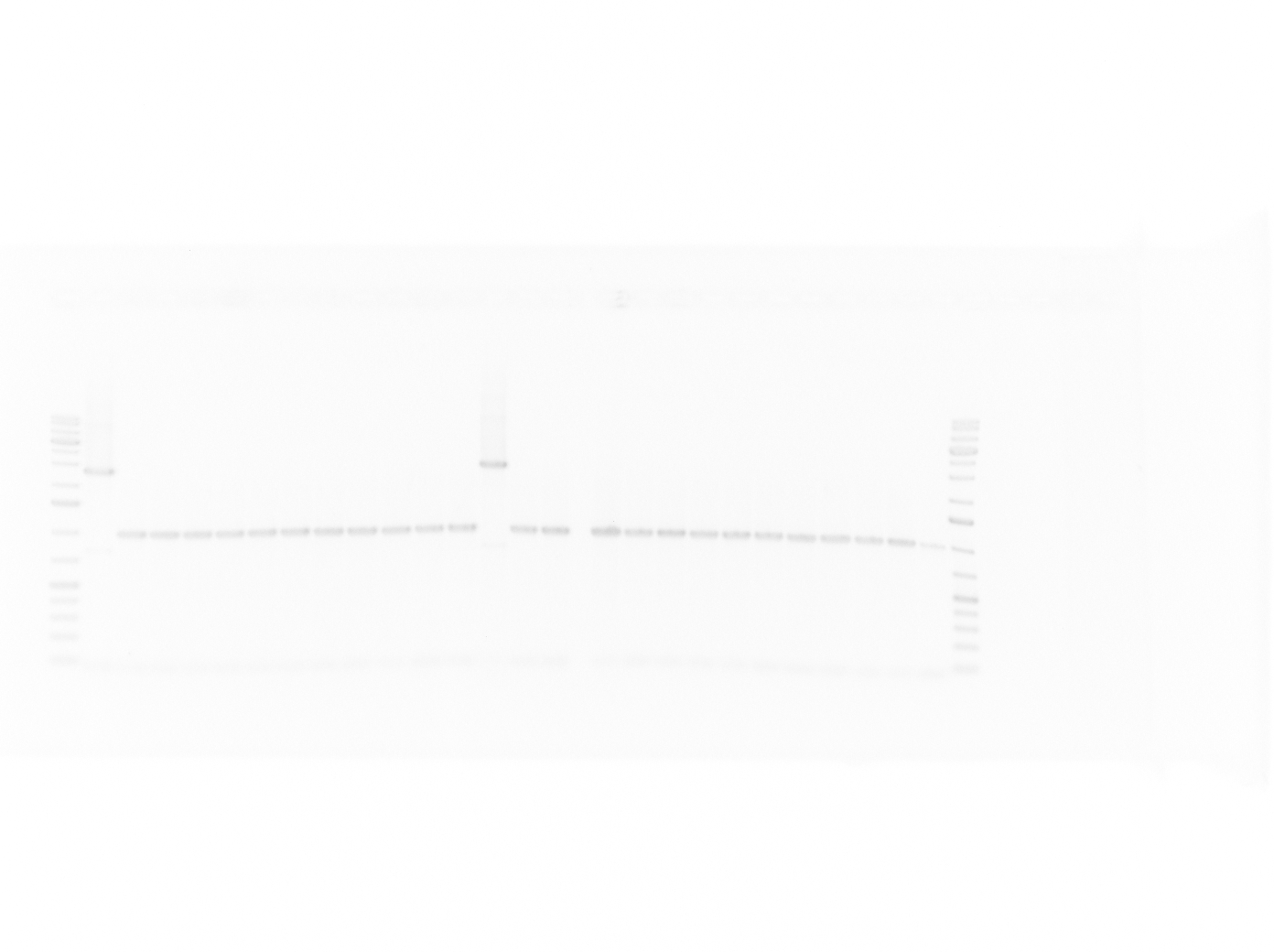

Supplement: Supplementary file 5 — Supplementary Data 3 [file 42003_2025_8934_MOESM5_ESM.zip › Supplementary Data 3/Kp_700721/colonypcrwzi_rep2.bmp]

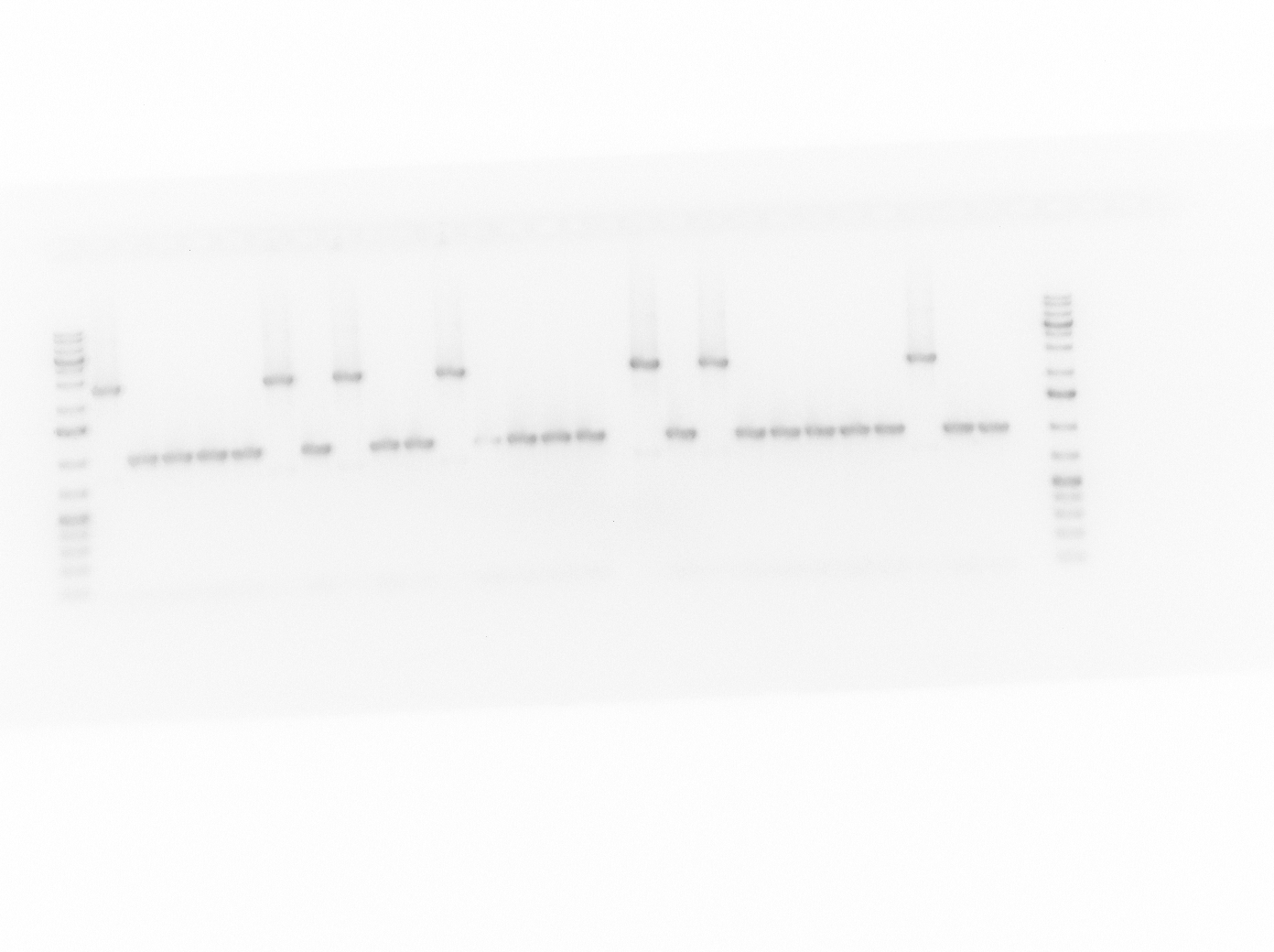

Supplement: Supplementary file 5 — Supplementary Data 3 [file 42003_2025_8934_MOESM5_ESM.zip › Supplementary Data 3/Kp_700721/colonypcrwzi_rep3.bmp]

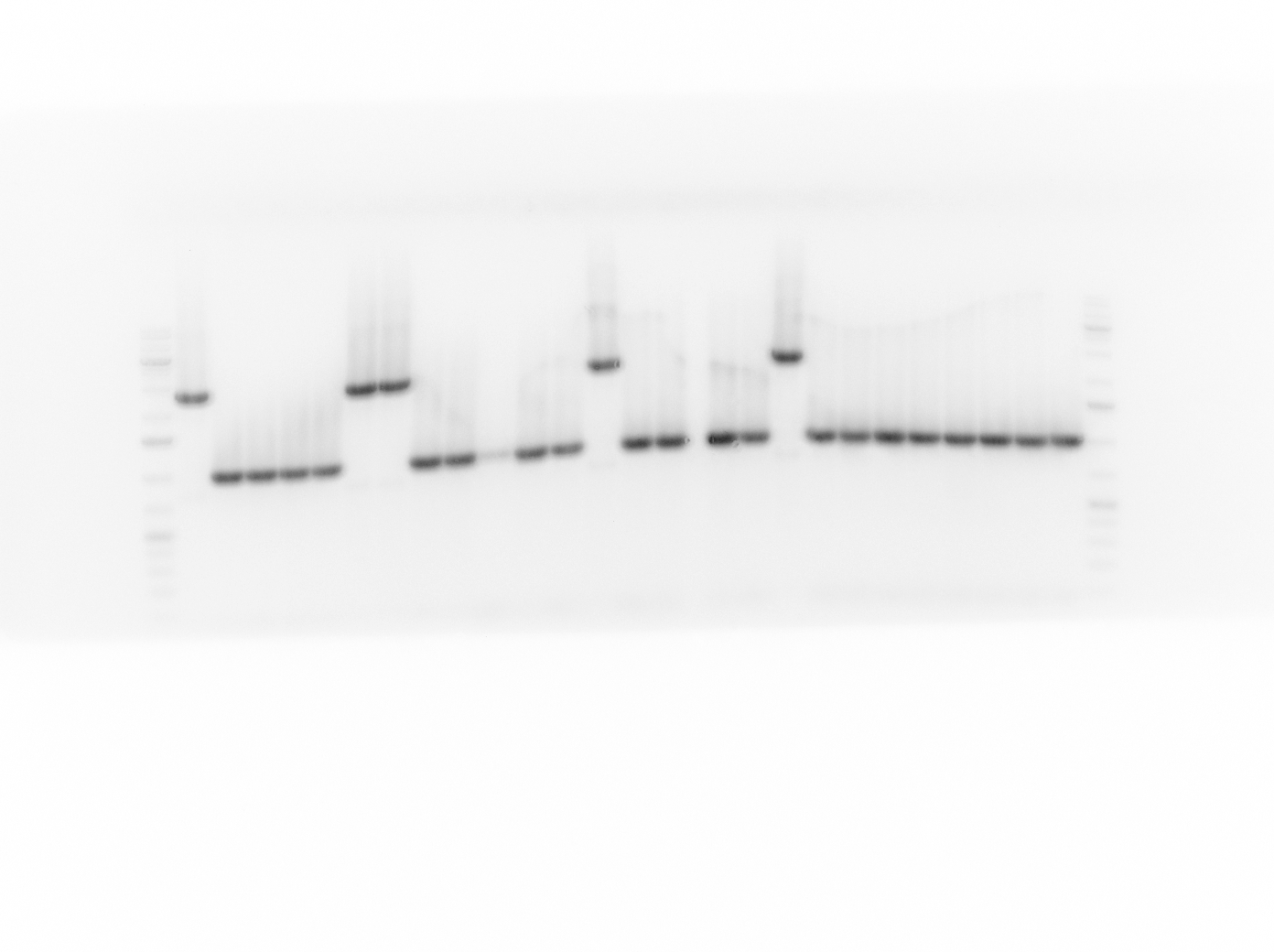

Supplement: Supplementary file 5 — Supplementary Data 3 [file 42003_2025_8934_MOESM5_ESM.zip › Supplementary Data 3/Kp_700721/colonypcrwzi_rep4.bmp]

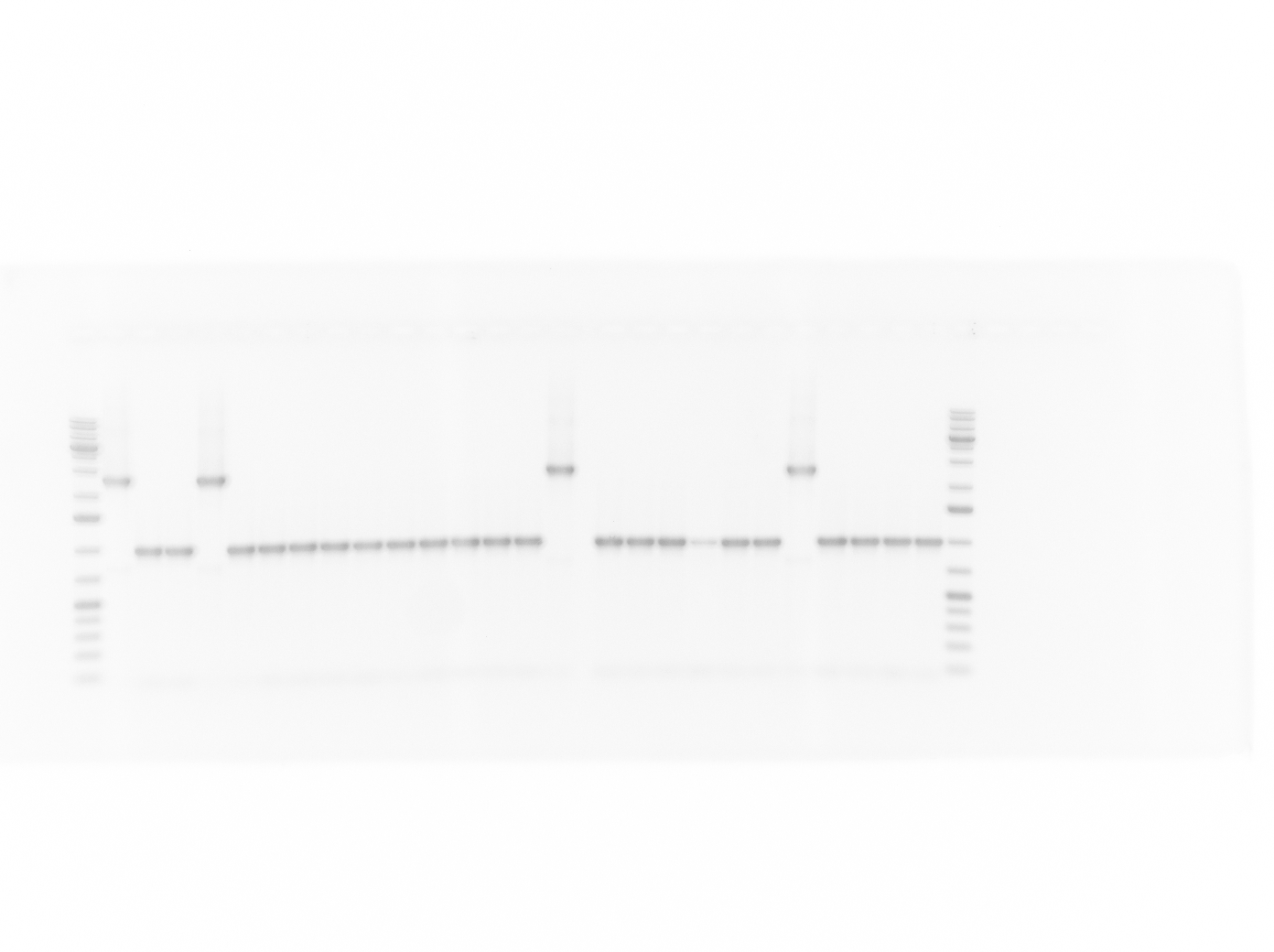

Supplement: Supplementary file 5 — Supplementary Data 3 [file 42003_2025_8934_MOESM5_ESM.zip › Supplementary Data 3/Kp_700721/colonypcrwzi_rep5.bmp]

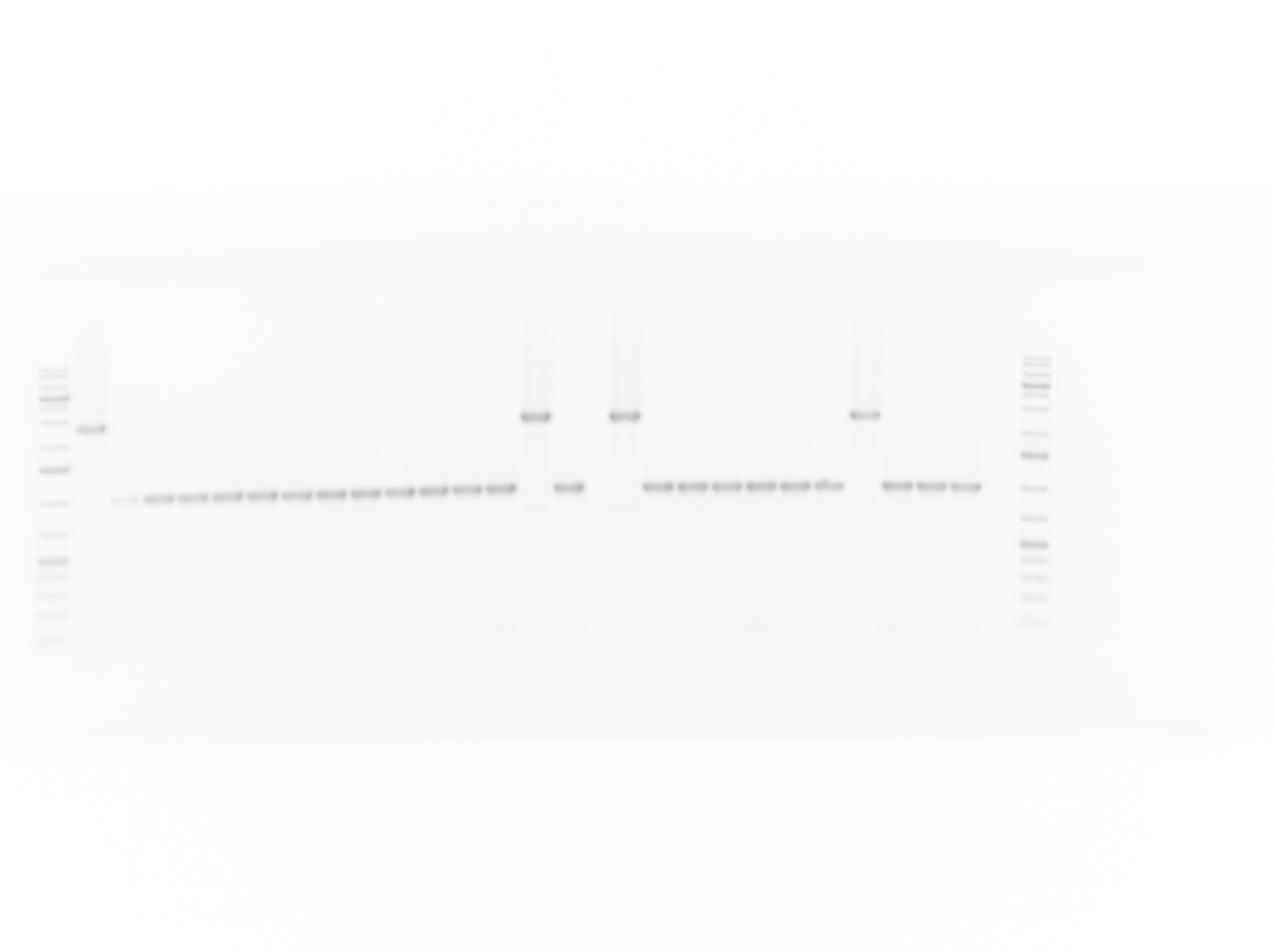

Supplement: Supplementary file 5 — Supplementary Data 3 [file 42003_2025_8934_MOESM5_ESM.zip › Supplementary Data 3/Kp_700721/colonypcrwzi_rep6.bmp]

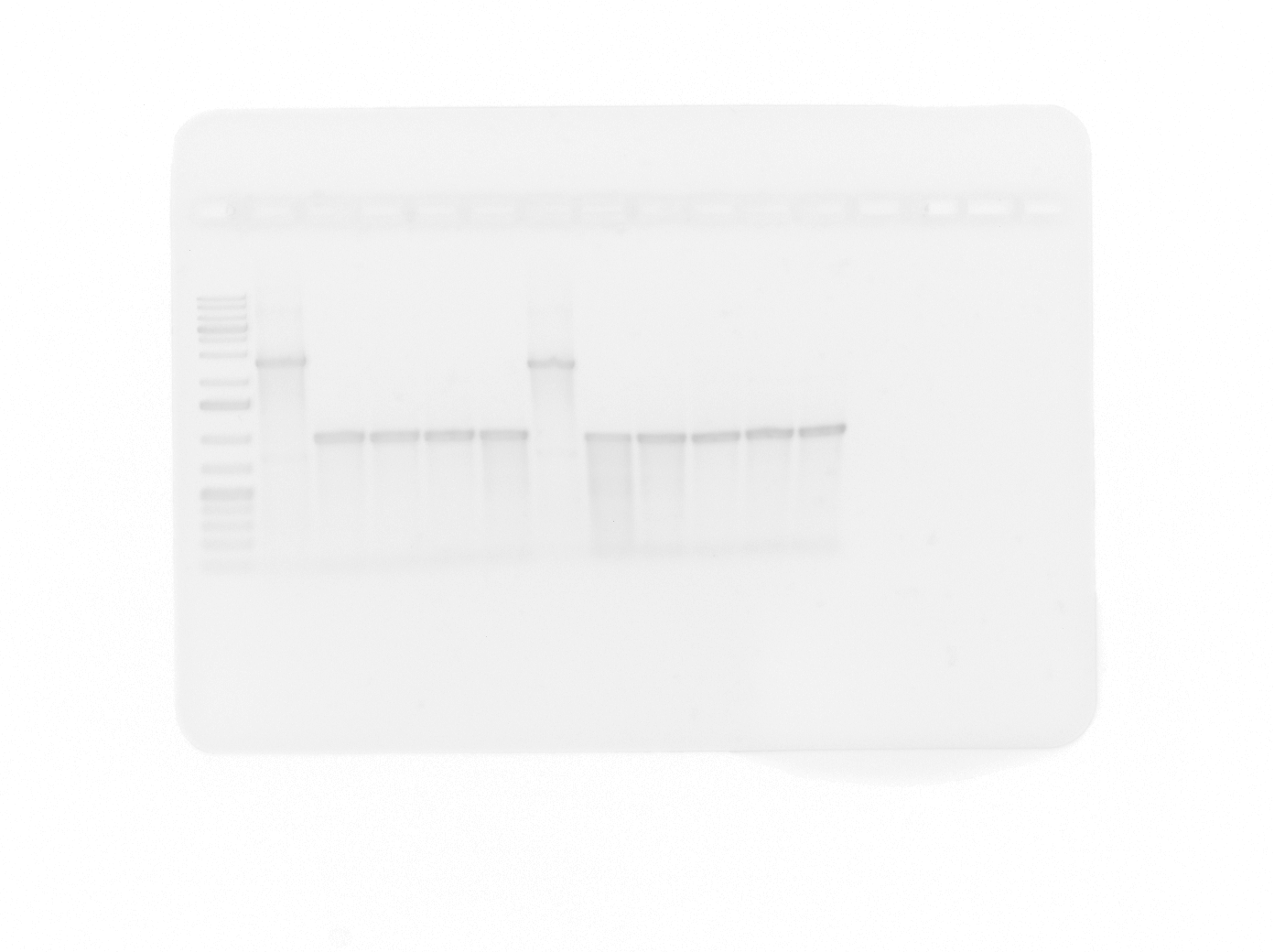

Supplement: Supplementary file 5 — Supplementary Data 3 [file 42003_2025_8934_MOESM5_ESM.zip › Supplementary Data 3/Kp_BAA1705/BAA1705_wzi_deletion colonypcr.bmp]

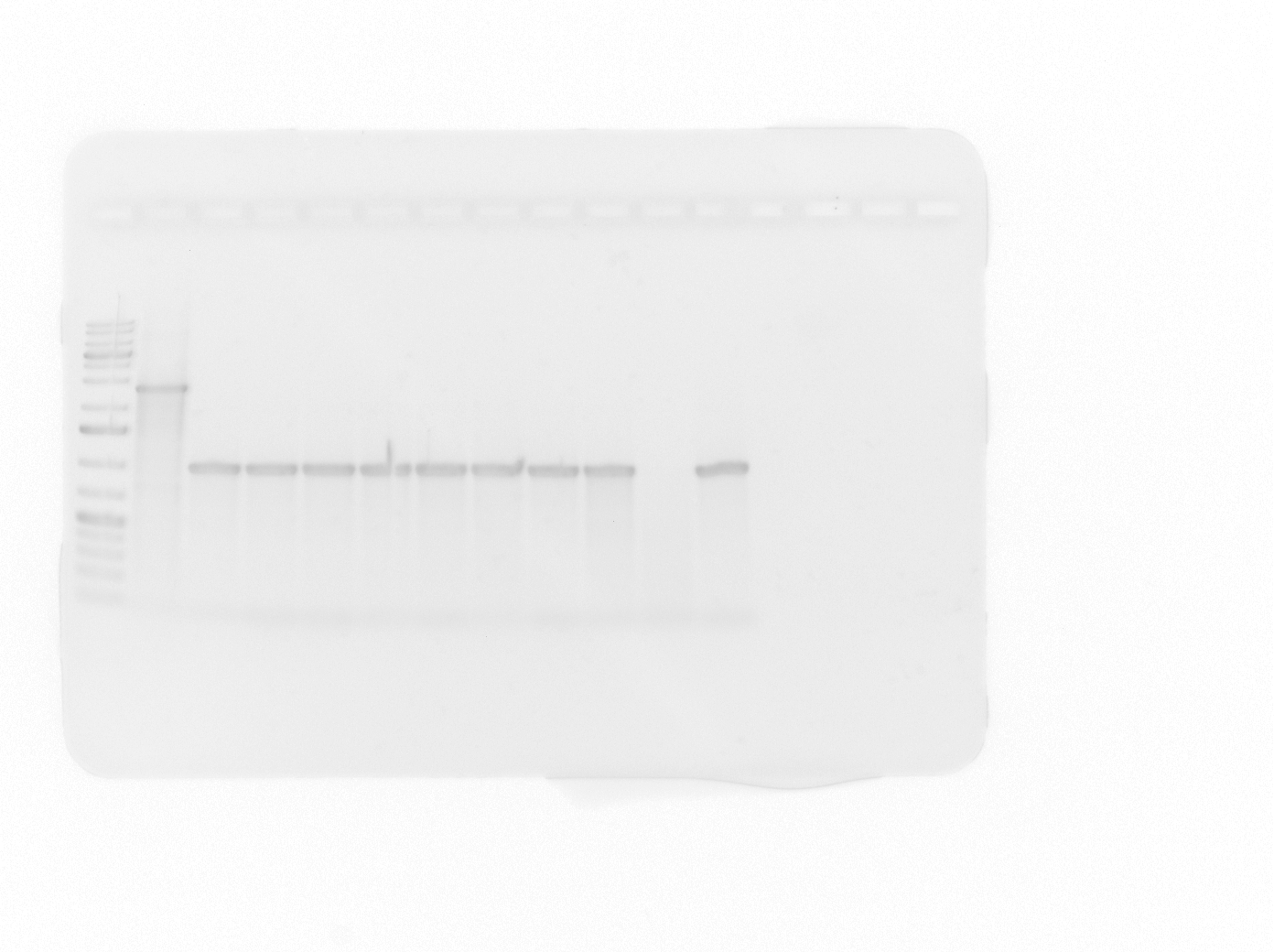

Supplement: Supplementary file 5 — Supplementary Data 3 [file 42003_2025_8934_MOESM5_ESM.zip › Supplementary Data 3/kp_700603/kp700603_wzi_deletion.bmp]

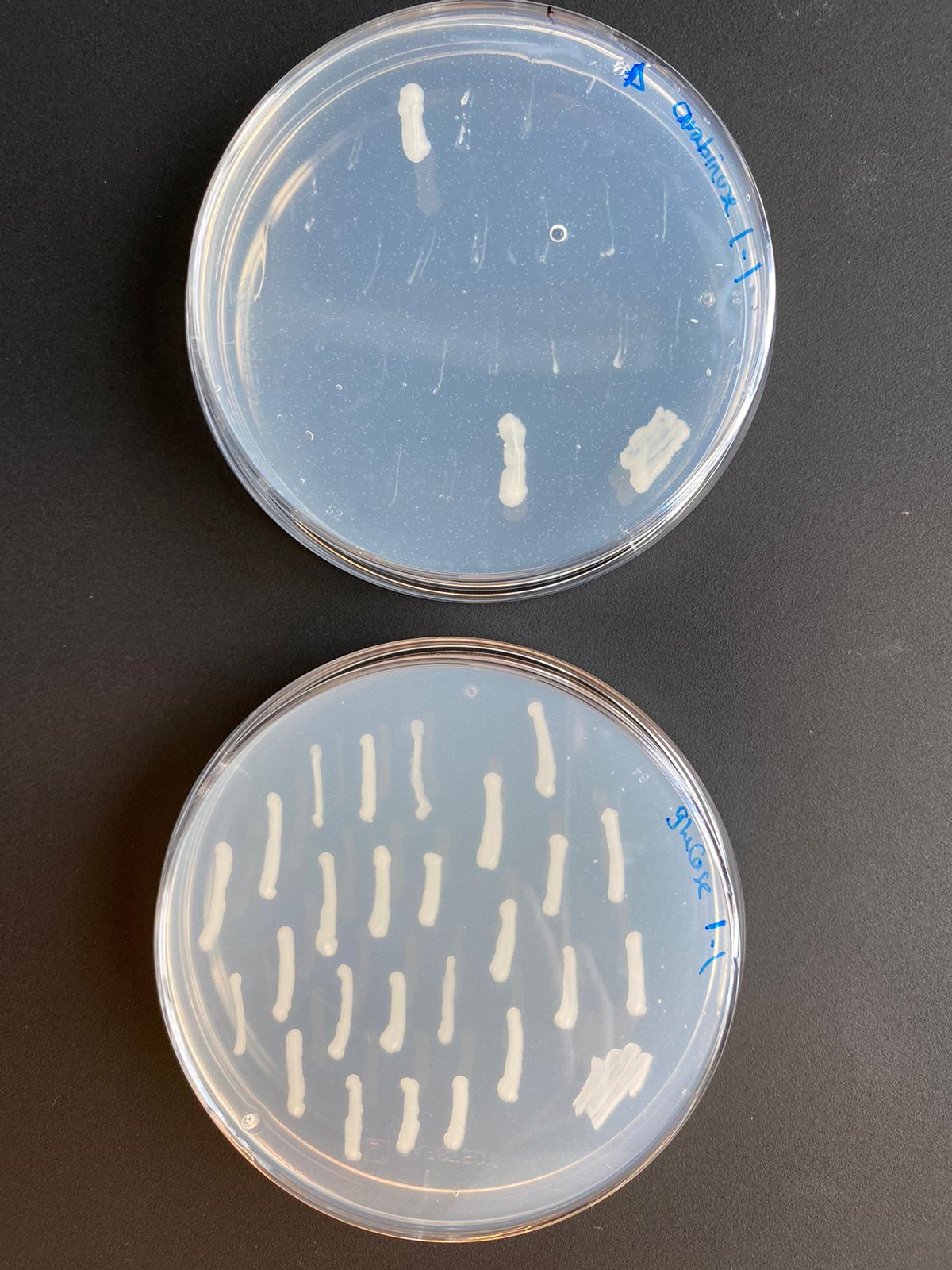

Supplement: Supplementary file 6 — Supplementary Data 4 [file 42003_2025_8934_MOESM6_ESM.zip › Supplementary Data 4/Deletion/araA_deletion/araA_deletion1.jpeg]

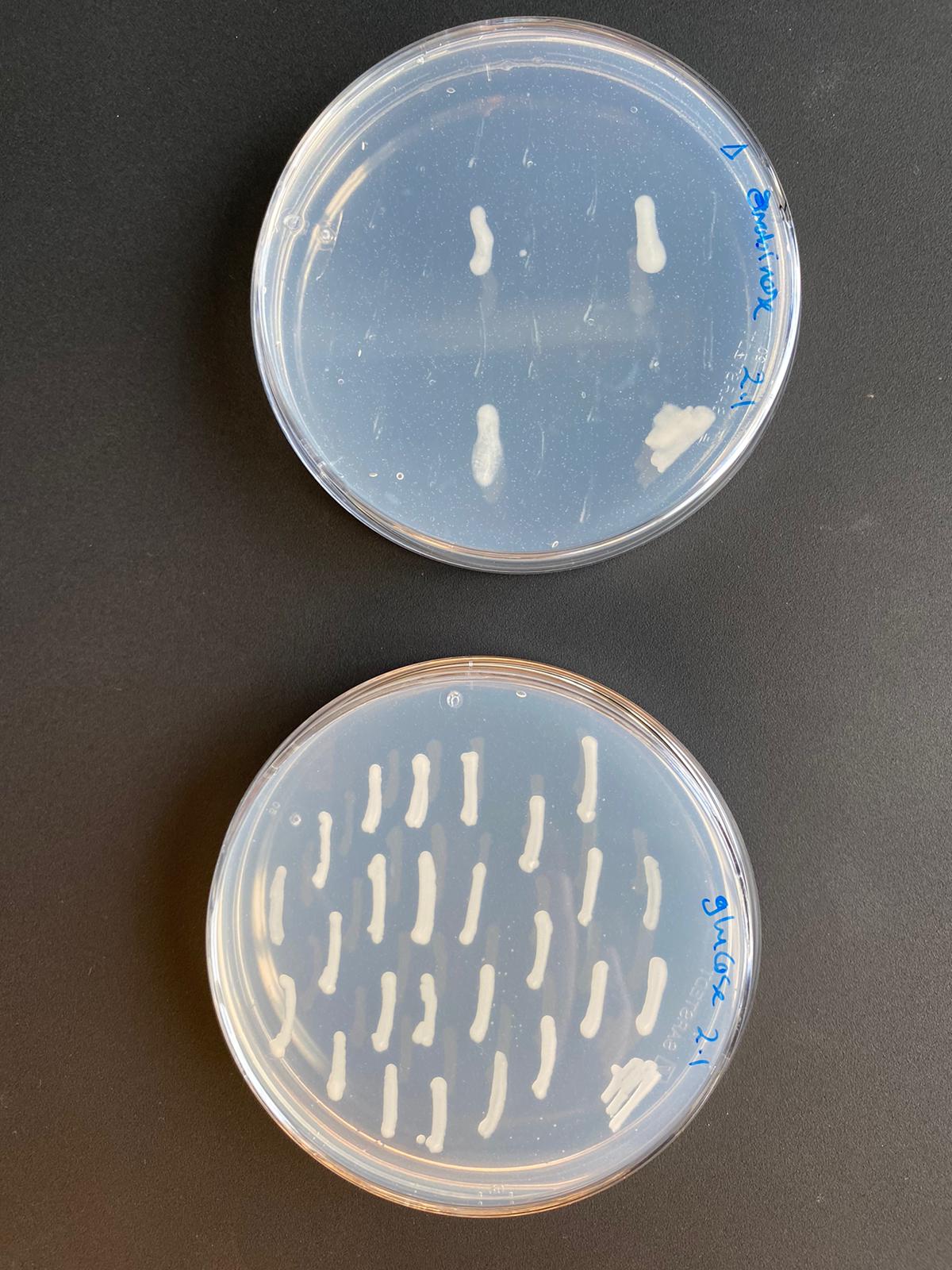

Supplement: Supplementary file 6 — Supplementary Data 4 [file 42003_2025_8934_MOESM6_ESM.zip › Supplementary Data 4/Deletion/araA_deletion/araA_deletion2.jpeg]

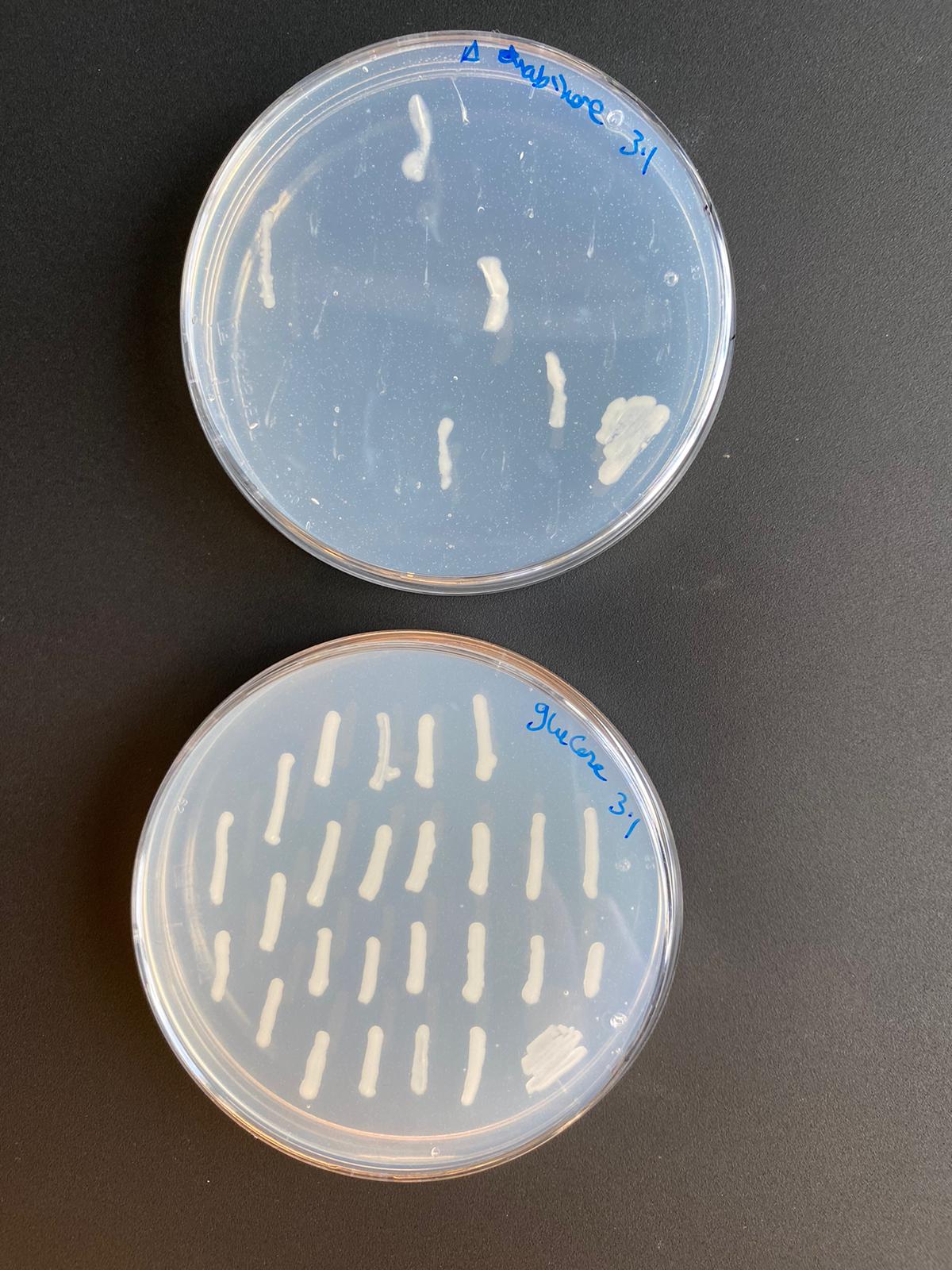

Supplement: Supplementary file 6 — Supplementary Data 4 [file 42003_2025_8934_MOESM6_ESM.zip › Supplementary Data 4/Deletion/araA_deletion/araA_deletion3.jpeg]

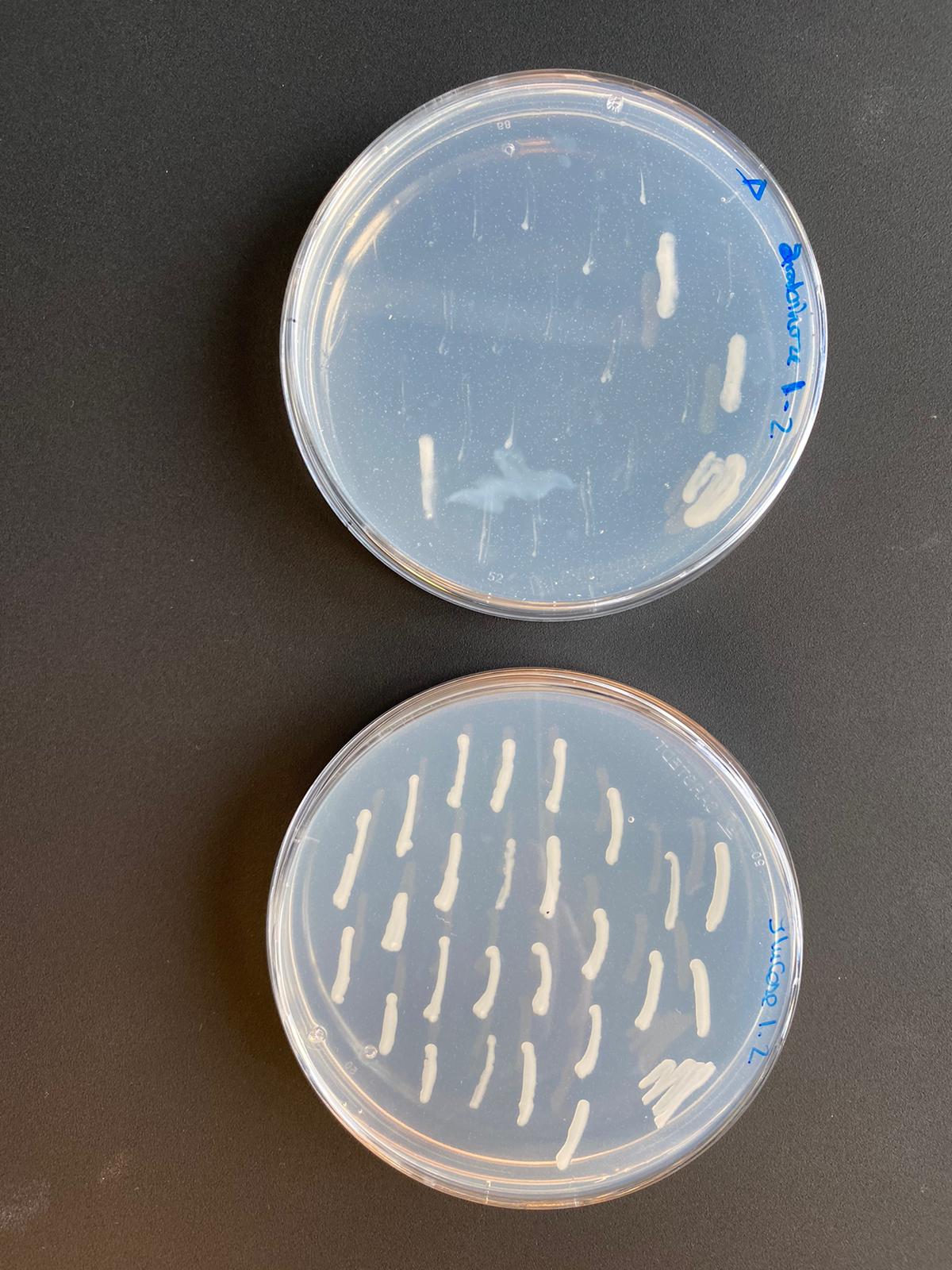

Supplement: Supplementary file 6 — Supplementary Data 4 [file 42003_2025_8934_MOESM6_ESM.zip › Supplementary Data 4/Deletion/araA_deletion/araA_deletion4.jpeg]

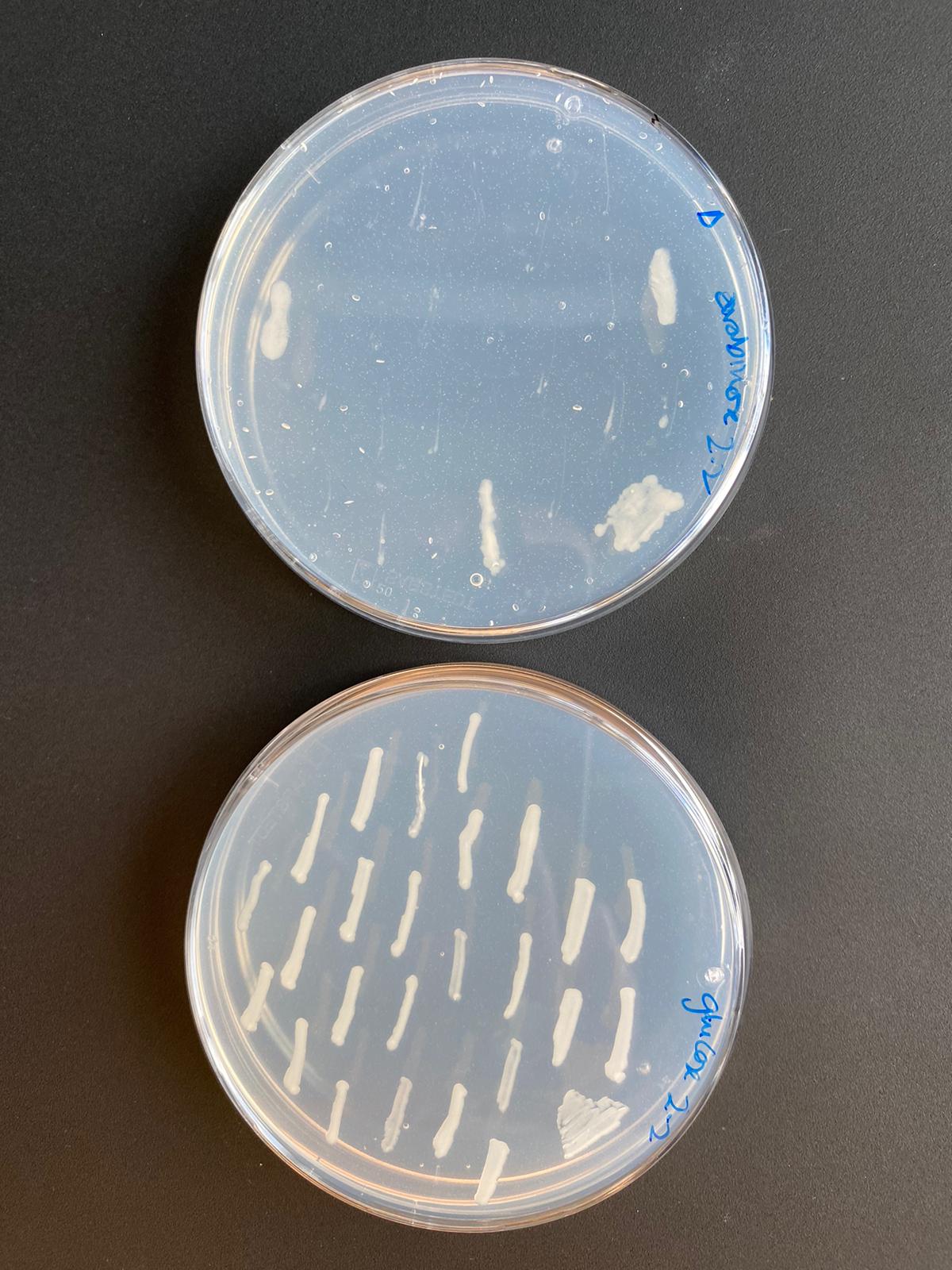

Supplement: Supplementary file 6 — Supplementary Data 4 [file 42003_2025_8934_MOESM6_ESM.zip › Supplementary Data 4/Deletion/araA_deletion/araA_deletion5.jpeg]

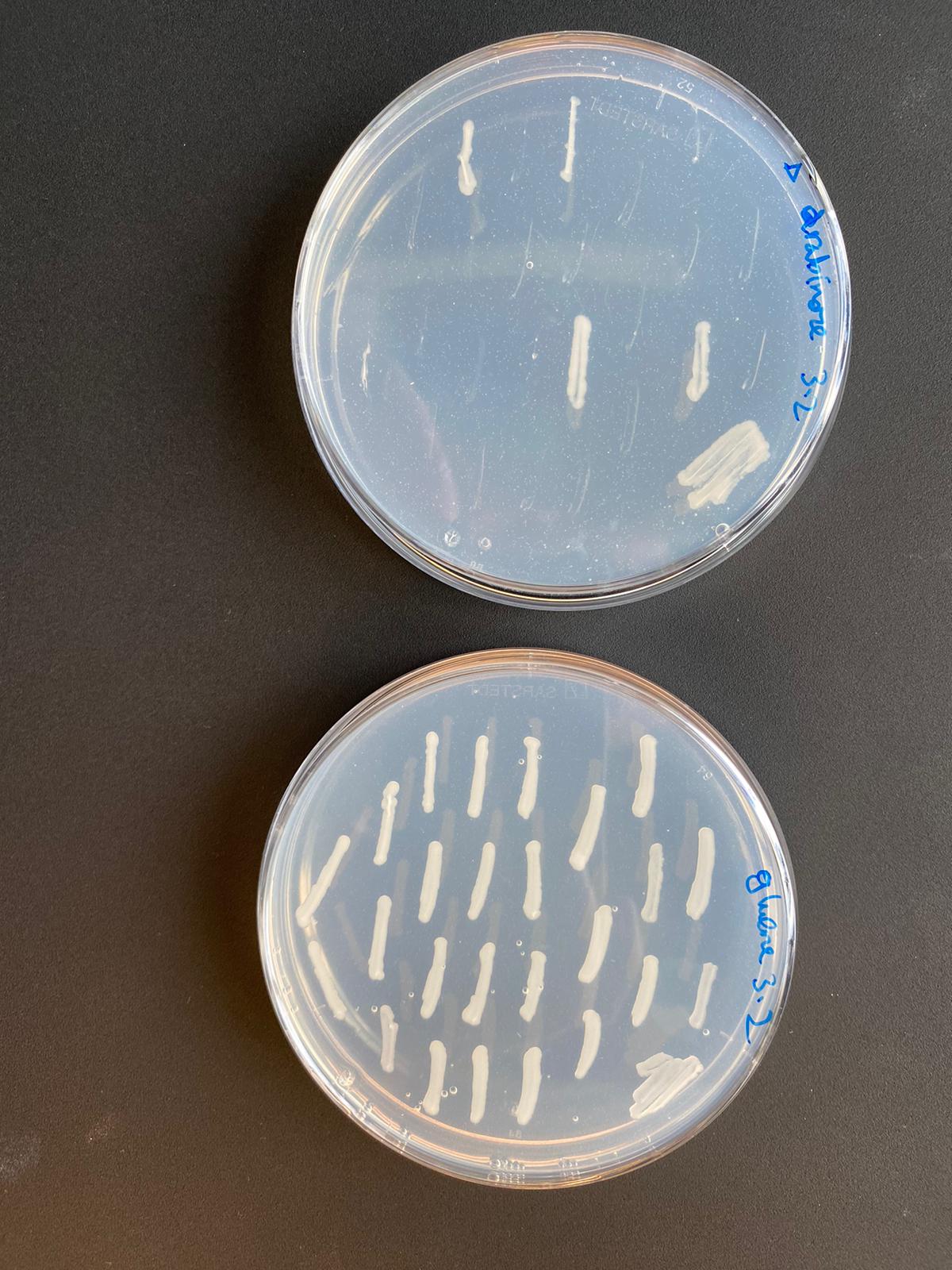

Supplement: Supplementary file 6 — Supplementary Data 4 [file 42003_2025_8934_MOESM6_ESM.zip › Supplementary Data 4/Deletion/araA_deletion/araA_deletion6.jpeg]

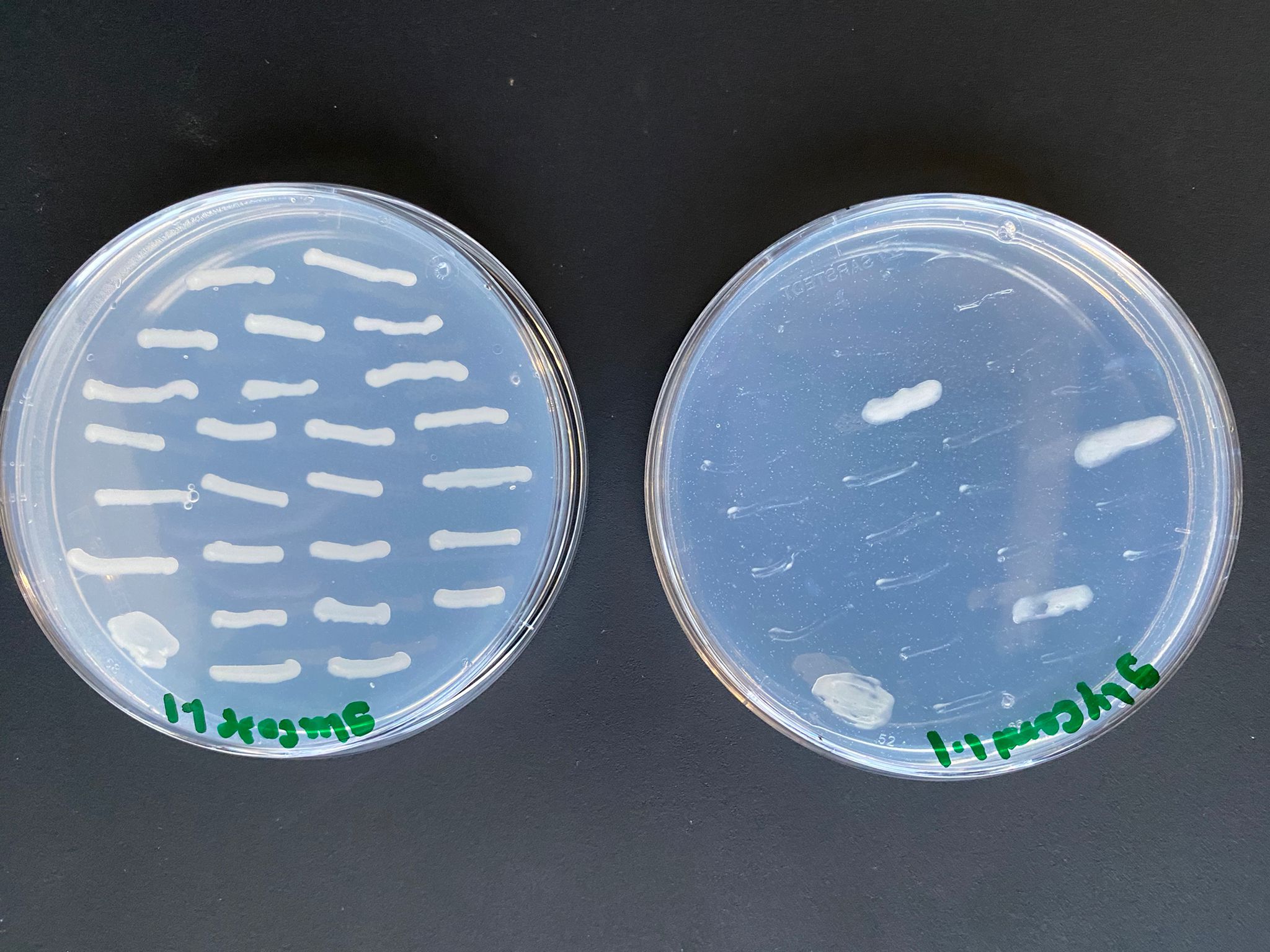

Supplement: Supplementary file 6 — Supplementary Data 4 [file 42003_2025_8934_MOESM6_ESM.zip › Supplementary Data 4/Deletion/dhaK_deletion/dhaK_deletion_1.jpeg]

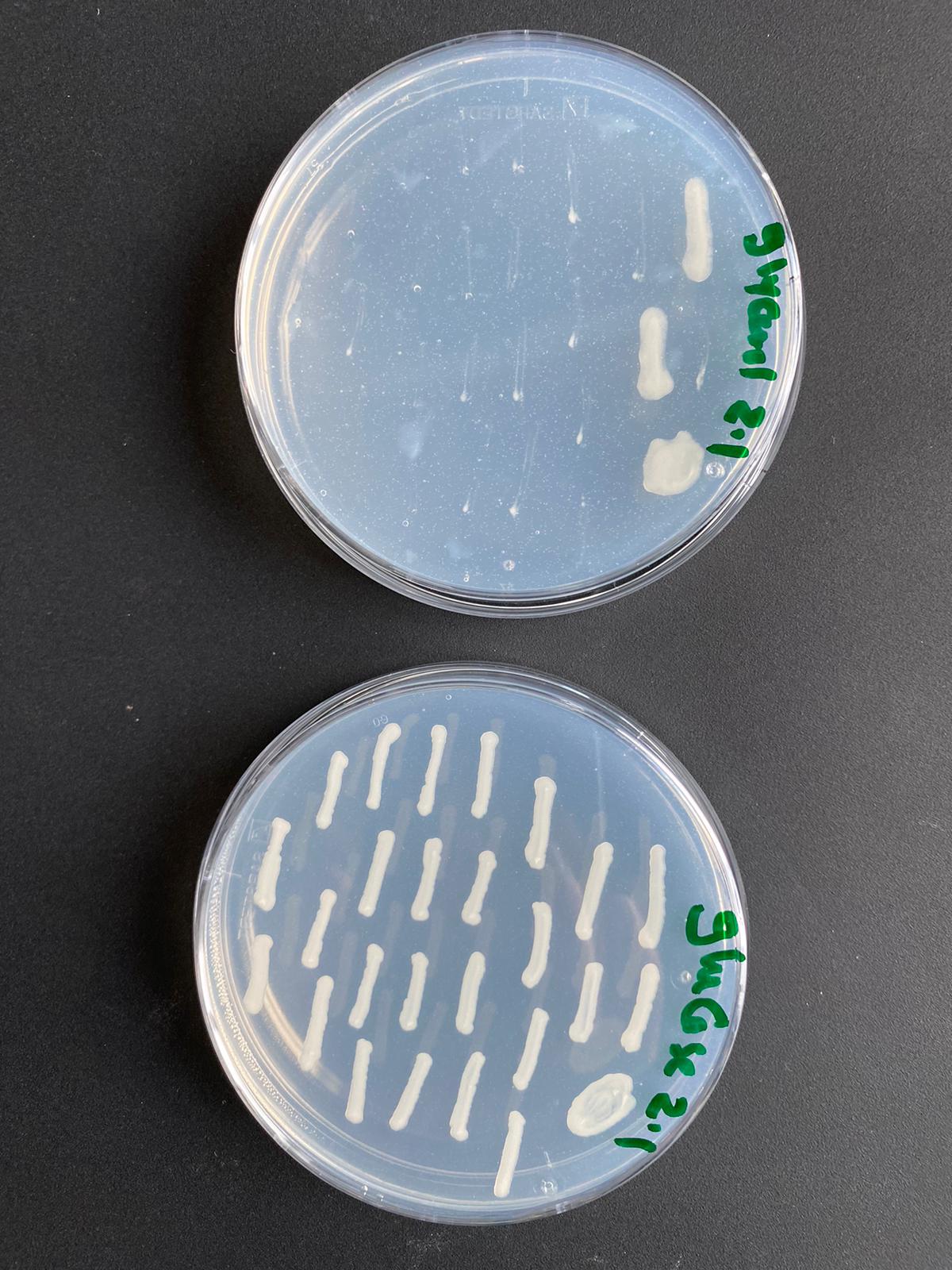

Supplement: Supplementary file 6 — Supplementary Data 4 [file 42003_2025_8934_MOESM6_ESM.zip › Supplementary Data 4/Deletion/dhaK_deletion/dhaK_deletion_2.jpeg]

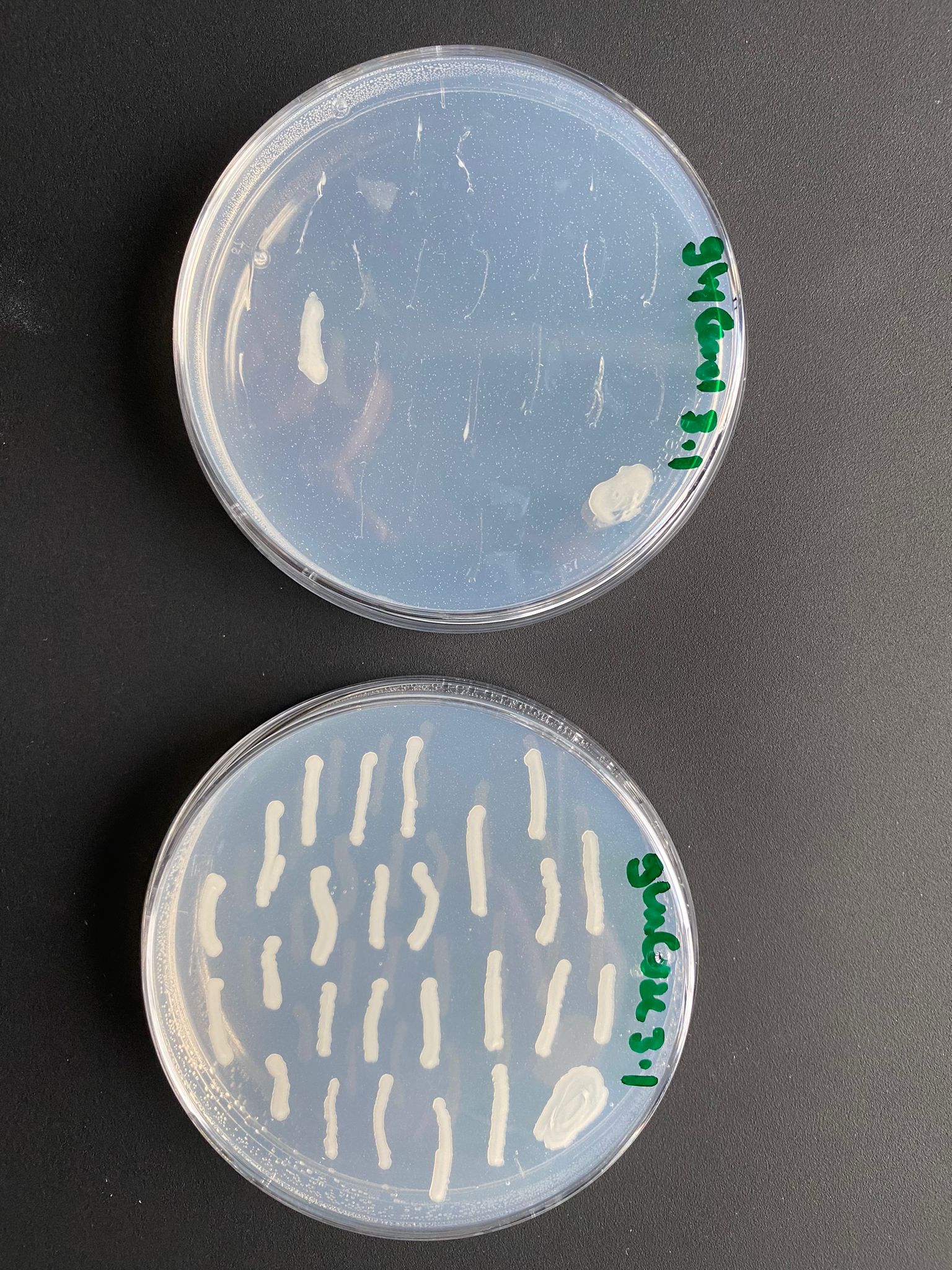

Supplement: Supplementary file 6 — Supplementary Data 4 [file 42003_2025_8934_MOESM6_ESM.zip › Supplementary Data 4/Deletion/dhaK_deletion/dhaK_deletion_3.jpeg]

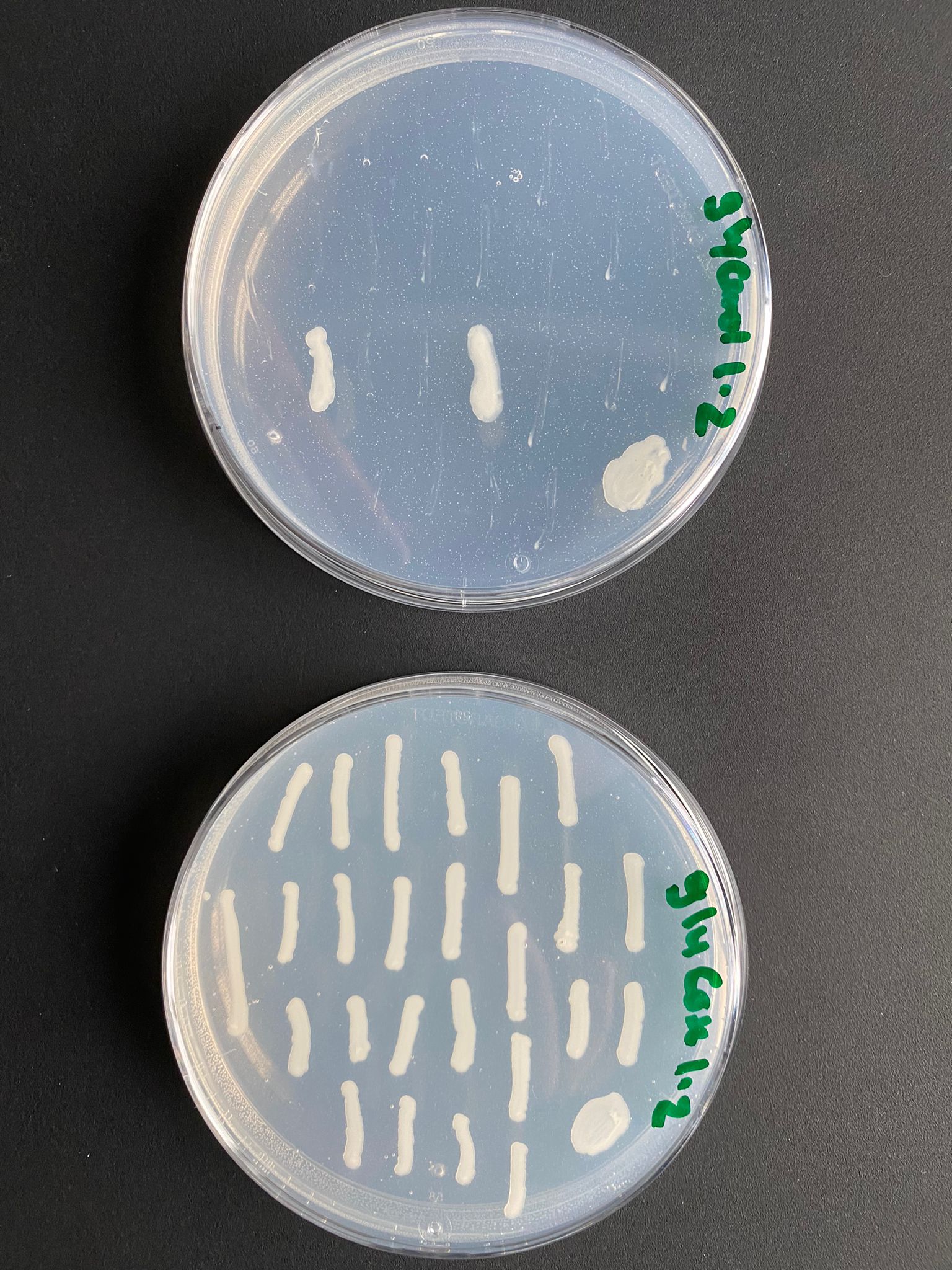

Supplement: Supplementary file 6 — Supplementary Data 4 [file 42003_2025_8934_MOESM6_ESM.zip › Supplementary Data 4/Deletion/dhaK_deletion/dhaK_deletion_4.jpeg]

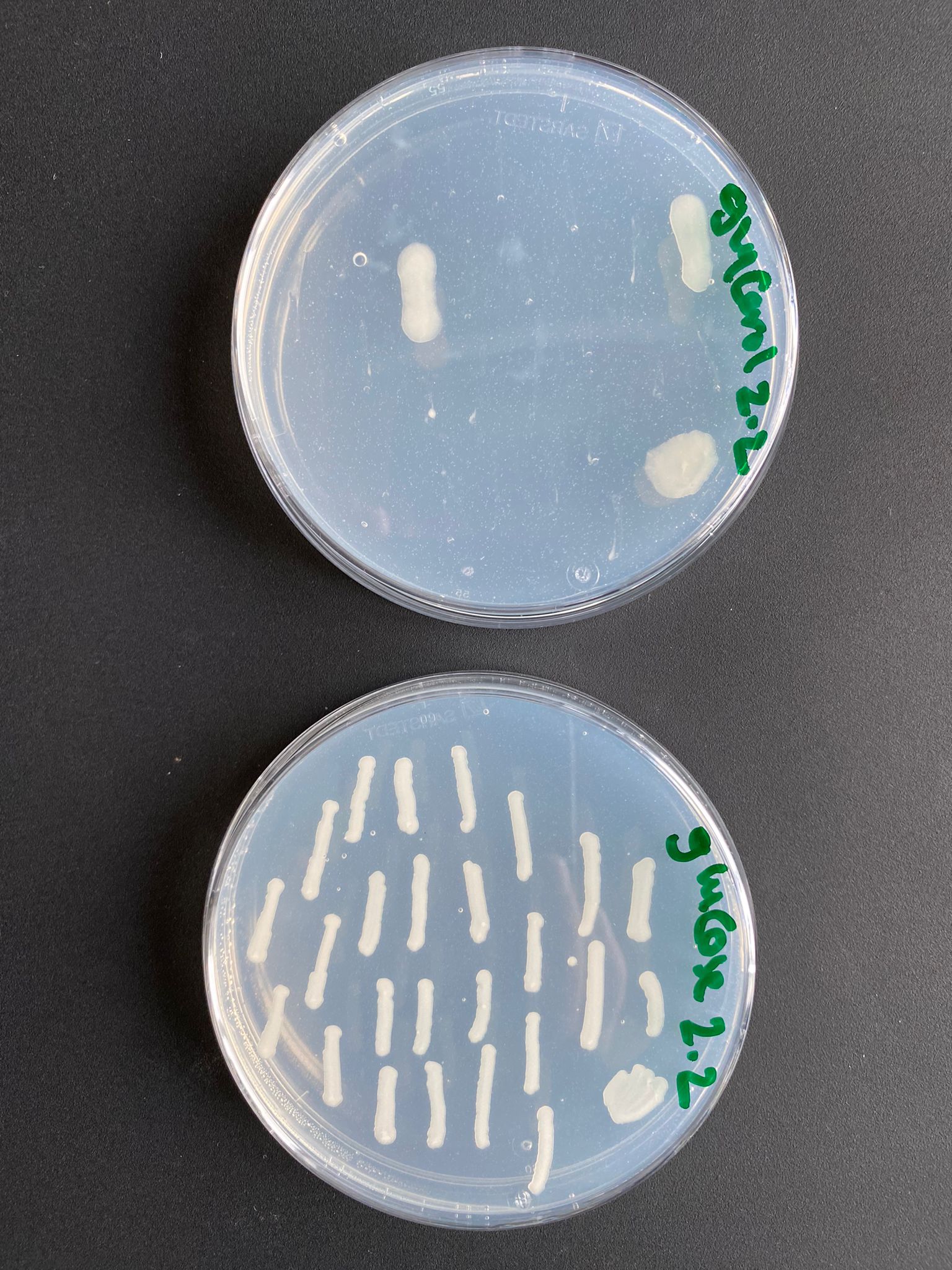

Supplement: Supplementary file 6 — Supplementary Data 4 [file 42003_2025_8934_MOESM6_ESM.zip › Supplementary Data 4/Deletion/dhaK_deletion/dhaK_deletion_5.jpeg]

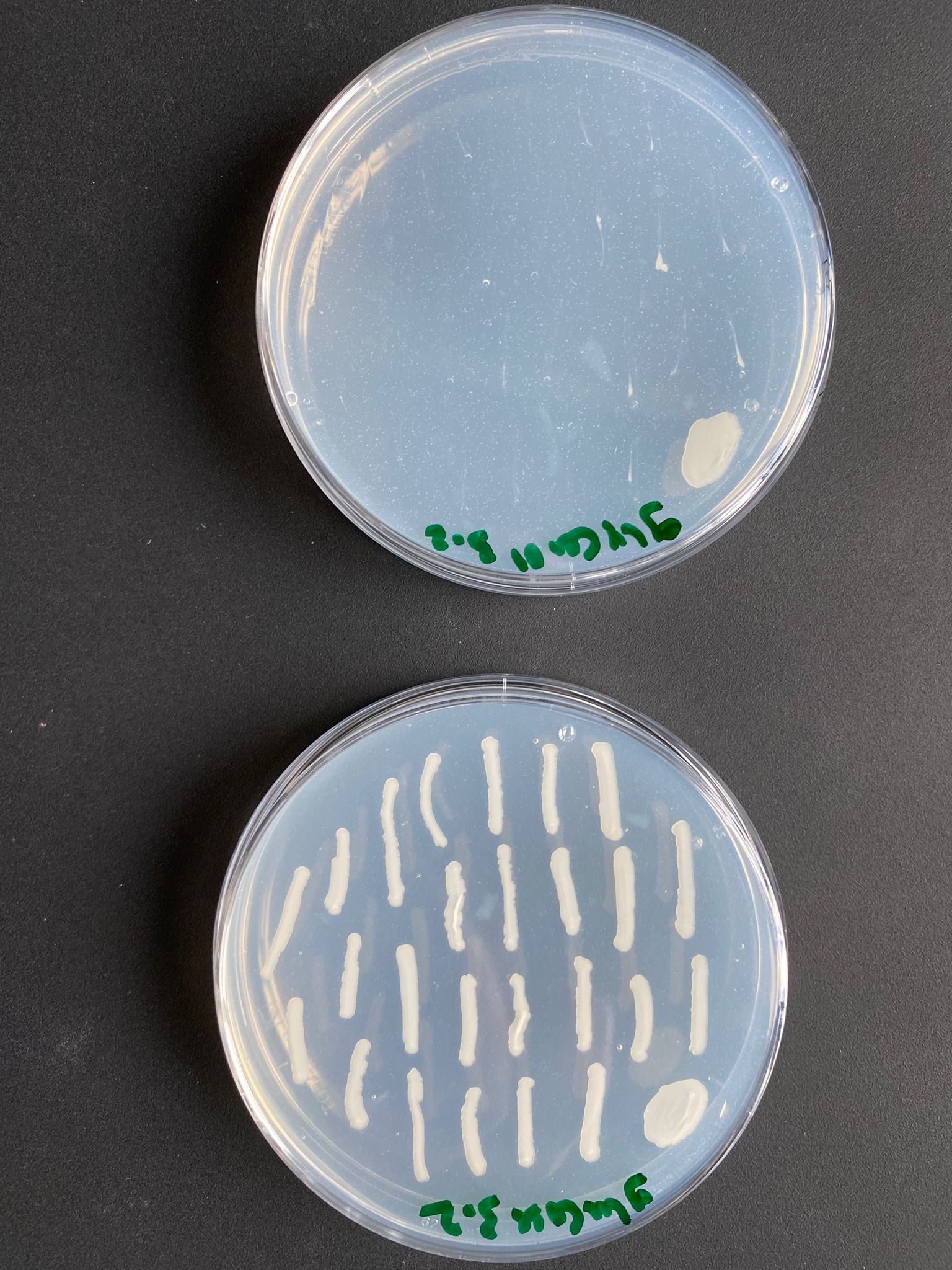

Supplement: Supplementary file 6 — Supplementary Data 4 [file 42003_2025_8934_MOESM6_ESM.zip › Supplementary Data 4/Deletion/dhaK_deletion/dhaK_deletion_6.jpeg]

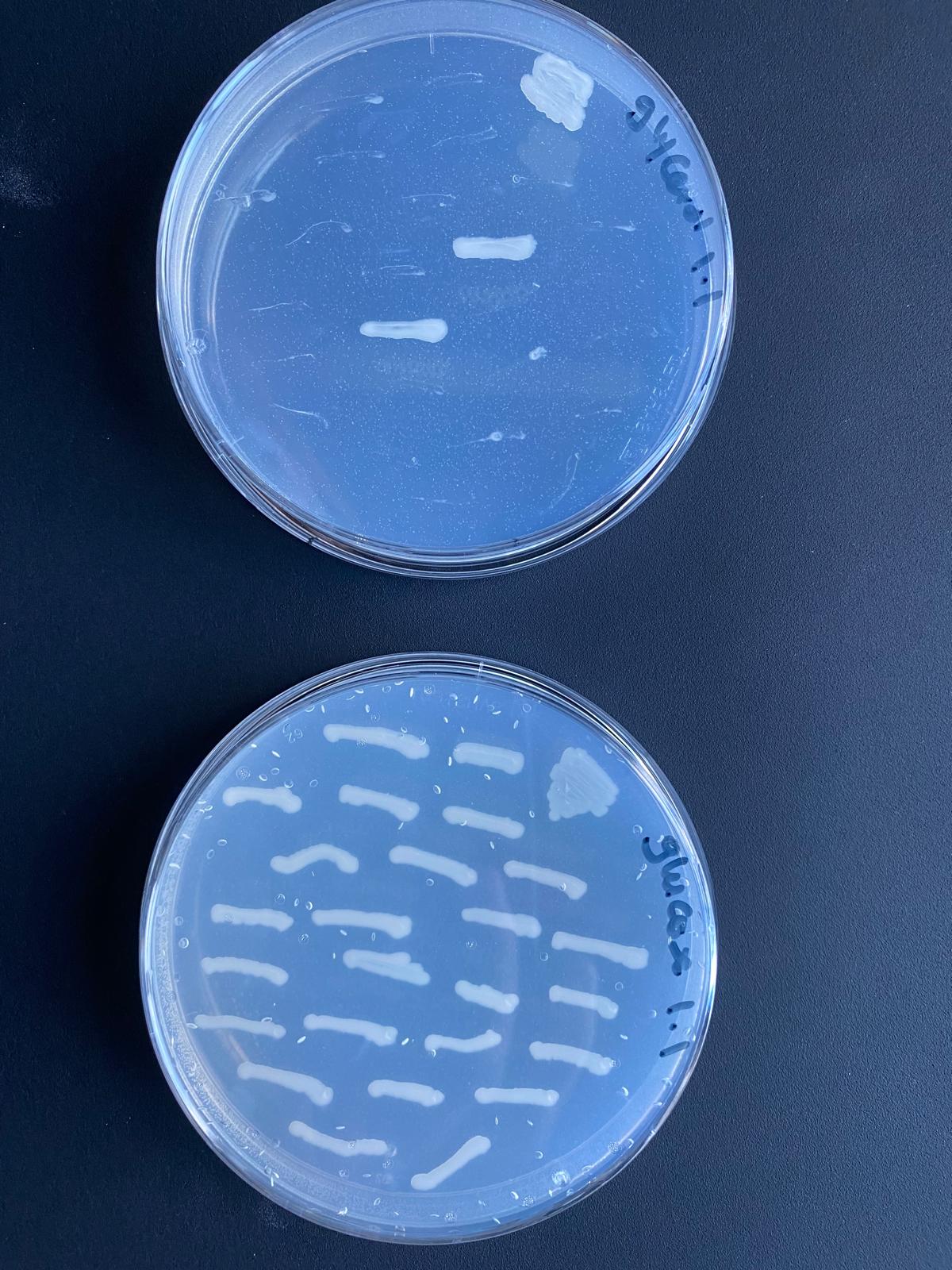

Supplement: Supplementary file 6 — Supplementary Data 4 [file 42003_2025_8934_MOESM6_ESM.zip › Supplementary Data 4/Deletion/glpK_deletion/glpK_deletion_1.jpeg]

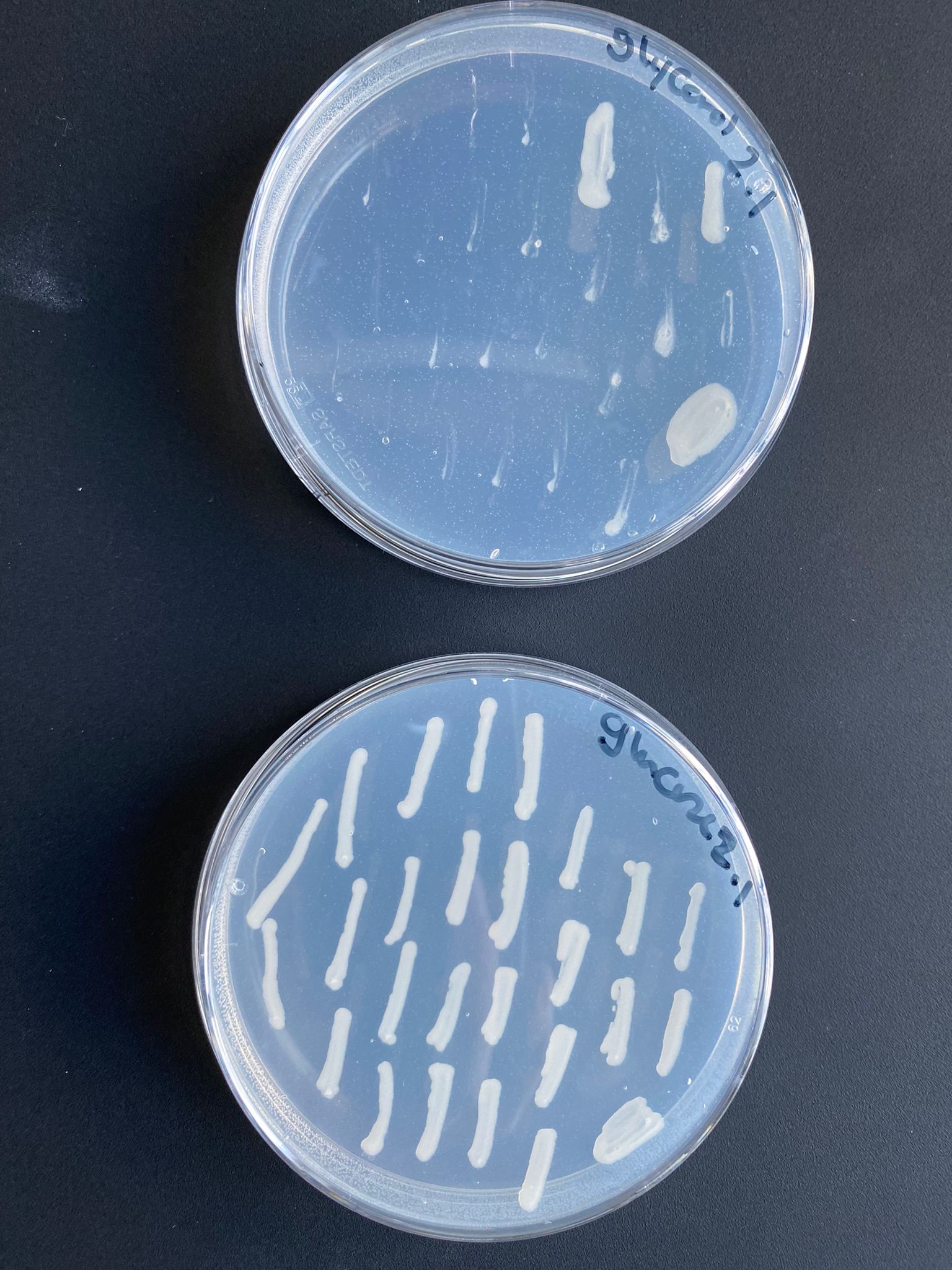

Supplement: Supplementary file 6 — Supplementary Data 4 [file 42003_2025_8934_MOESM6_ESM.zip › Supplementary Data 4/Deletion/glpK_deletion/glpK_deletion_2.jpeg]

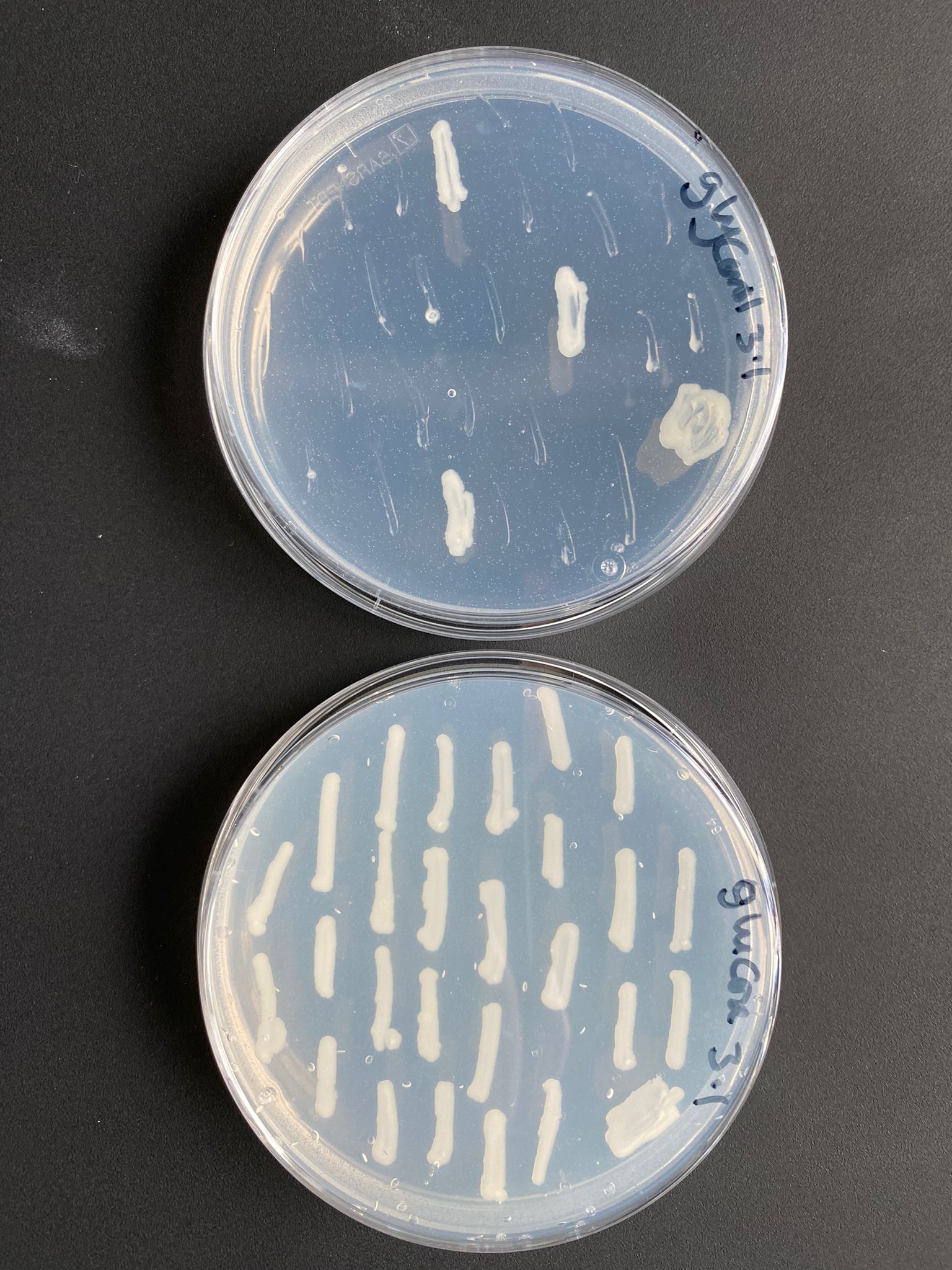

Supplement: Supplementary file 6 — Supplementary Data 4 [file 42003_2025_8934_MOESM6_ESM.zip › Supplementary Data 4/Deletion/glpK_deletion/glpK_deletion_3.jpeg]

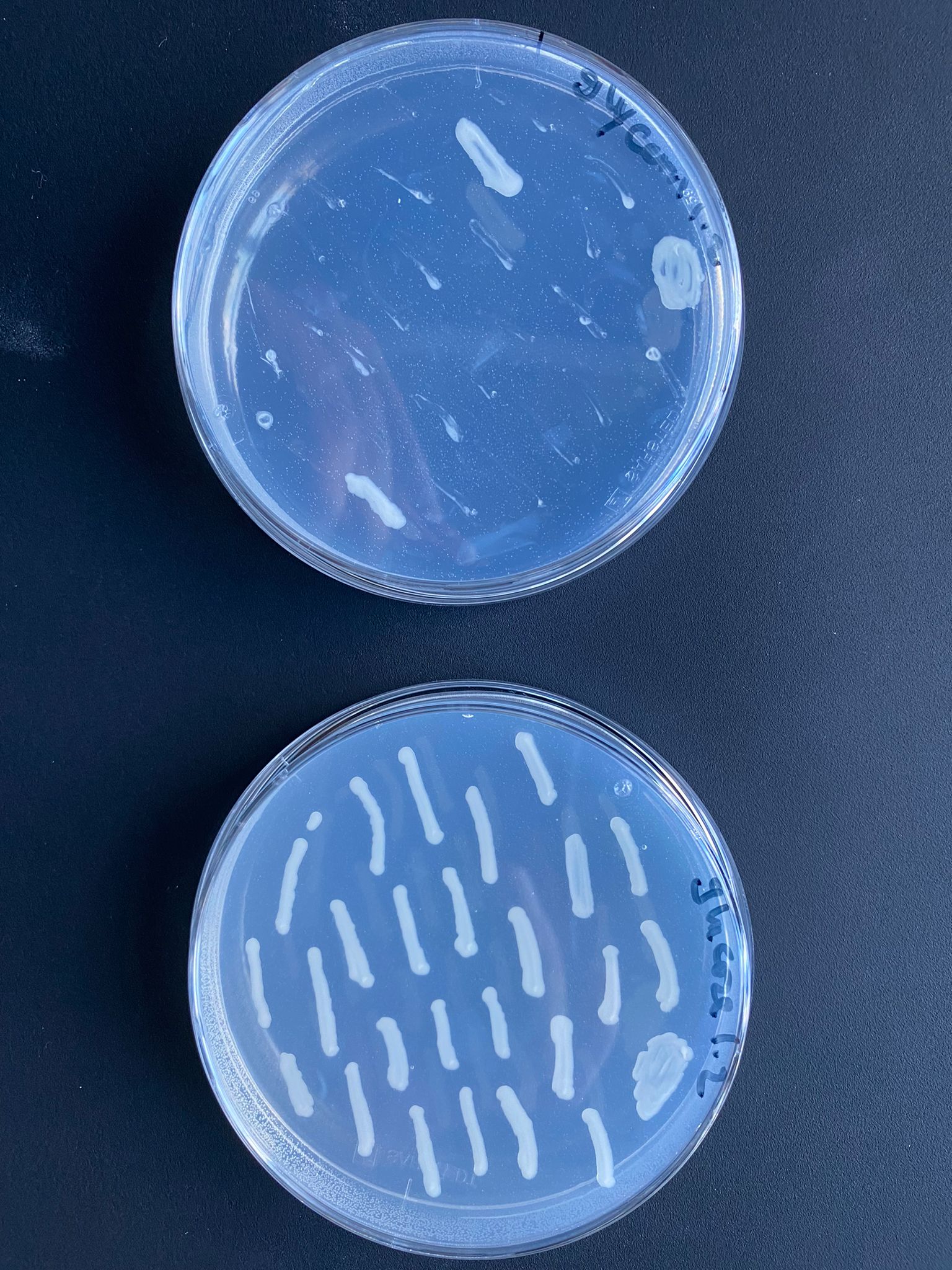

Supplement: Supplementary file 6 — Supplementary Data 4 [file 42003_2025_8934_MOESM6_ESM.zip › Supplementary Data 4/Deletion/glpK_deletion/glpK_deletion_4.jpeg]

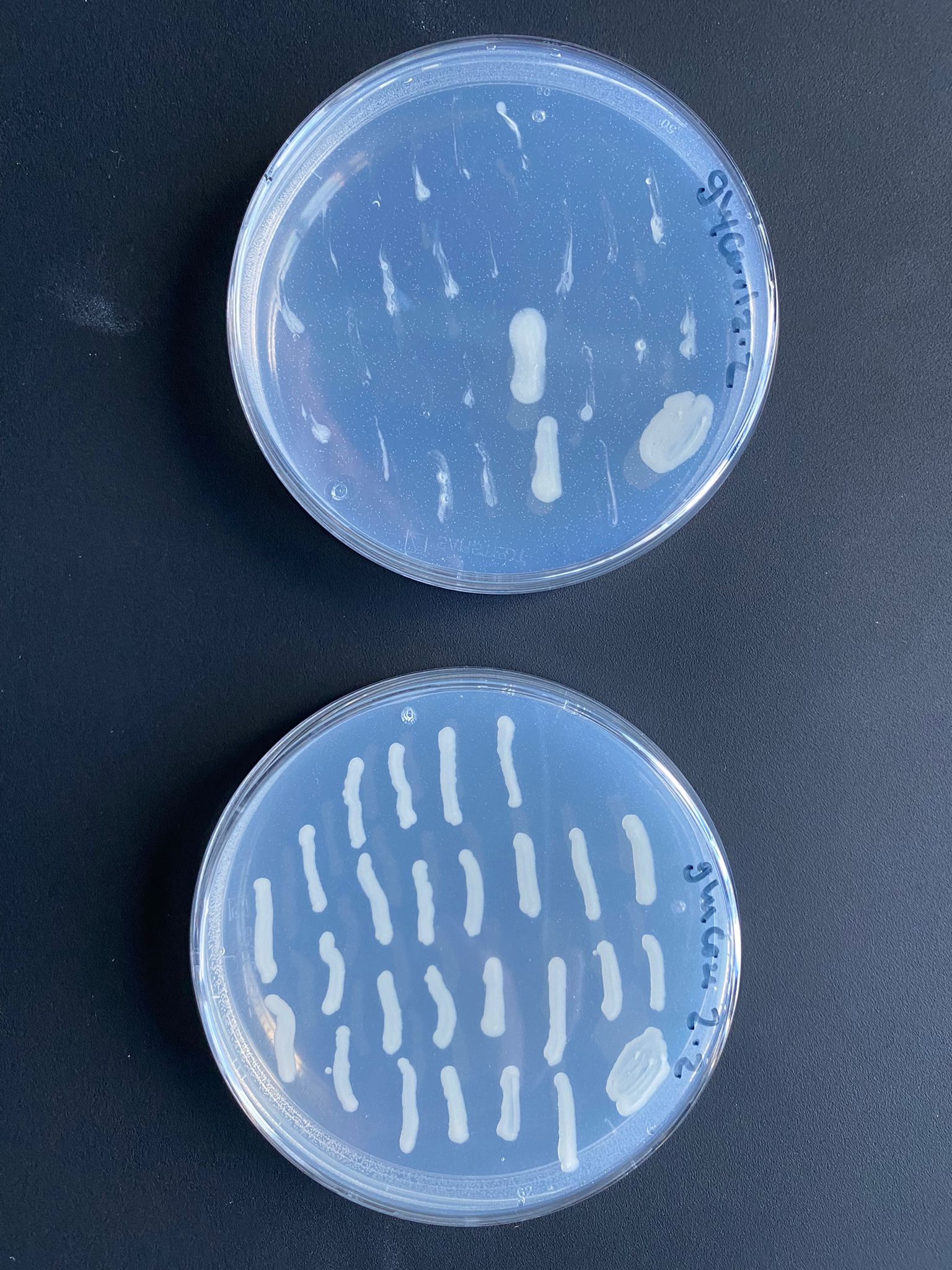

Supplement: Supplementary file 6 — Supplementary Data 4 [file 42003_2025_8934_MOESM6_ESM.zip › Supplementary Data 4/Deletion/glpK_deletion/glpK_deletion_5.jpeg]

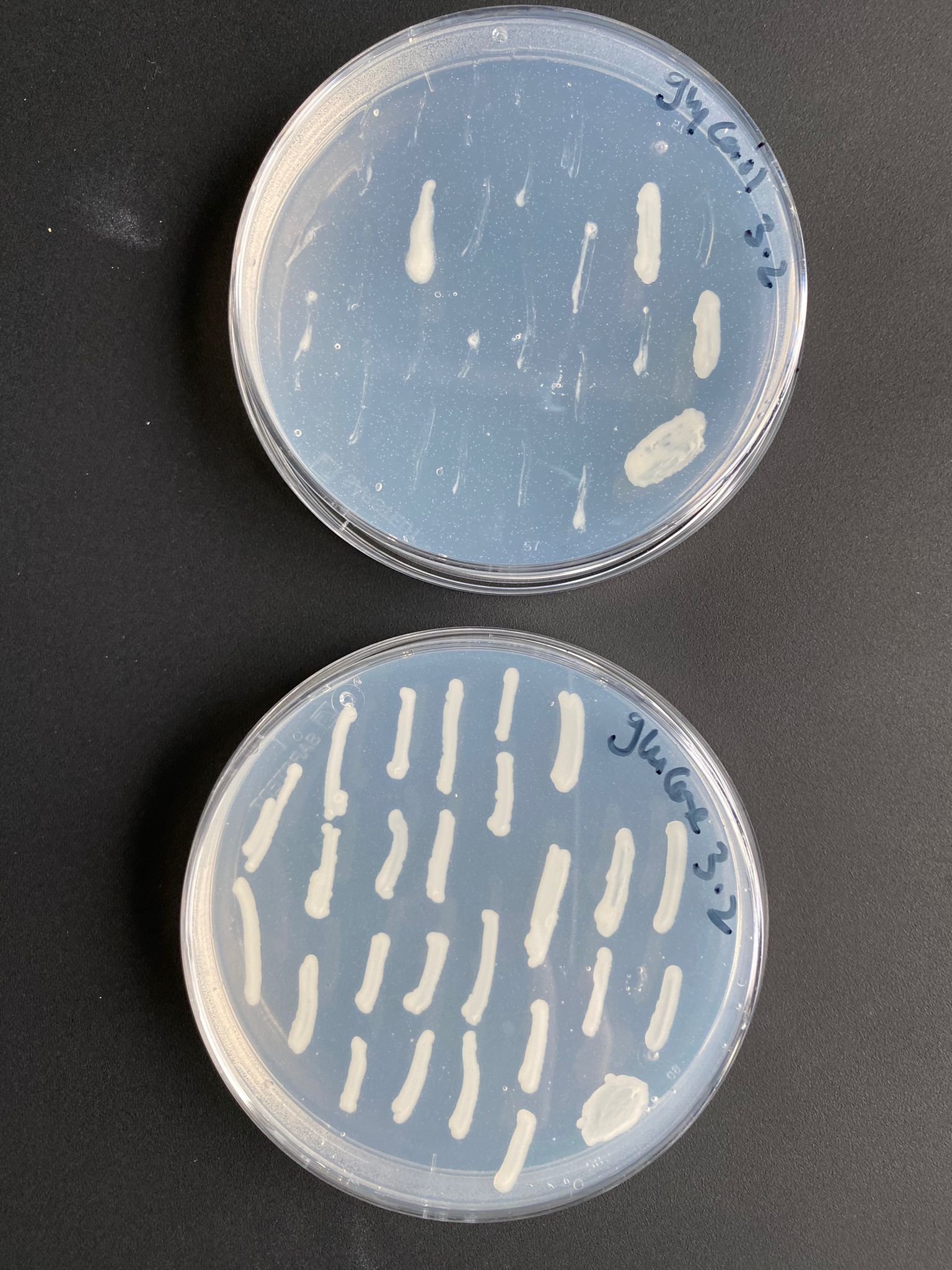

Supplement: Supplementary file 6 — Supplementary Data 4 [file 42003_2025_8934_MOESM6_ESM.zip › Supplementary Data 4/Deletion/glpK_deletion/glpK_deletion_6.jpeg]

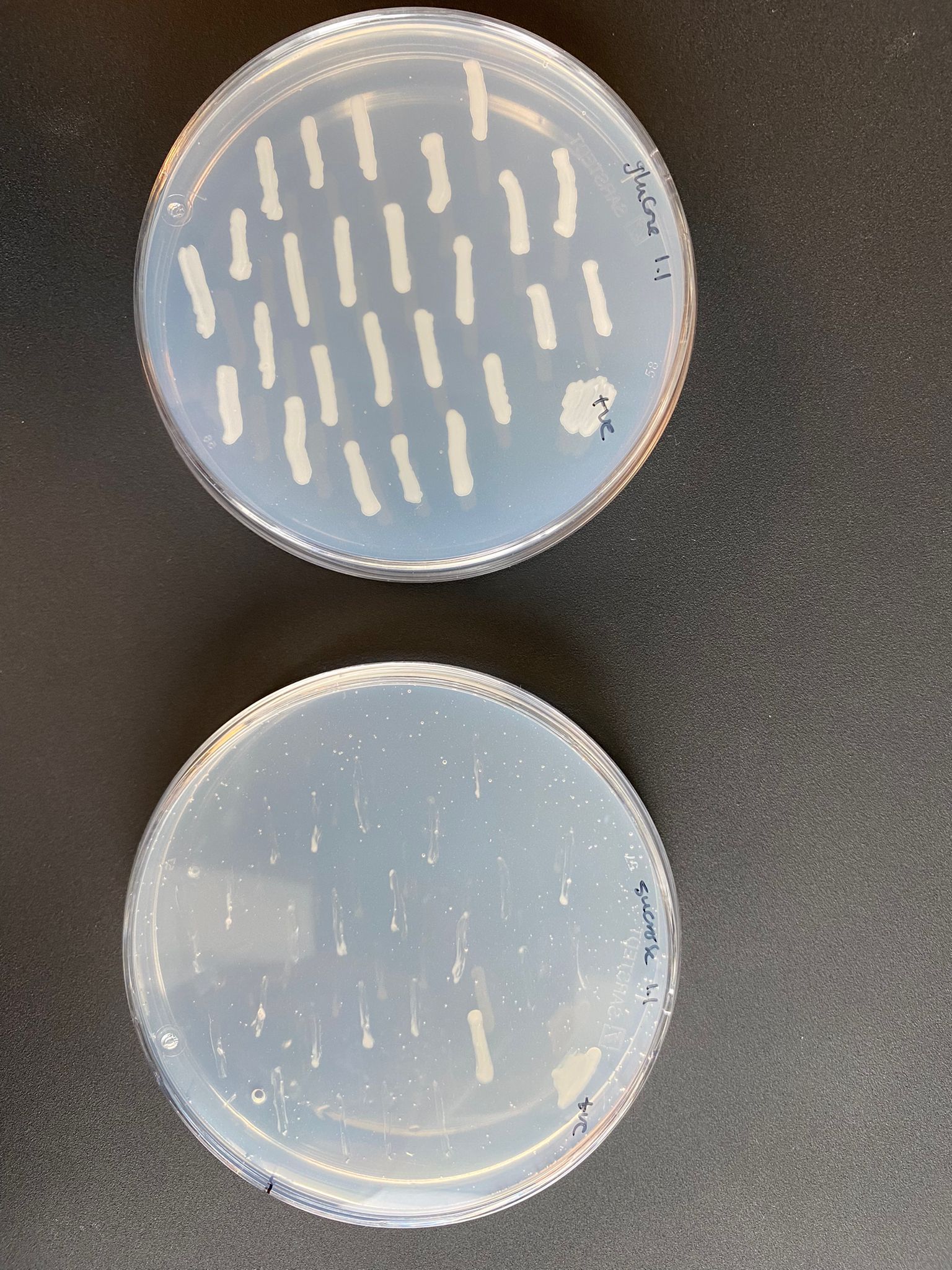

Supplement: Supplementary file 6 — Supplementary Data 4 [file 42003_2025_8934_MOESM6_ESM.zip › Supplementary Data 4/Deletion/scrK_deletion/scrK_deletion_1.jpeg]

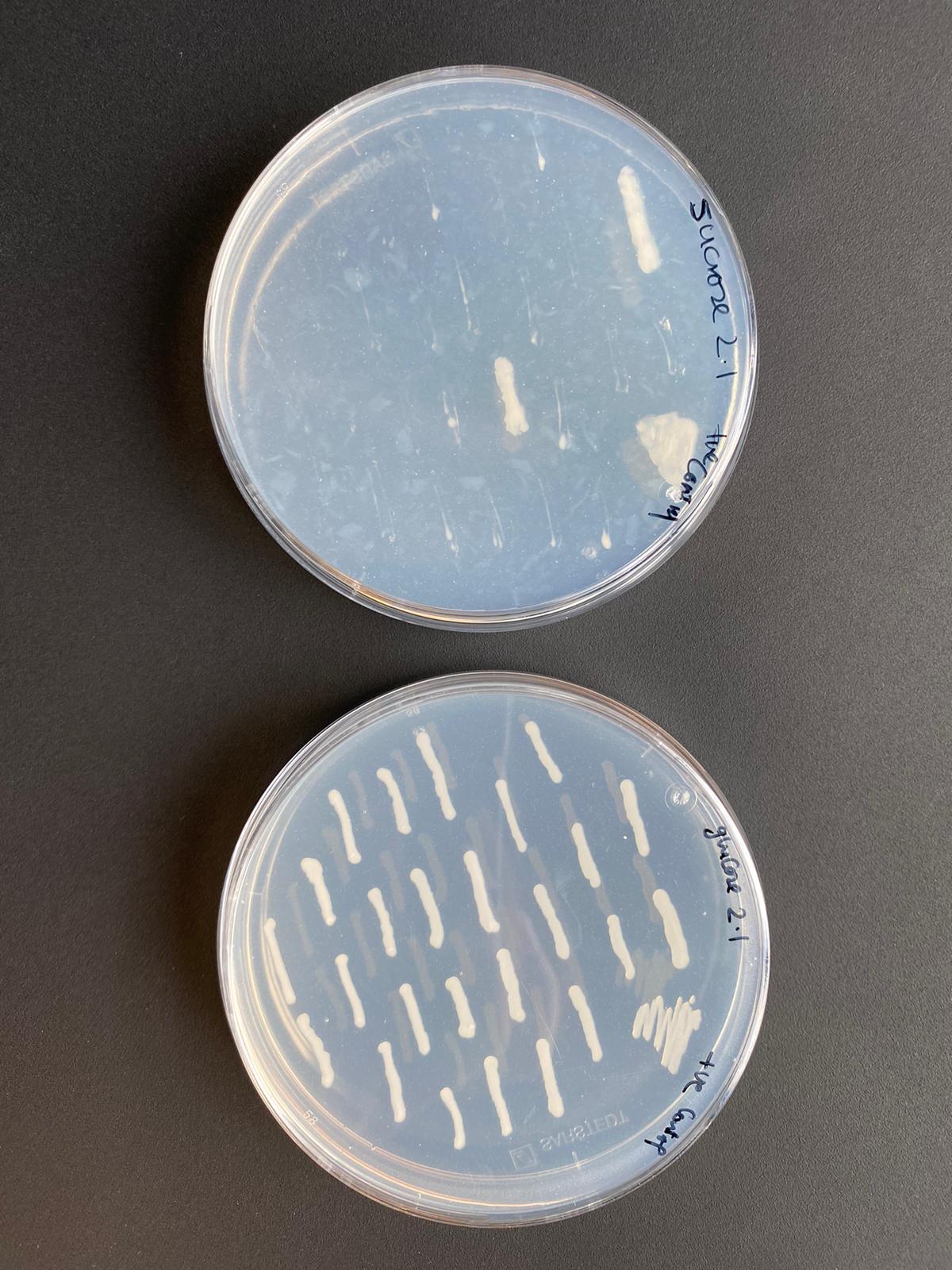

Supplement: Supplementary file 6 — Supplementary Data 4 [file 42003_2025_8934_MOESM6_ESM.zip › Supplementary Data 4/Deletion/scrK_deletion/scrK_deletion_2.jpeg]

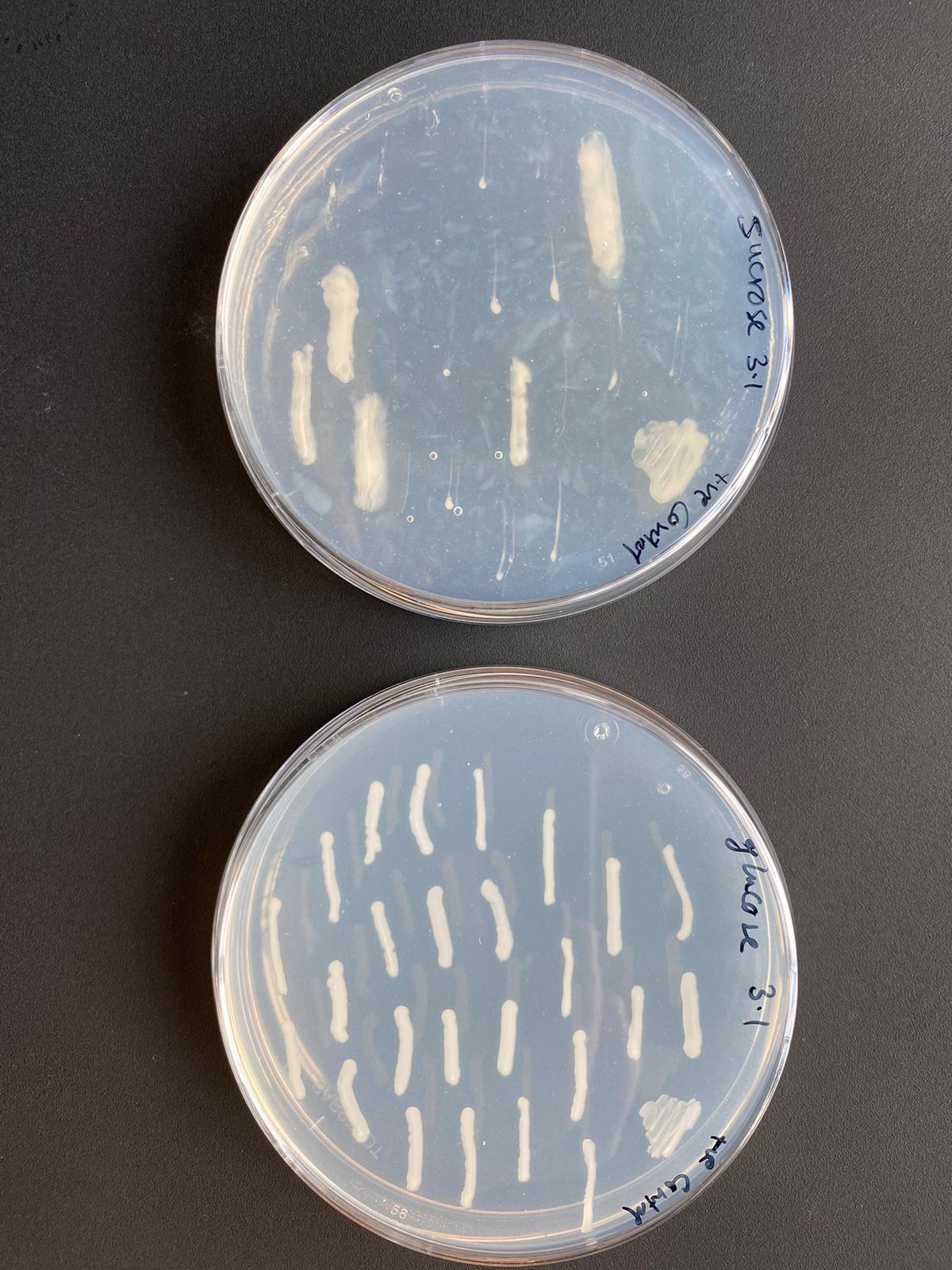

Supplement: Supplementary file 6 — Supplementary Data 4 [file 42003_2025_8934_MOESM6_ESM.zip › Supplementary Data 4/Deletion/scrK_deletion/scrK_deletion_3.jpeg]

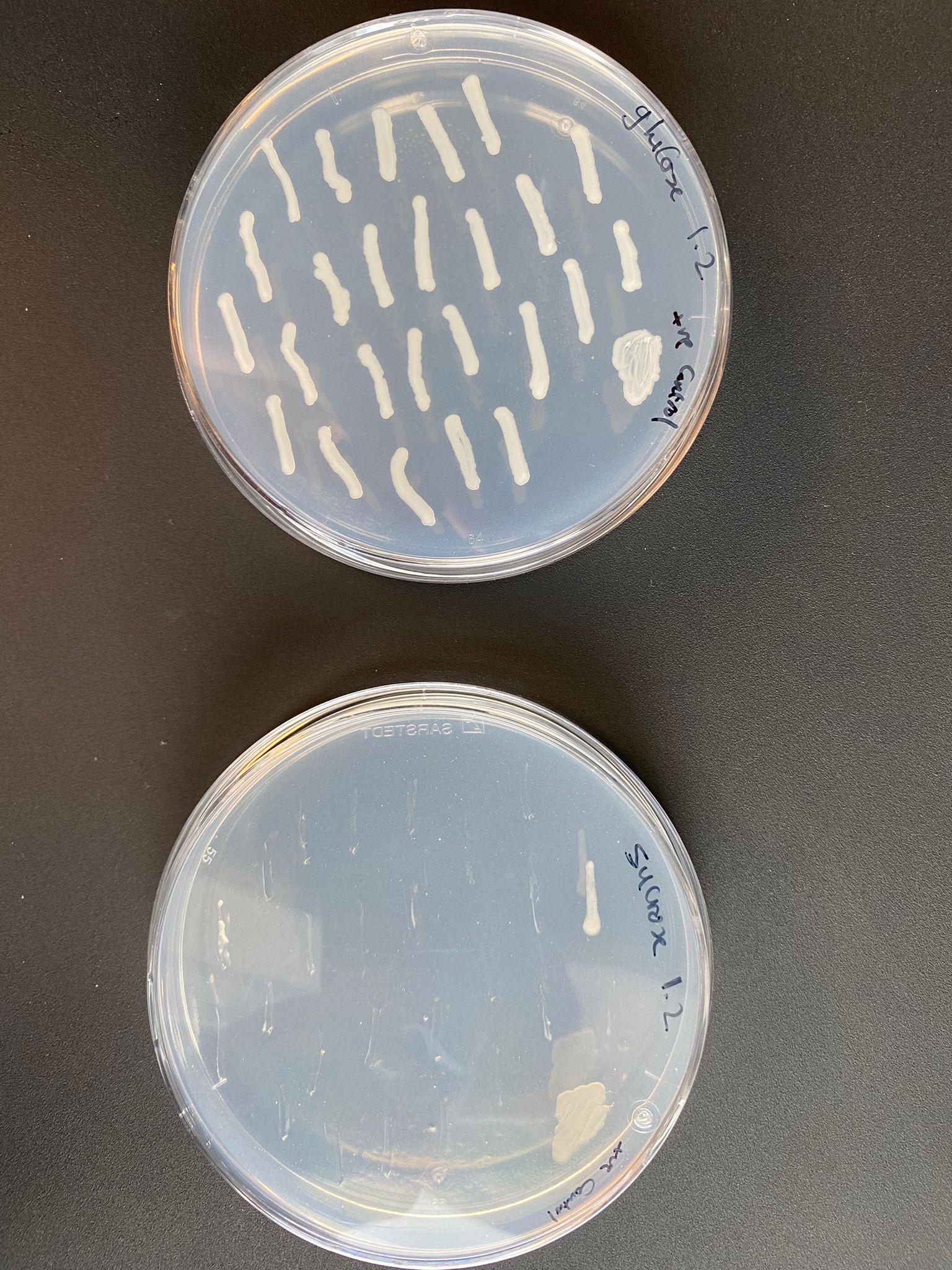

Supplement: Supplementary file 6 — Supplementary Data 4 [file 42003_2025_8934_MOESM6_ESM.zip › Supplementary Data 4/Deletion/scrK_deletion/scrK_deletion_4.jpeg]

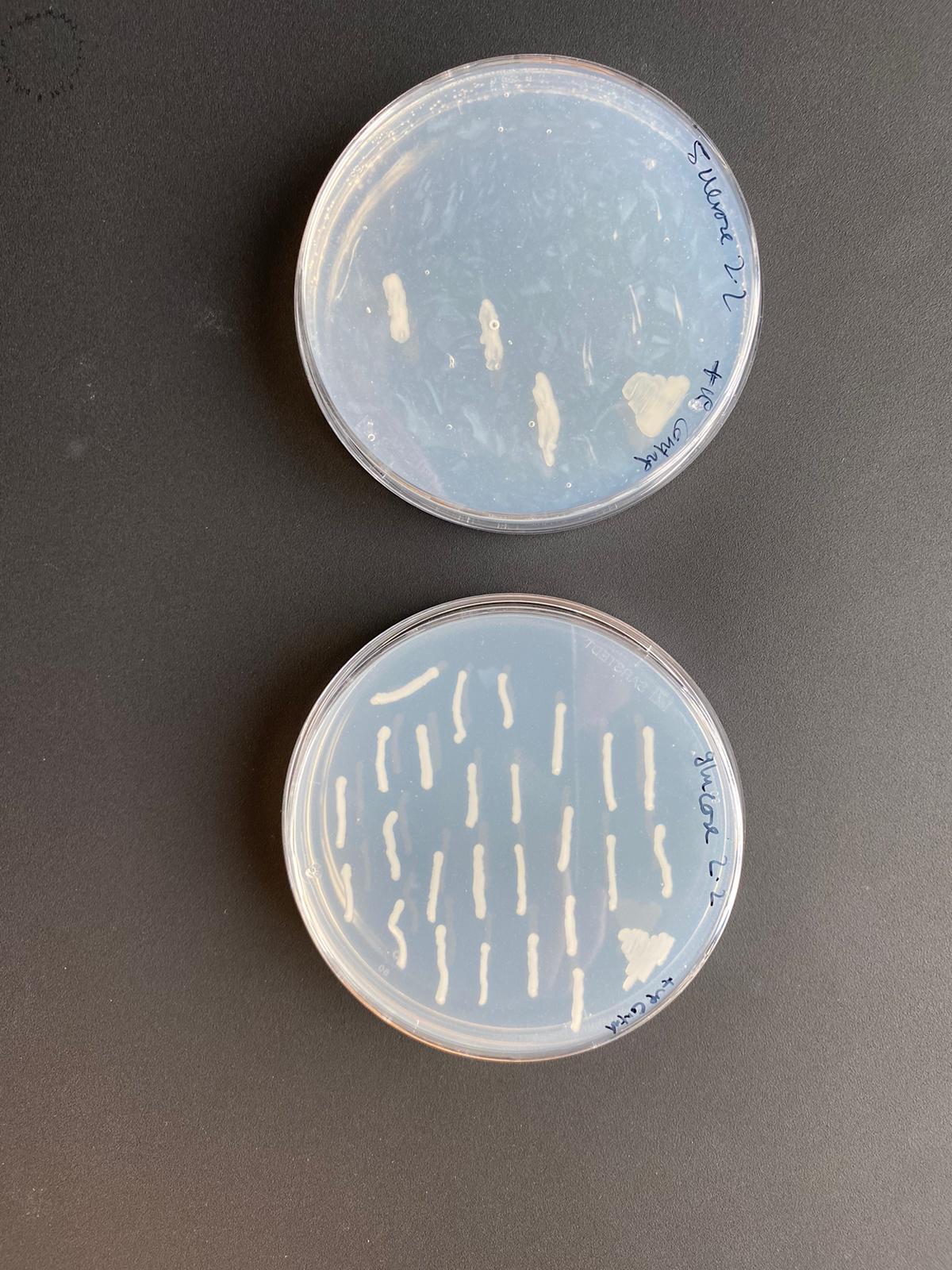

Supplement: Supplementary file 6 — Supplementary Data 4 [file 42003_2025_8934_MOESM6_ESM.zip › Supplementary Data 4/Deletion/scrK_deletion/scrK_deletion_5.jpeg]

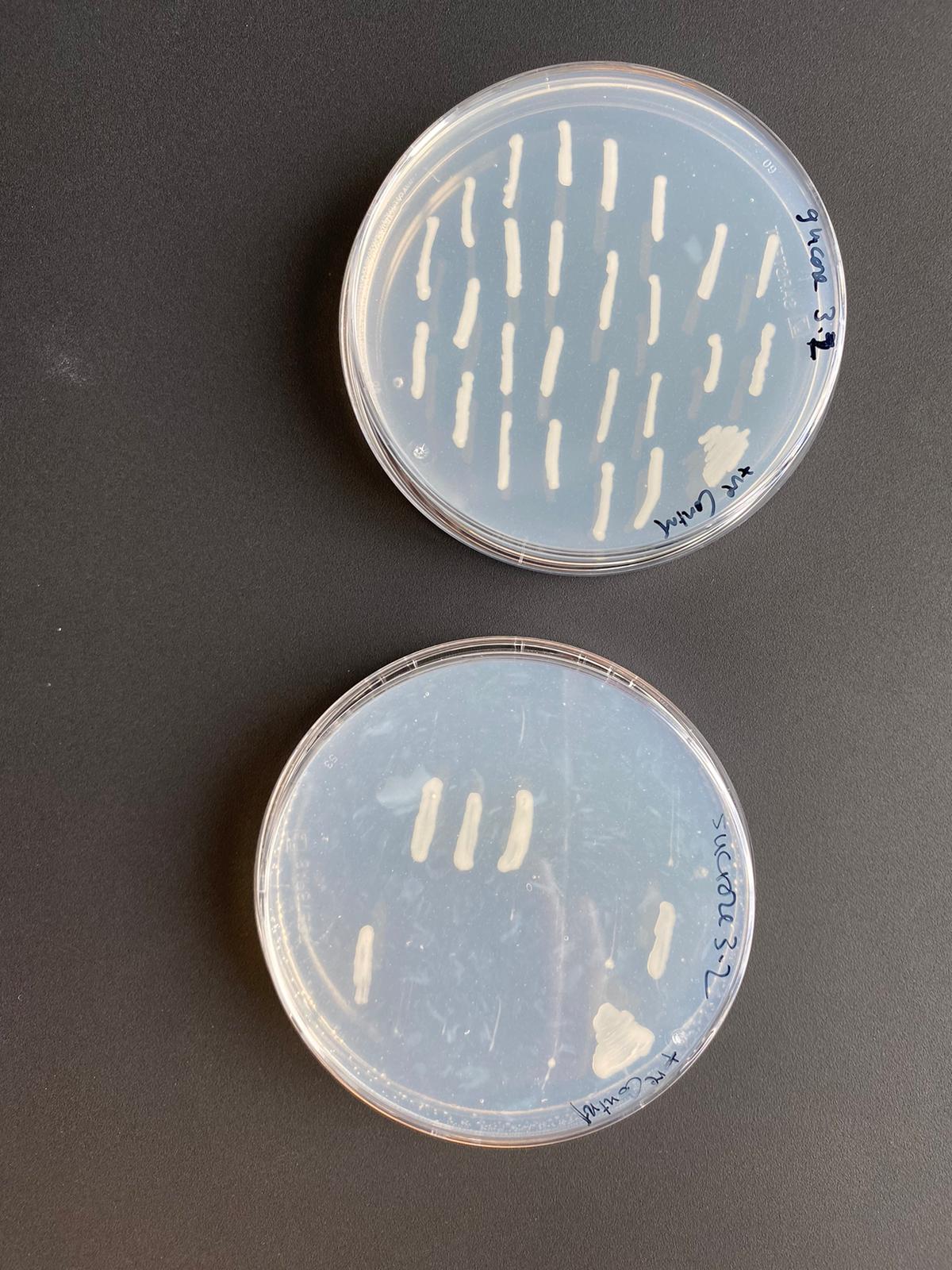

Supplement: Supplementary file 6 — Supplementary Data 4 [file 42003_2025_8934_MOESM6_ESM.zip › Supplementary Data 4/Deletion/scrK_deletion/scrK_deletion_6.jpeg]

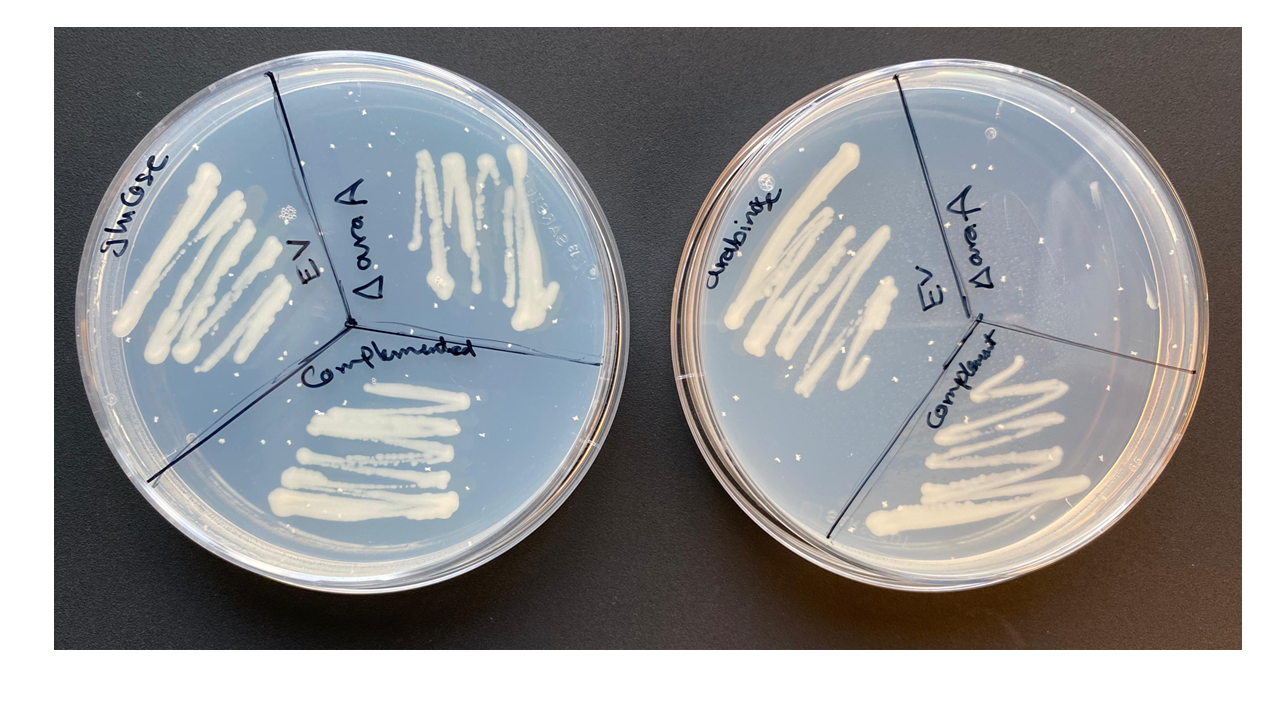

Supplement: Supplementary file 6 — Supplementary Data 4 [file 42003_2025_8934_MOESM6_ESM.zip › Supplementary Data 4/complementation/araA_del_complement_1.TIF]

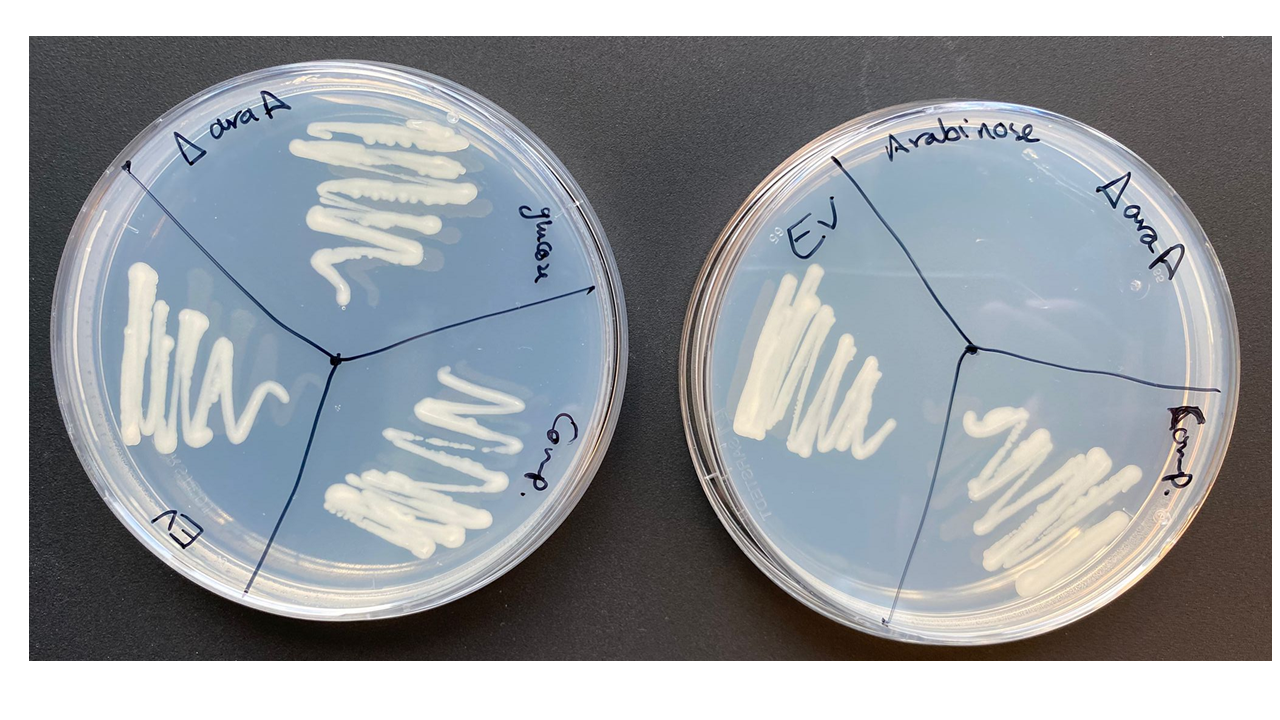

Supplement: Supplementary file 6 — Supplementary Data 4 [file 42003_2025_8934_MOESM6_ESM.zip › Supplementary Data 4/complementation/araA_del_complement_2.TIF]

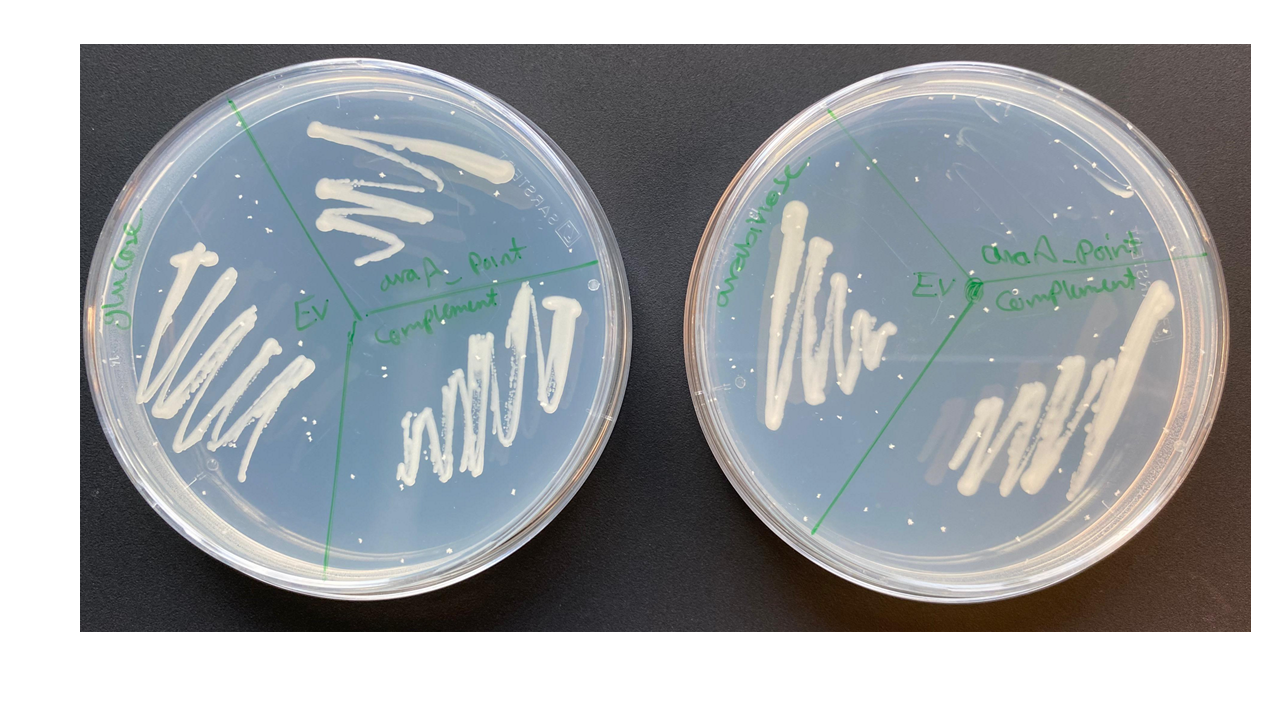

Supplement: Supplementary file 6 — Supplementary Data 4 [file 42003_2025_8934_MOESM6_ESM.zip › Supplementary Data 4/complementation/araA_point_complement_1.TIF]

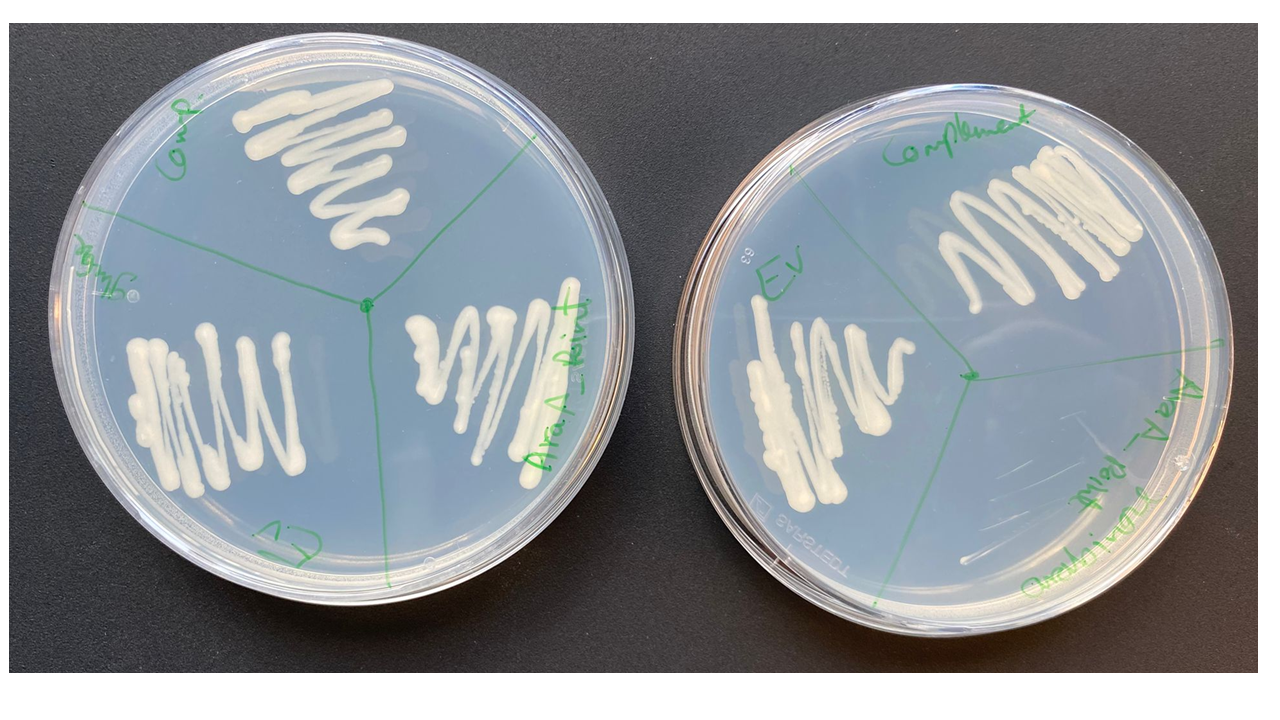

Supplement: Supplementary file 6 — Supplementary Data 4 [file 42003_2025_8934_MOESM6_ESM.zip › Supplementary Data 4/complementation/araA_point_complement_2.TIF]

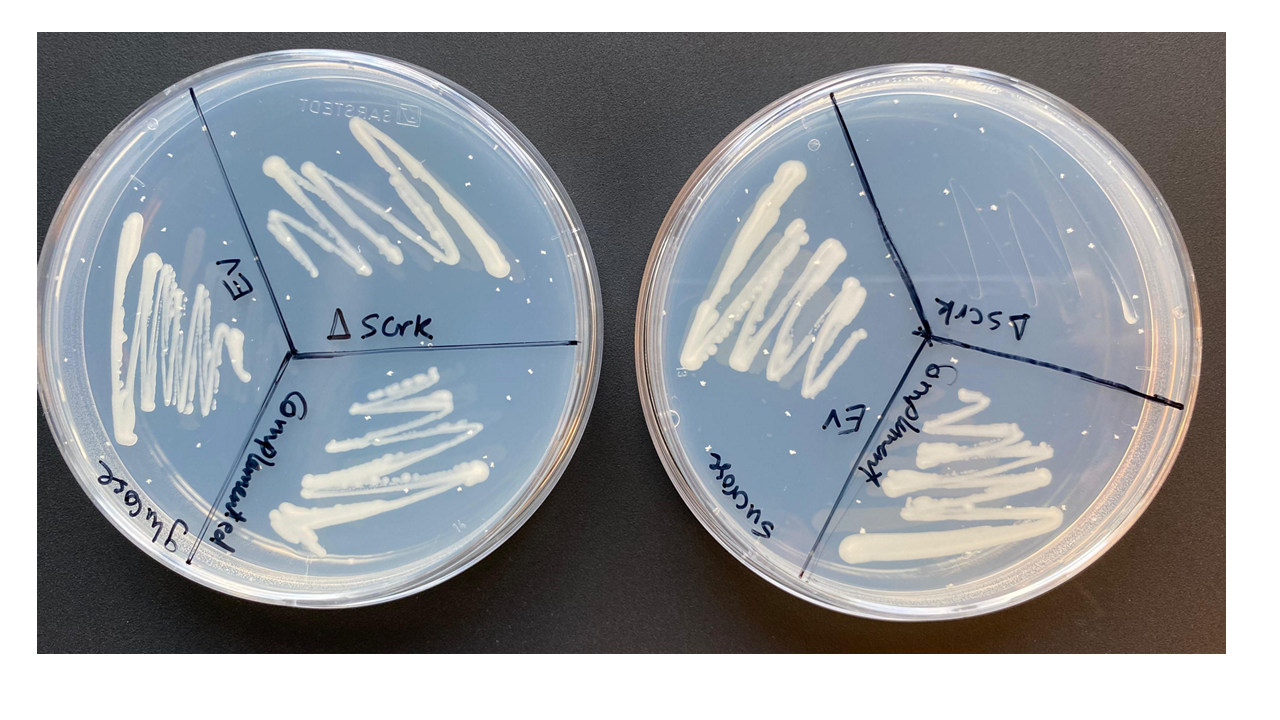

Supplement: Supplementary file 6 — Supplementary Data 4 [file 42003_2025_8934_MOESM6_ESM.zip › Supplementary Data 4/complementation/scrK_del_complement_1.TIF]

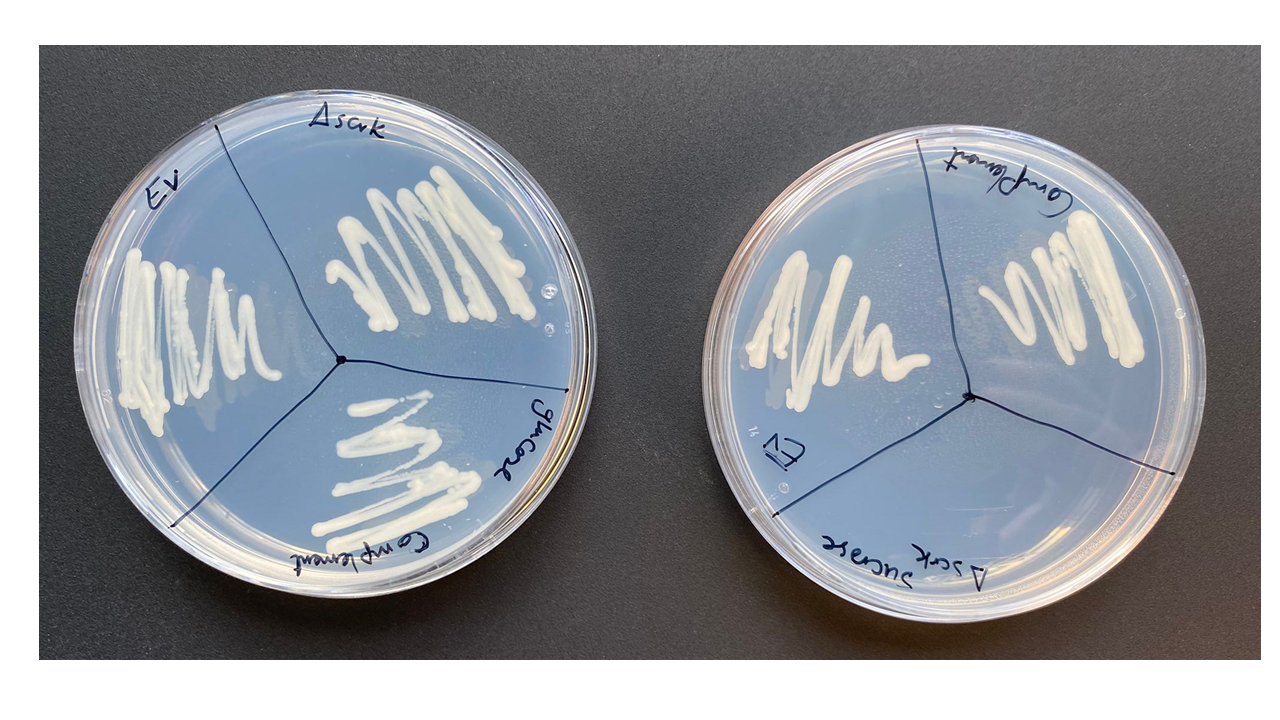

Supplement: Supplementary file 6 — Supplementary Data 4 [file 42003_2025_8934_MOESM6_ESM.zip › Supplementary Data 4/complementation/scrK_del_complement_2.TIF]

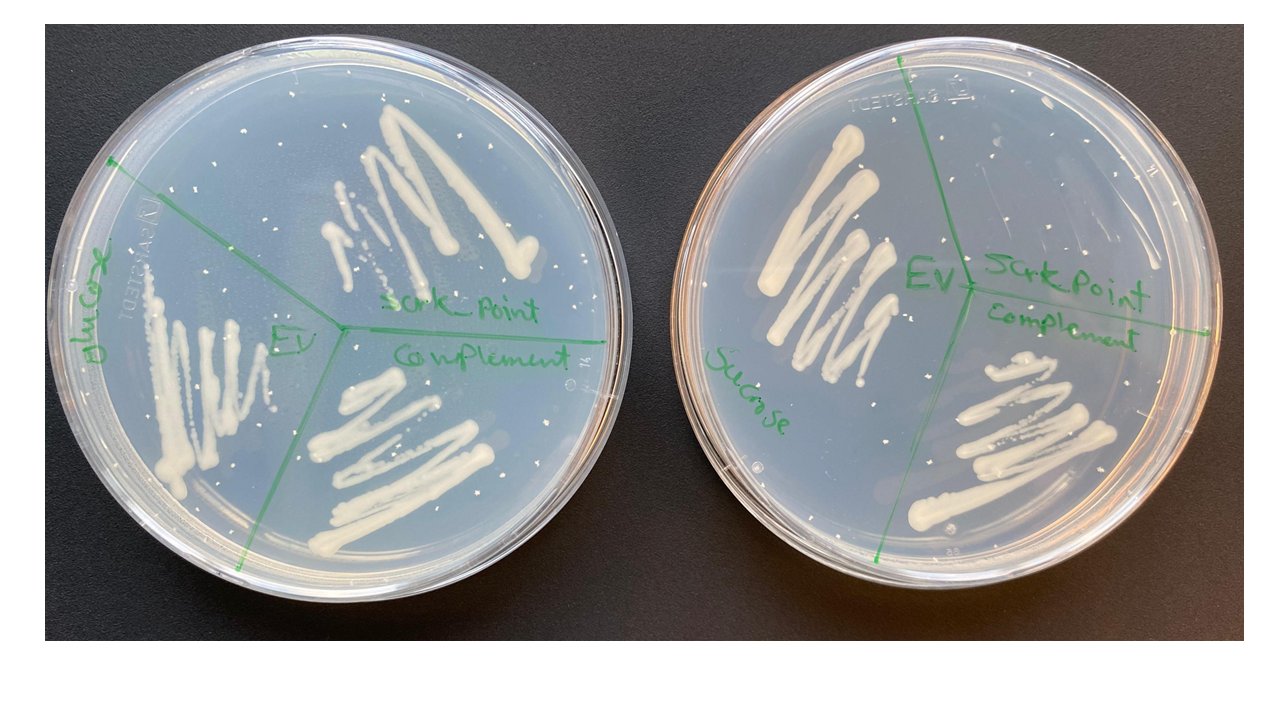

Supplement: Supplementary file 6 — Supplementary Data 4 [file 42003_2025_8934_MOESM6_ESM.zip › Supplementary Data 4/complementation/scrK_point_complement_1.TIF]

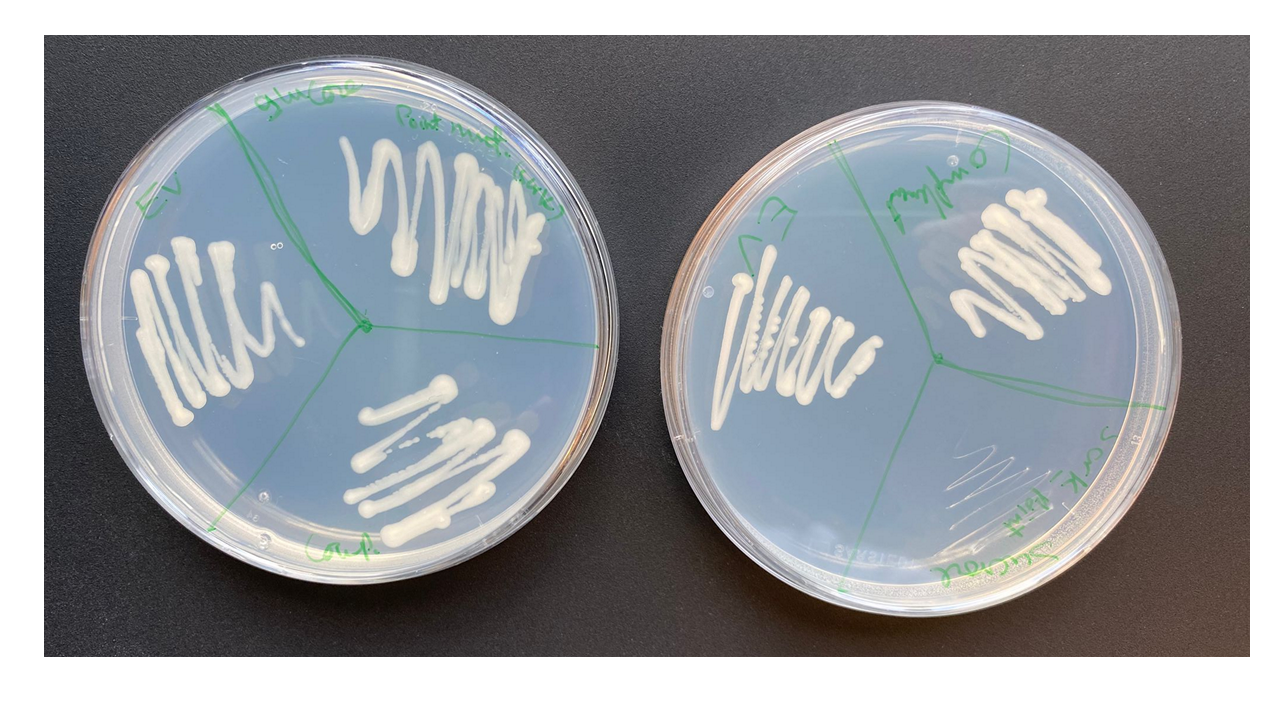

Supplement: Supplementary file 6 — Supplementary Data 4 [file 42003_2025_8934_MOESM6_ESM.zip › Supplementary Data 4/complementation/scrK_point_complement_2.TIF]

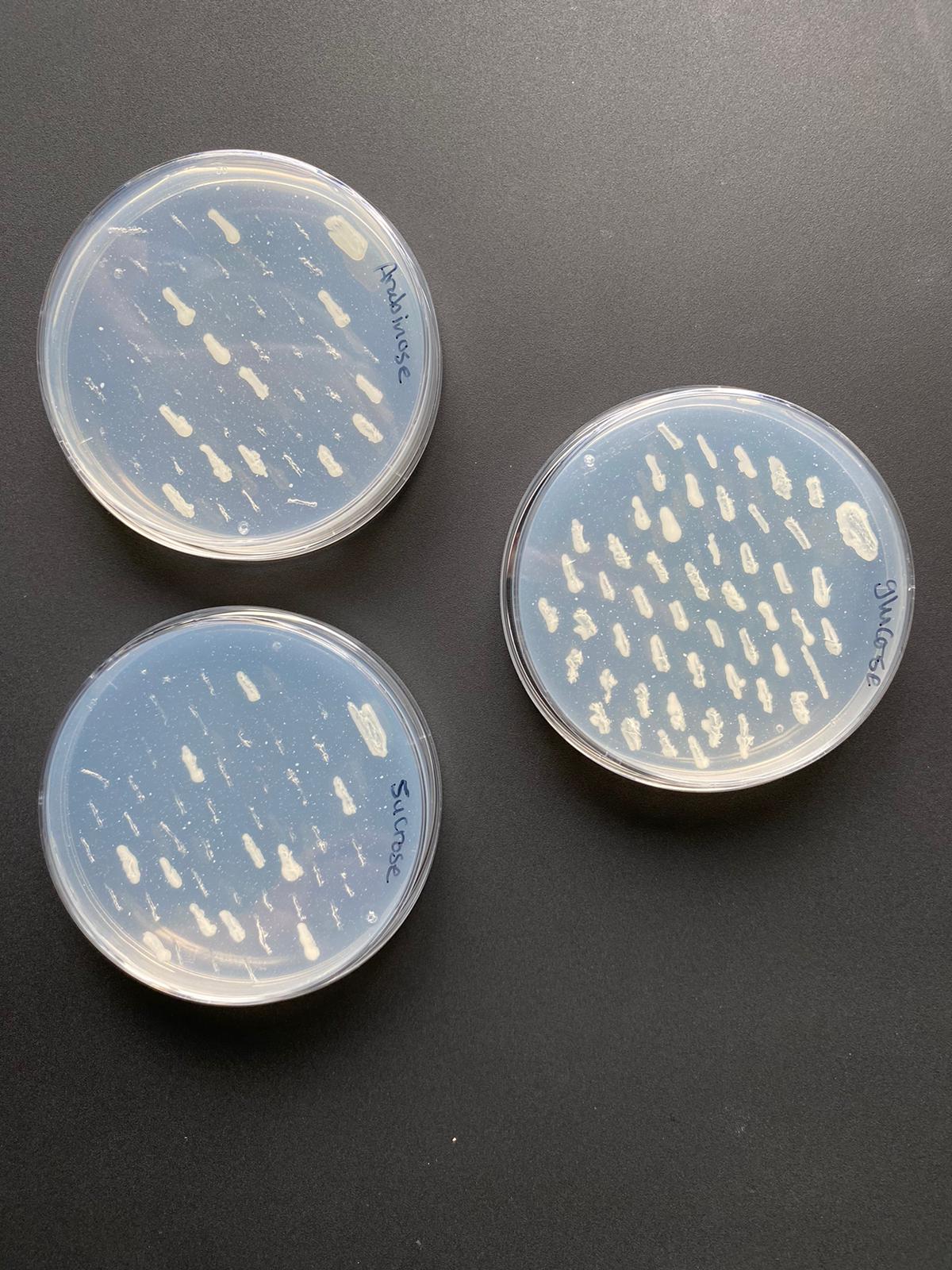

Supplement: Supplementary file 6 — Supplementary Data 4 [file 42003_2025_8934_MOESM6_ESM.zip › Supplementary Data 4/multi-deletions/multideletion_araAandscrK.jpeg]

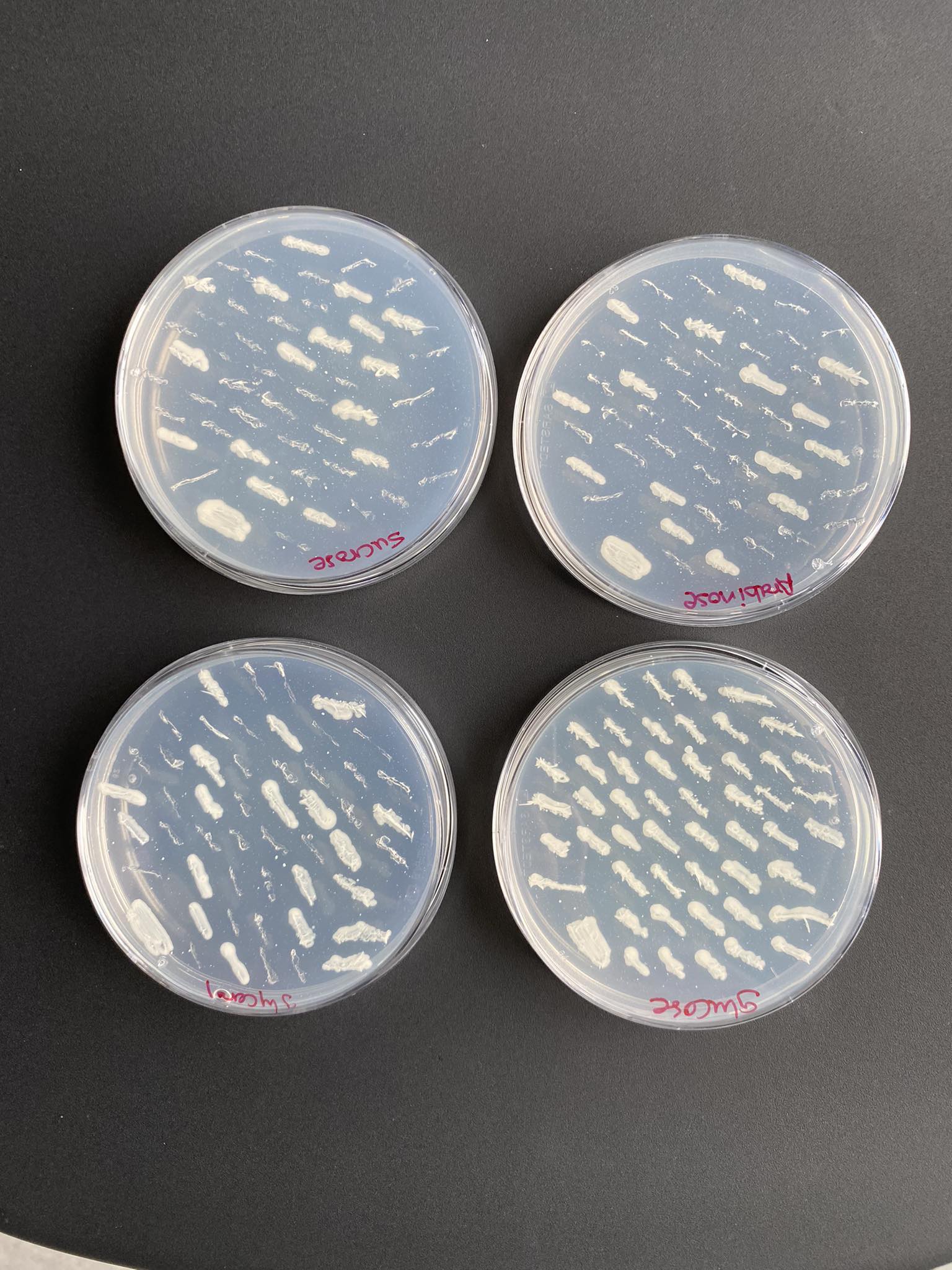

Supplement: Supplementary file 6 — Supplementary Data 4 [file 42003_2025_8934_MOESM6_ESM.zip › Supplementary Data 4/multi-deletions/multideletions_3_araA,scrk, and dhaK.jpg]

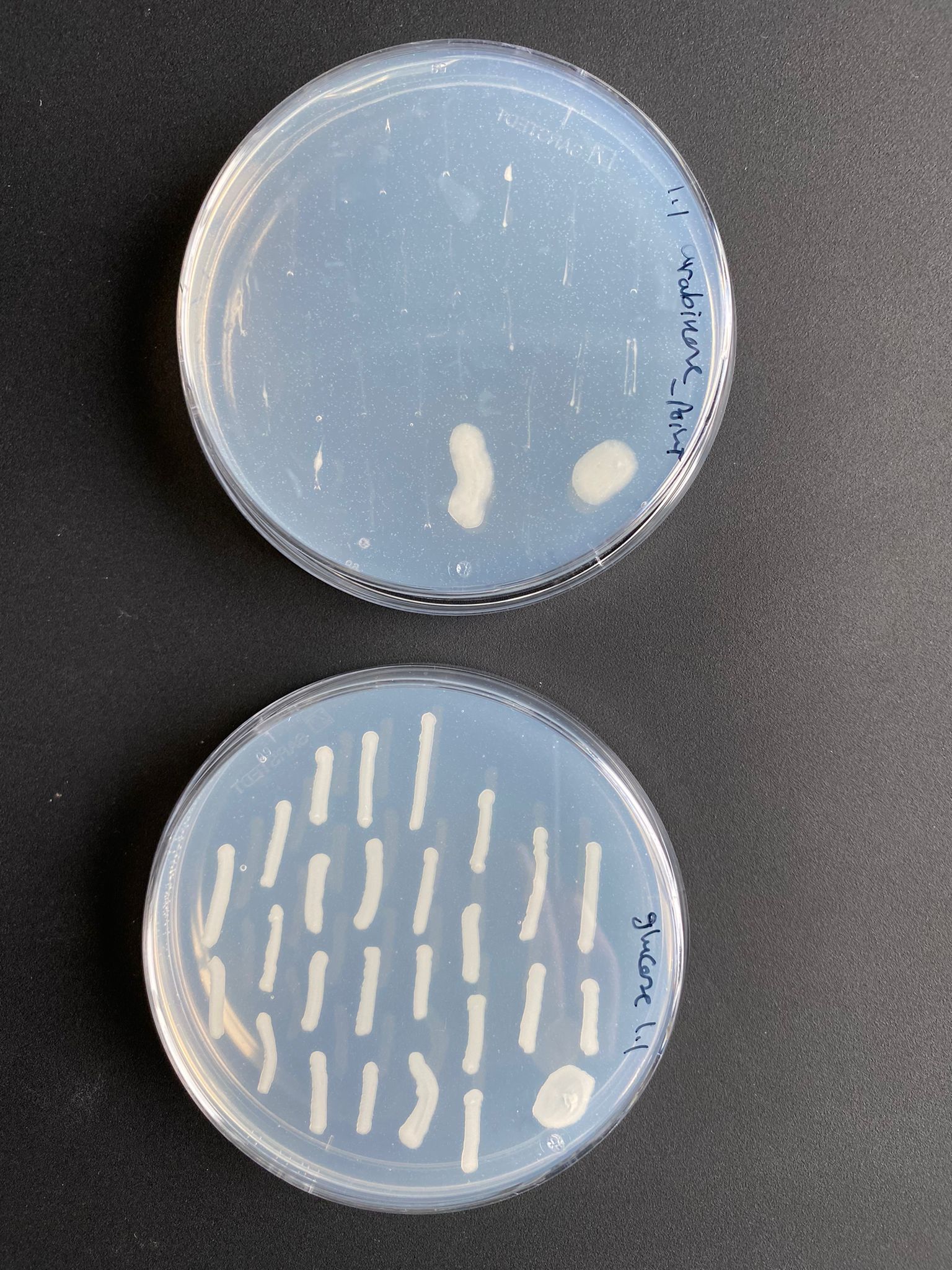

Supplement: Supplementary file 6 — Supplementary Data 4 [file 42003_2025_8934_MOESM6_ESM.zip › Supplementary Data 4/pointmutation/araA_pointmut/araA_pointmut_1.jpeg]

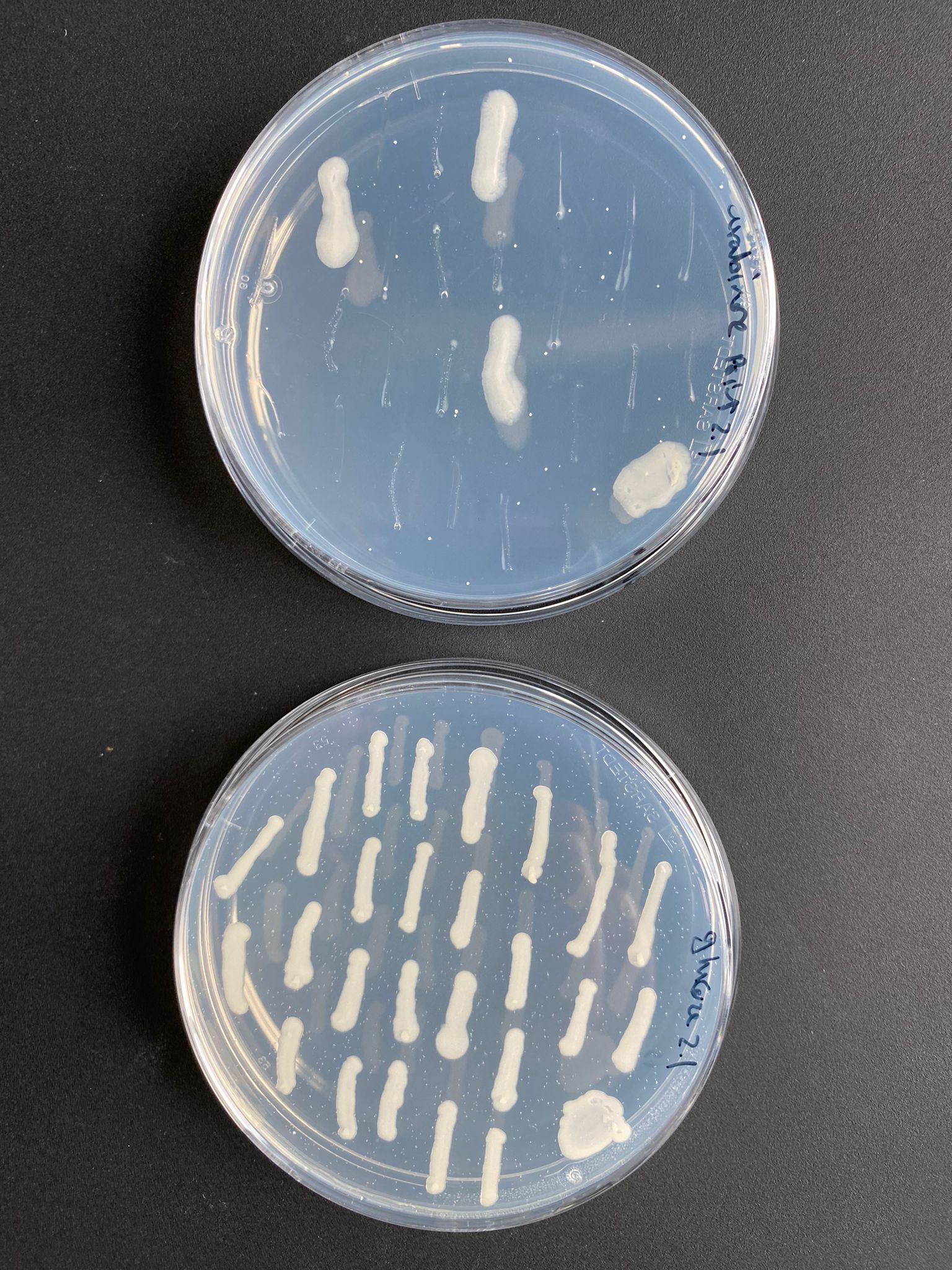

Supplement: Supplementary file 6 — Supplementary Data 4 [file 42003_2025_8934_MOESM6_ESM.zip › Supplementary Data 4/pointmutation/araA_pointmut/araA_pointmut_2.jpeg]

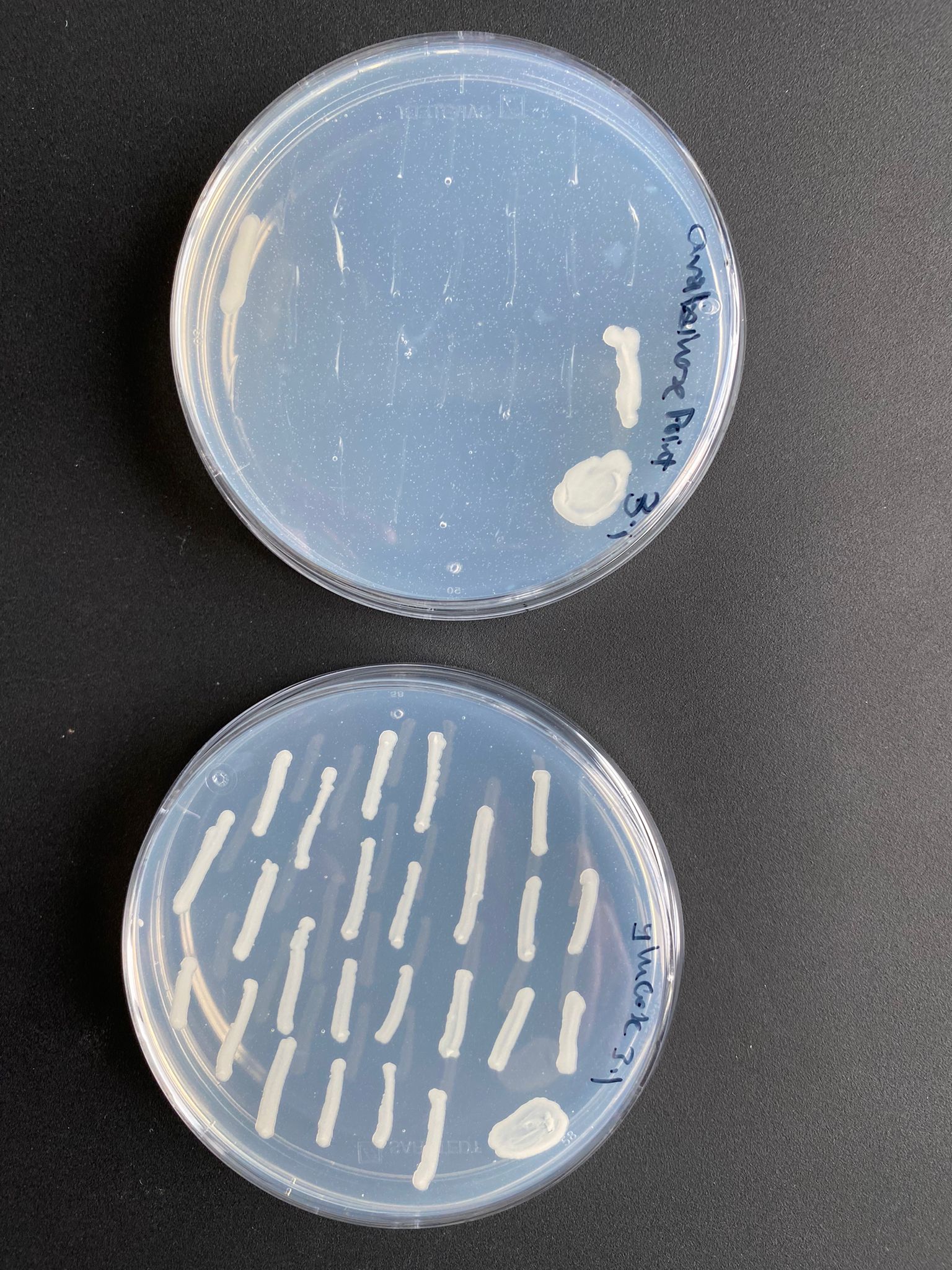

Supplement: Supplementary file 6 — Supplementary Data 4 [file 42003_2025_8934_MOESM6_ESM.zip › Supplementary Data 4/pointmutation/araA_pointmut/araA_pointmut_3.jpeg]

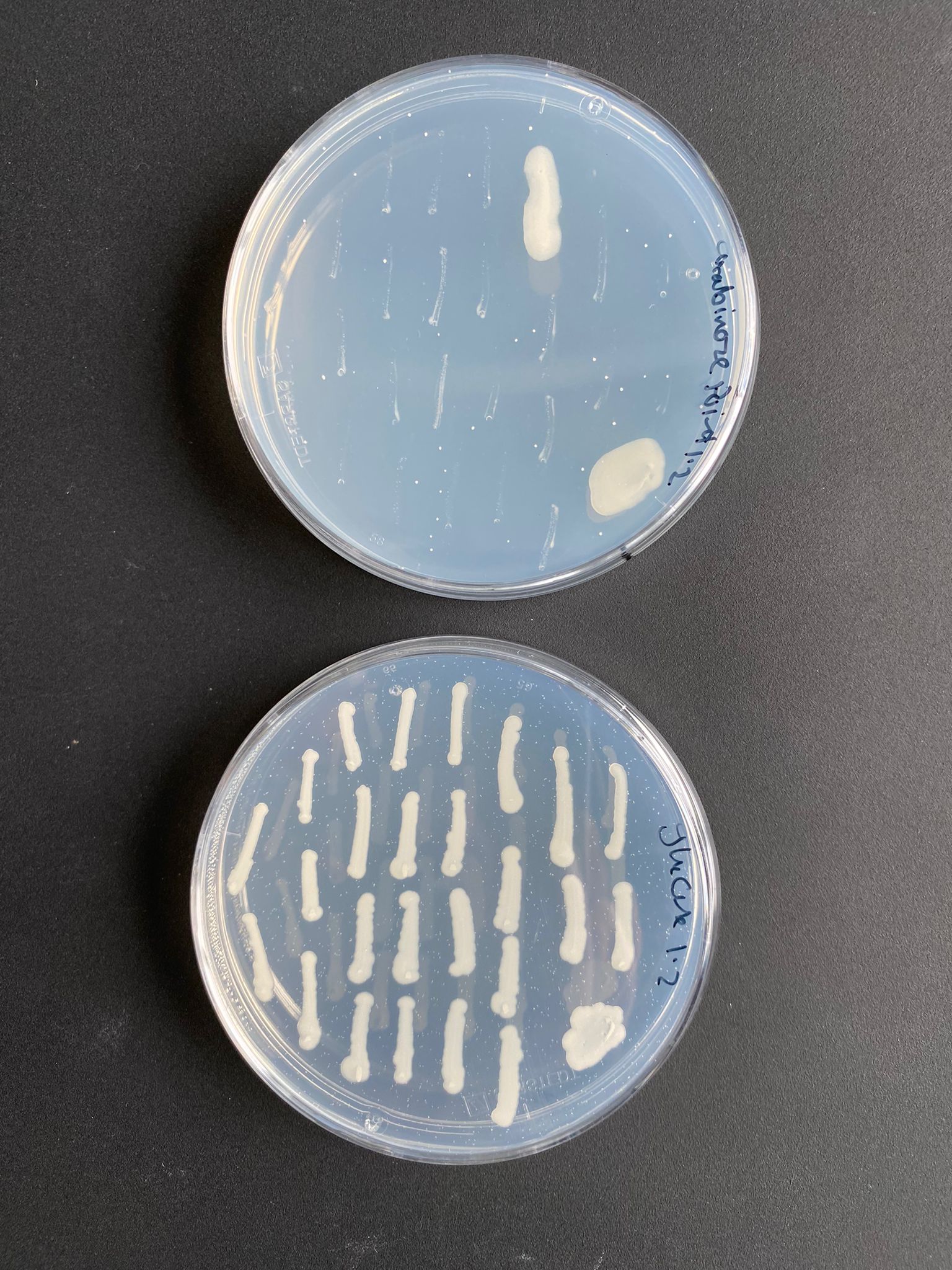

Supplement: Supplementary file 6 — Supplementary Data 4 [file 42003_2025_8934_MOESM6_ESM.zip › Supplementary Data 4/pointmutation/araA_pointmut/araA_pointmut_4.jpeg]

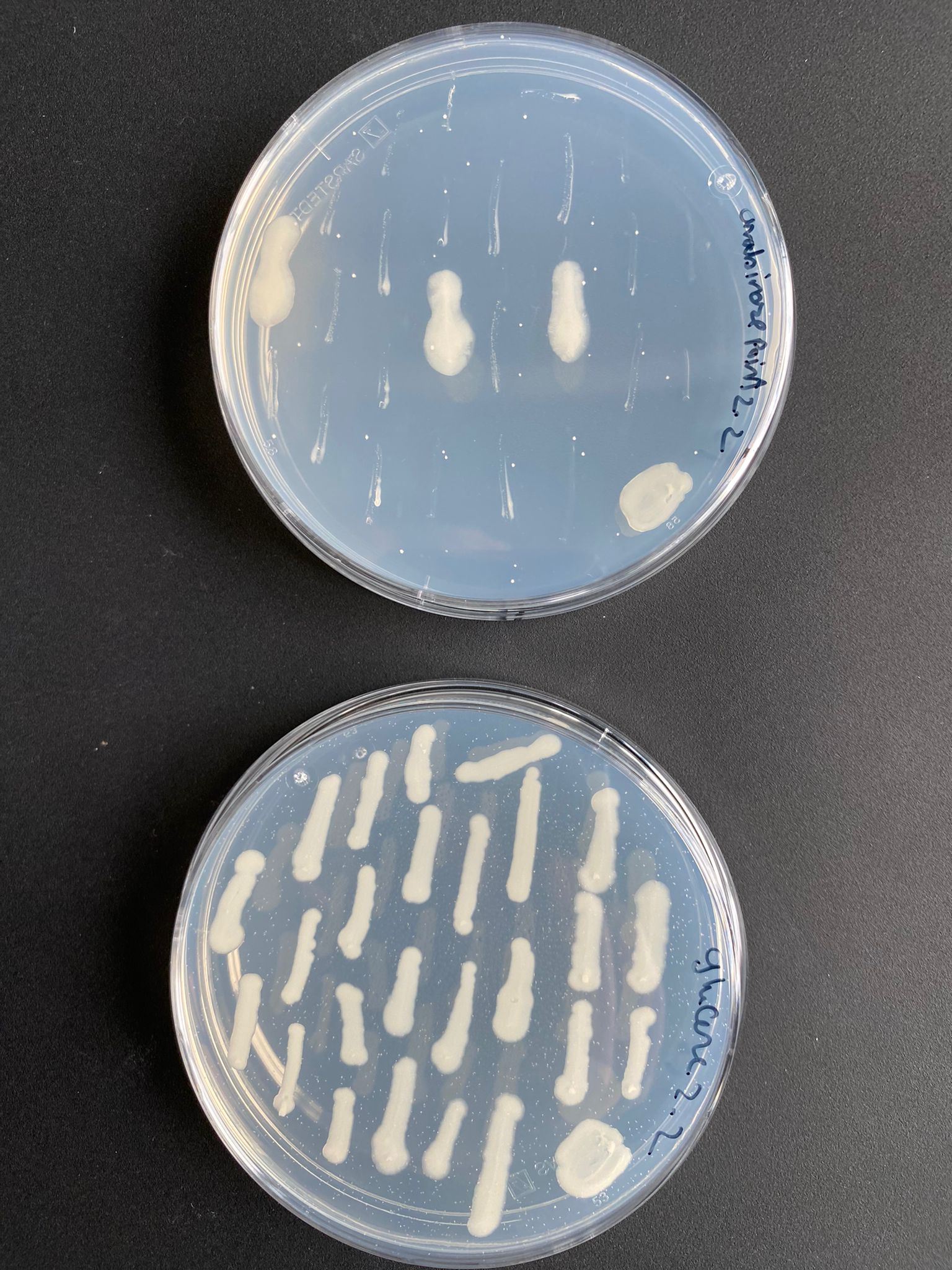

Supplement: Supplementary file 6 — Supplementary Data 4 [file 42003_2025_8934_MOESM6_ESM.zip › Supplementary Data 4/pointmutation/araA_pointmut/araA_pointmut_5.jpeg]

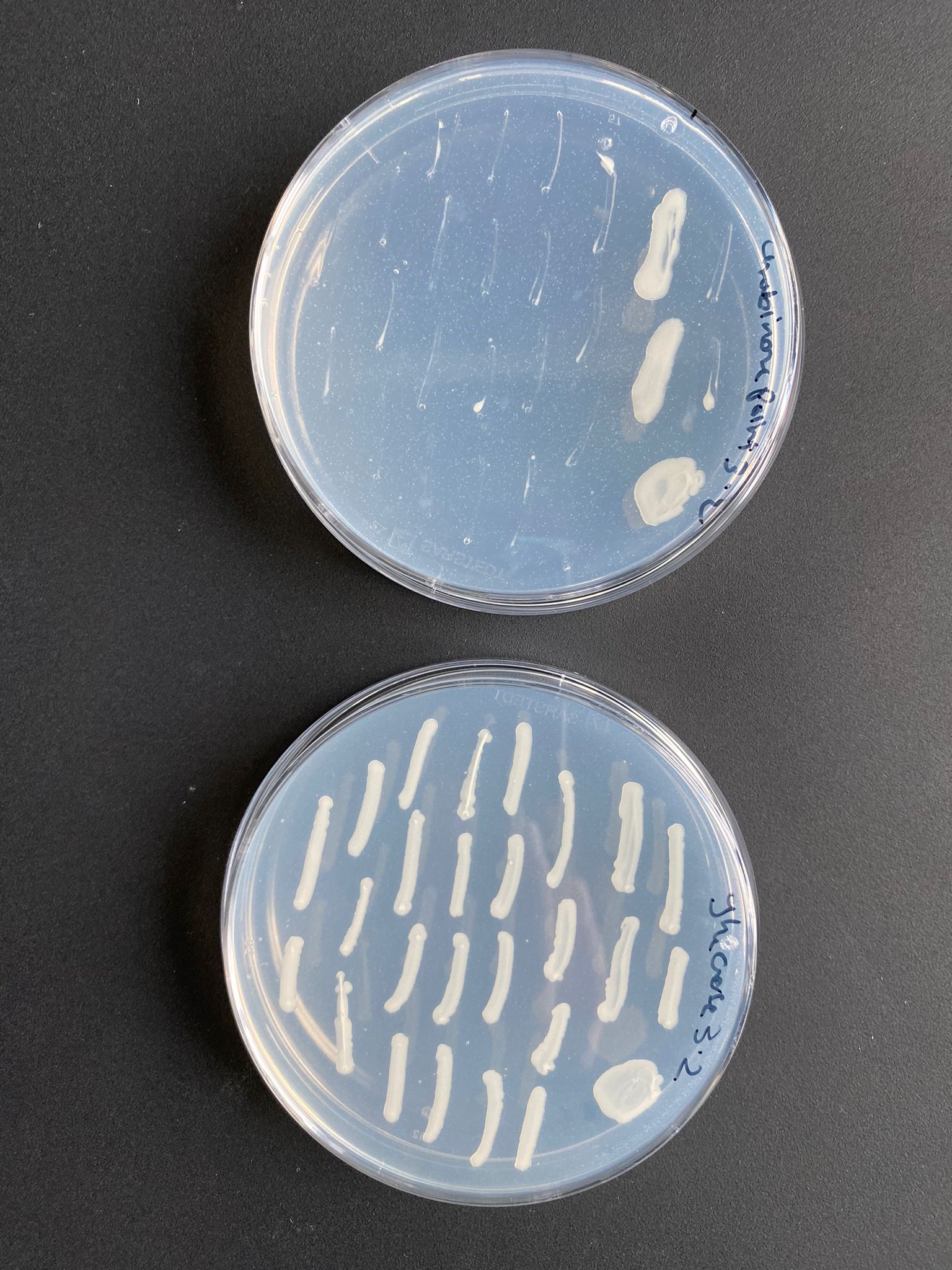

Supplement: Supplementary file 6 — Supplementary Data 4 [file 42003_2025_8934_MOESM6_ESM.zip › Supplementary Data 4/pointmutation/araA_pointmut/araA_pointmut_6.jpeg]

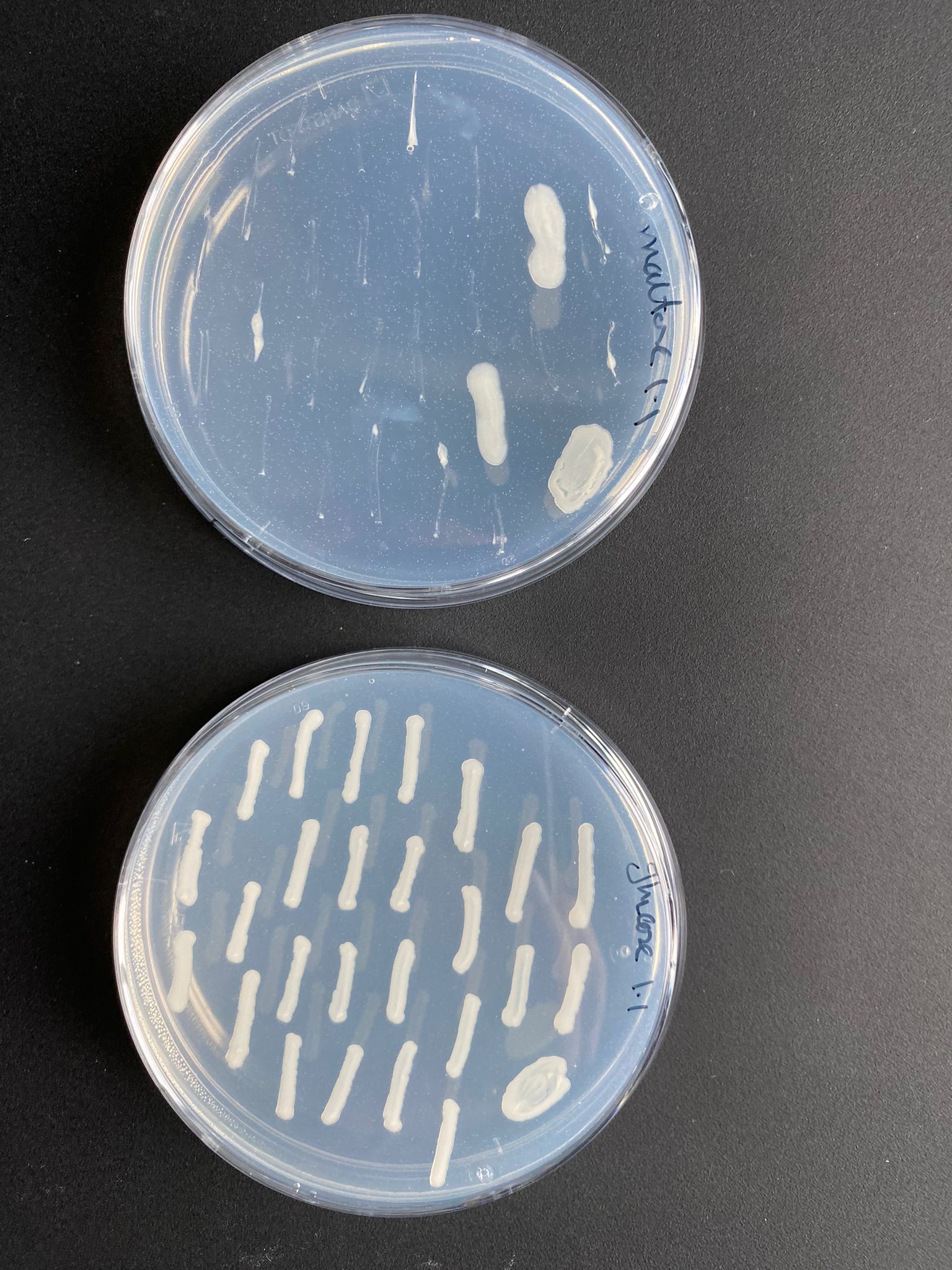

Supplement: Supplementary file 6 — Supplementary Data 4 [file 42003_2025_8934_MOESM6_ESM.zip › Supplementary Data 4/pointmutation/malQ_pointmut/malQ_pointmut_1.jpeg]

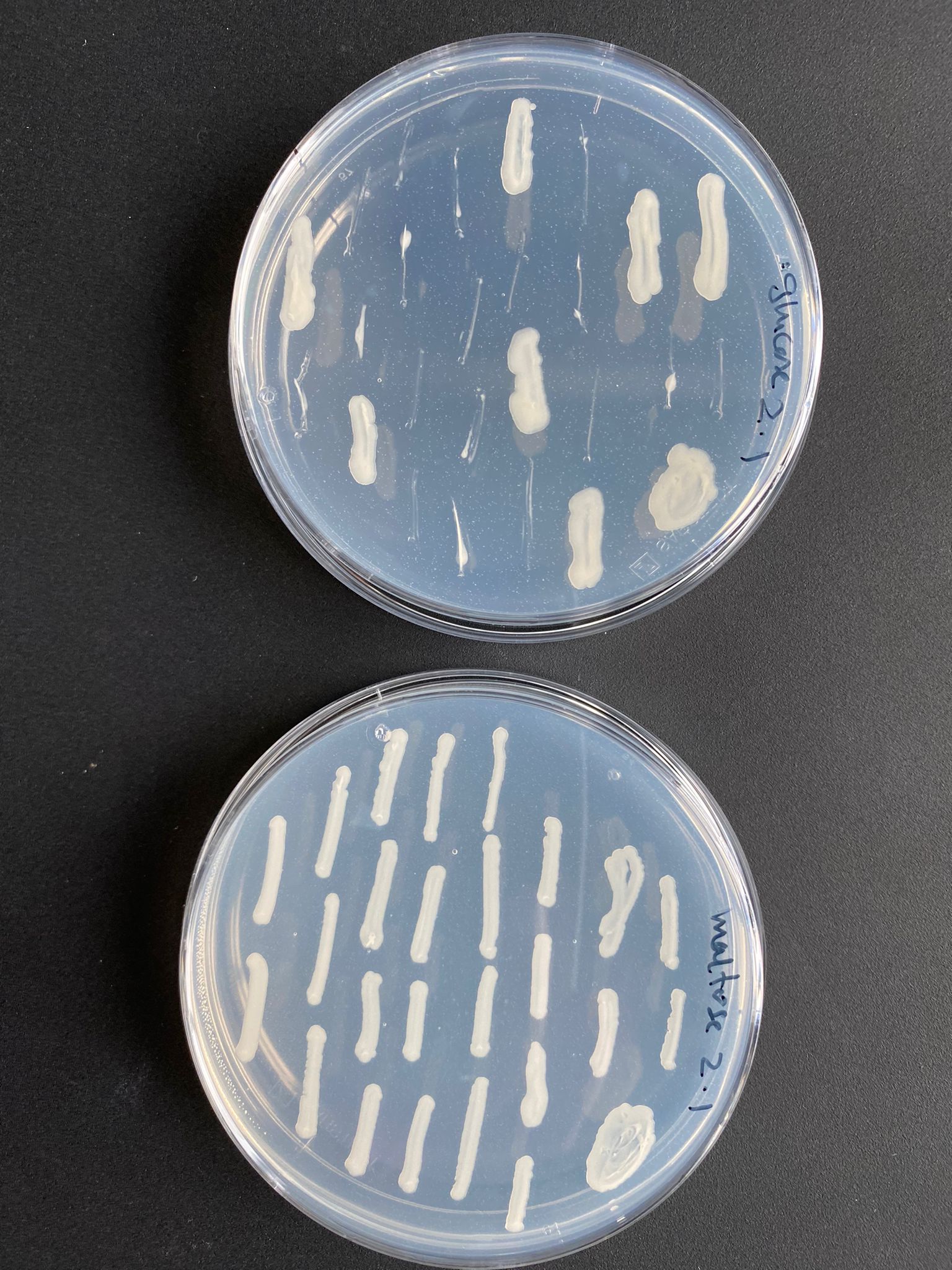

Supplement: Supplementary file 6 — Supplementary Data 4 [file 42003_2025_8934_MOESM6_ESM.zip › Supplementary Data 4/pointmutation/malQ_pointmut/malQ_pointmut_2.jpeg]

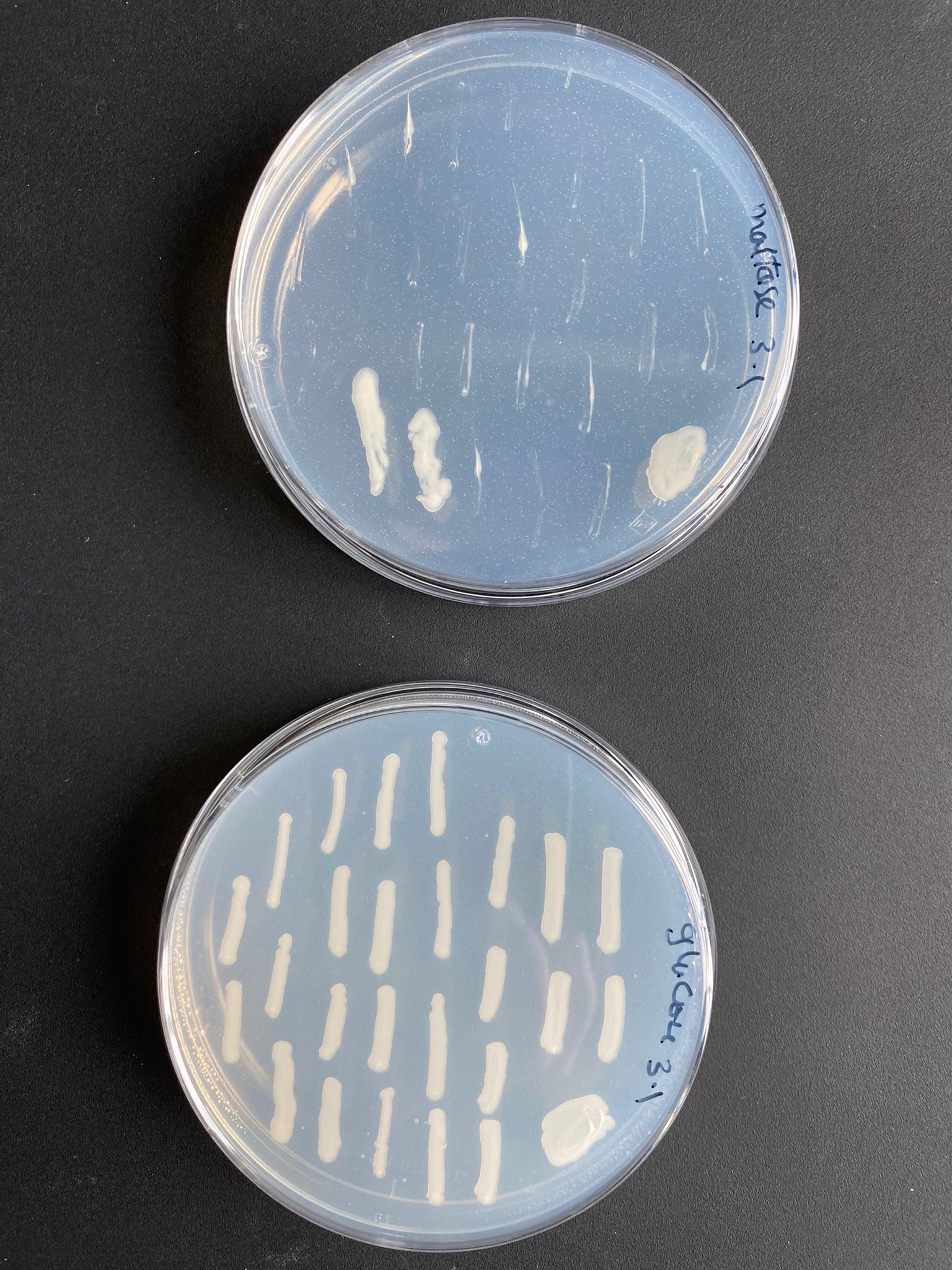

Supplement: Supplementary file 6 — Supplementary Data 4 [file 42003_2025_8934_MOESM6_ESM.zip › Supplementary Data 4/pointmutation/malQ_pointmut/malQ_pointmut_3.jpeg]

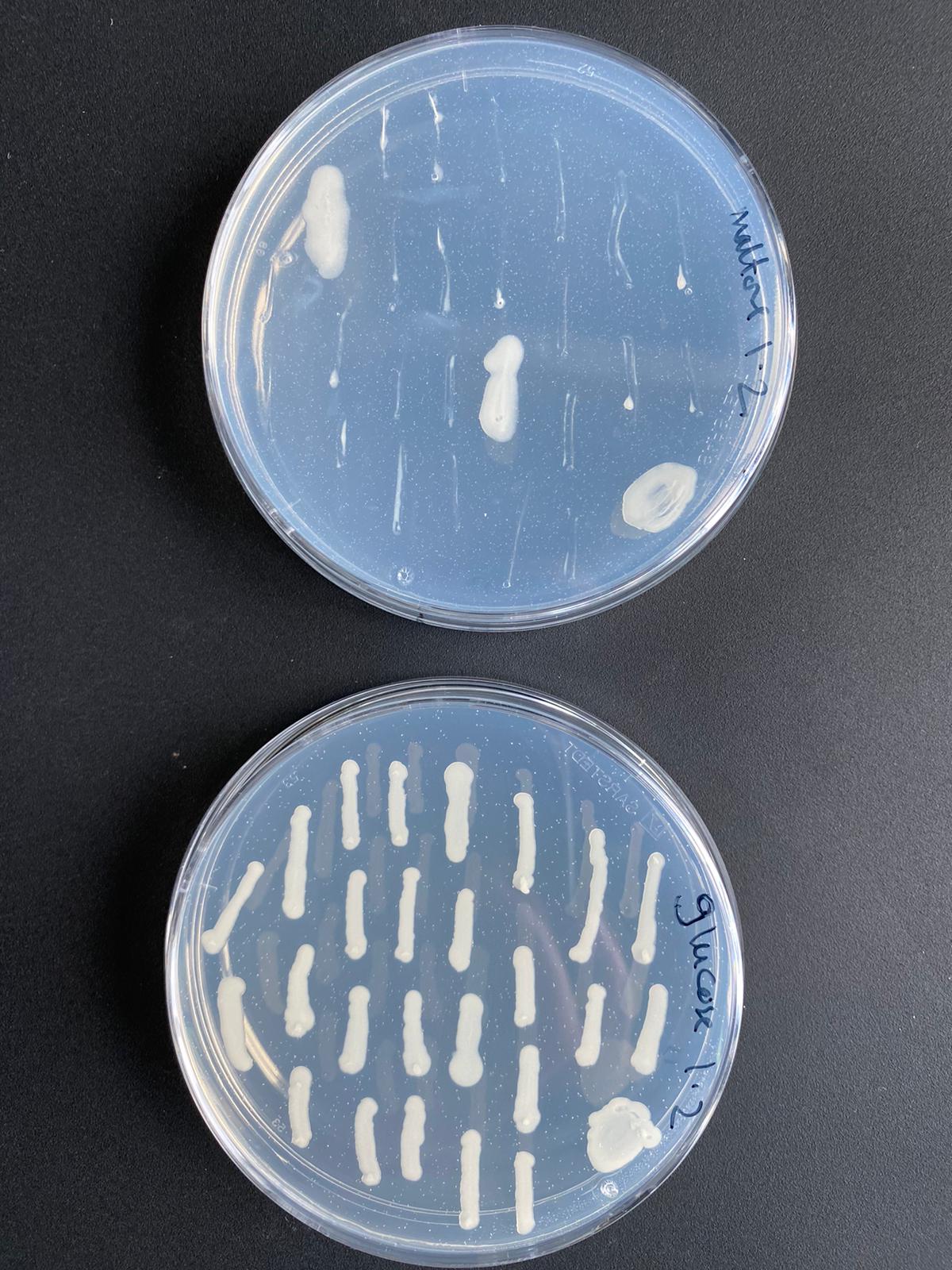

Supplement: Supplementary file 6 — Supplementary Data 4 [file 42003_2025_8934_MOESM6_ESM.zip › Supplementary Data 4/pointmutation/malQ_pointmut/malQ_pointmut_4.jpeg]

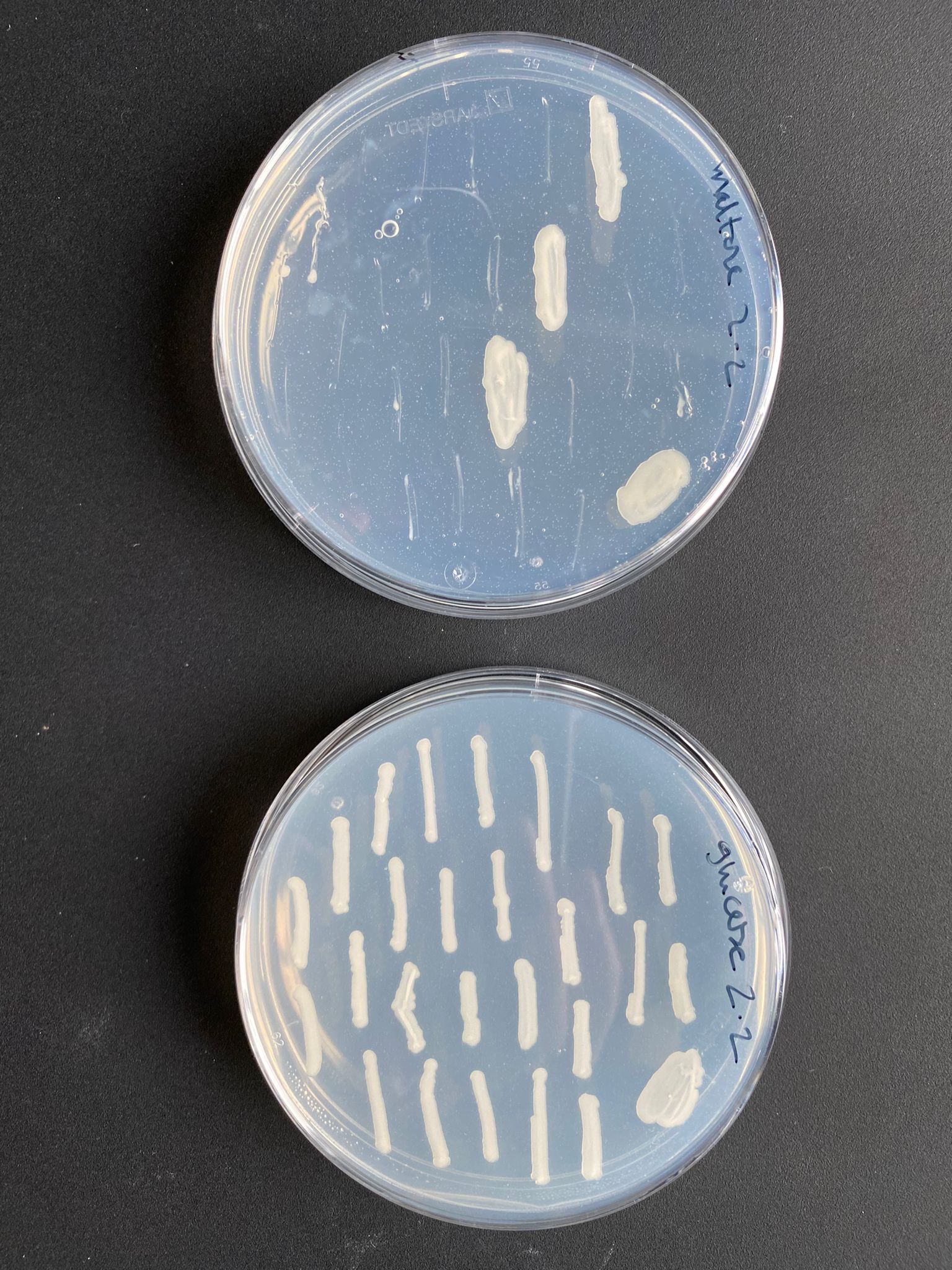

Supplement: Supplementary file 6 — Supplementary Data 4 [file 42003_2025_8934_MOESM6_ESM.zip › Supplementary Data 4/pointmutation/malQ_pointmut/malQ_pointmut_5.jpeg]

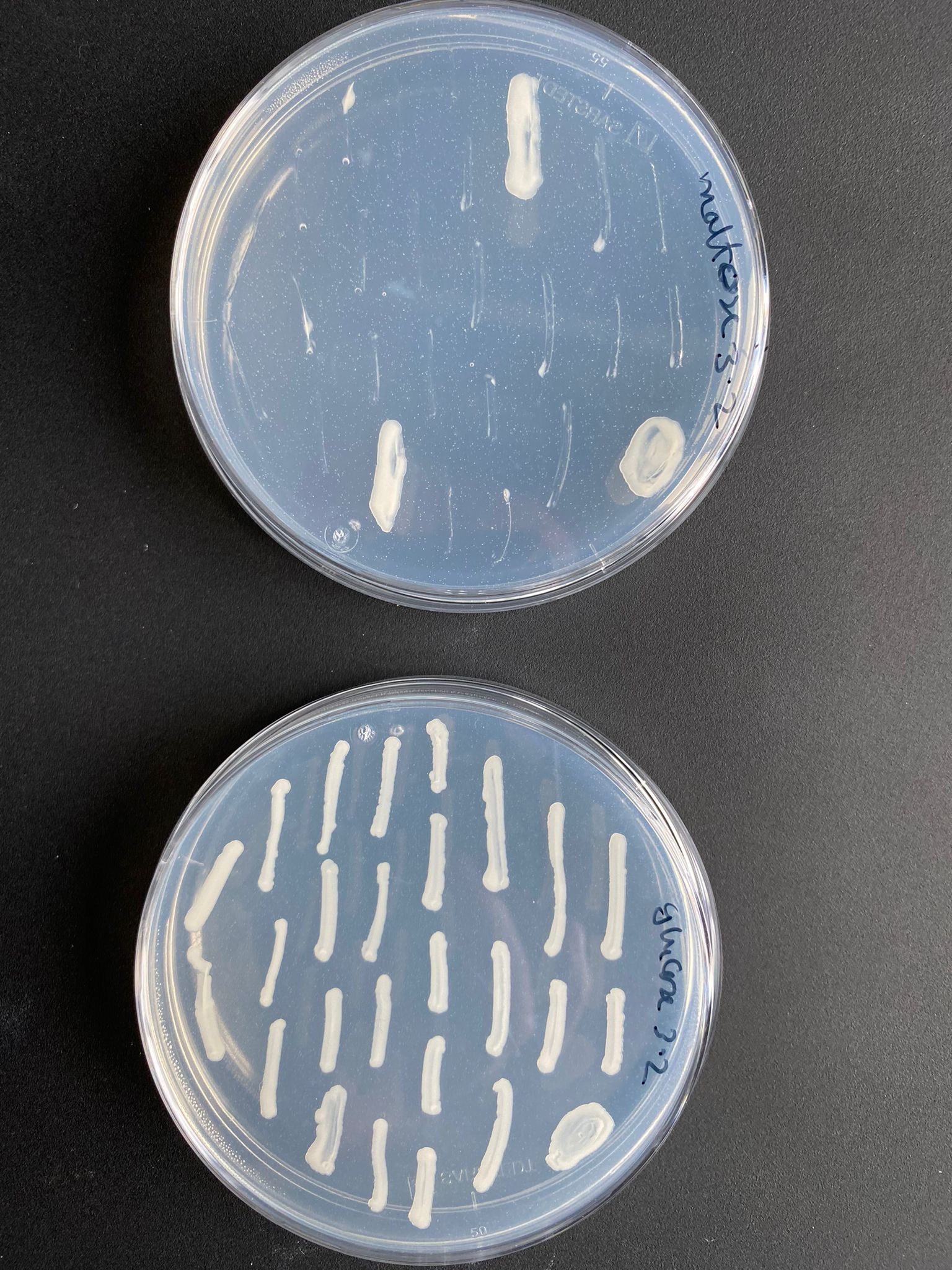

Supplement: Supplementary file 6 — Supplementary Data 4 [file 42003_2025_8934_MOESM6_ESM.zip › Supplementary Data 4/pointmutation/malQ_pointmut/malQ_pointmut_6.jpeg]

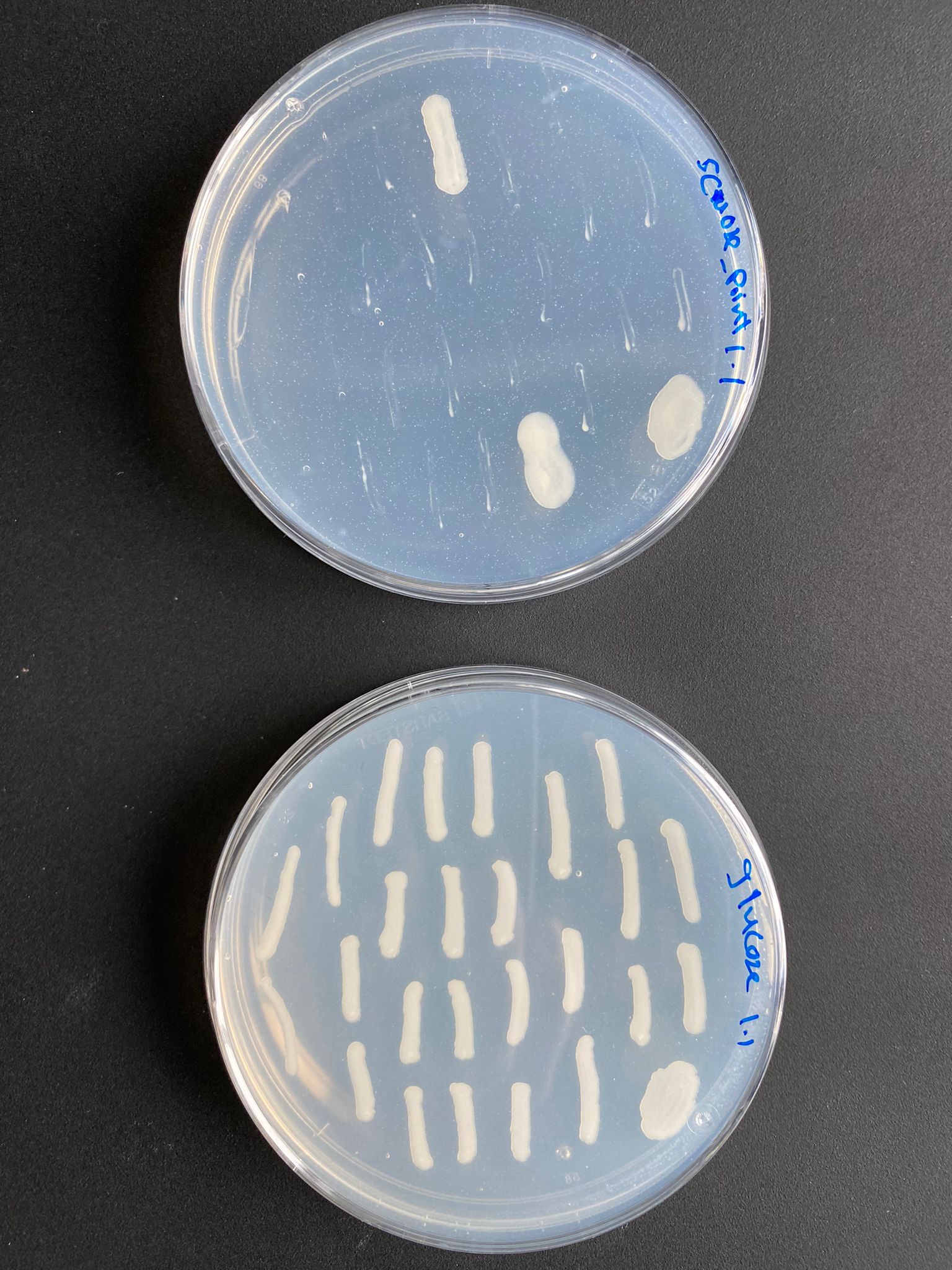

Supplement: Supplementary file 6 — Supplementary Data 4 [file 42003_2025_8934_MOESM6_ESM.zip › Supplementary Data 4/pointmutation/scrK_pointmut/scrK_pointmut_1.jpeg]

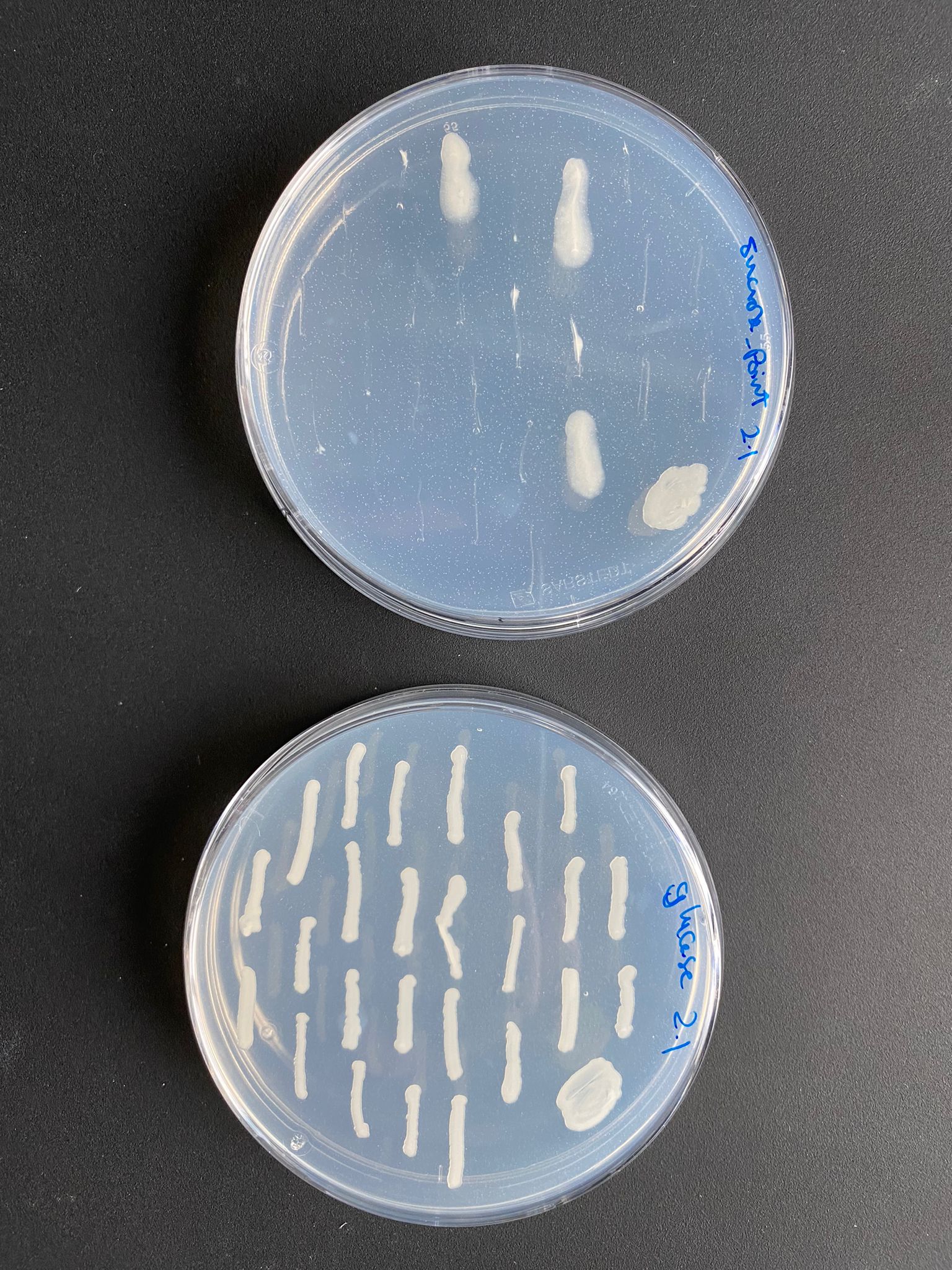

Supplement: Supplementary file 6 — Supplementary Data 4 [file 42003_2025_8934_MOESM6_ESM.zip › Supplementary Data 4/pointmutation/scrK_pointmut/scrK_pointmut_2.jpeg]

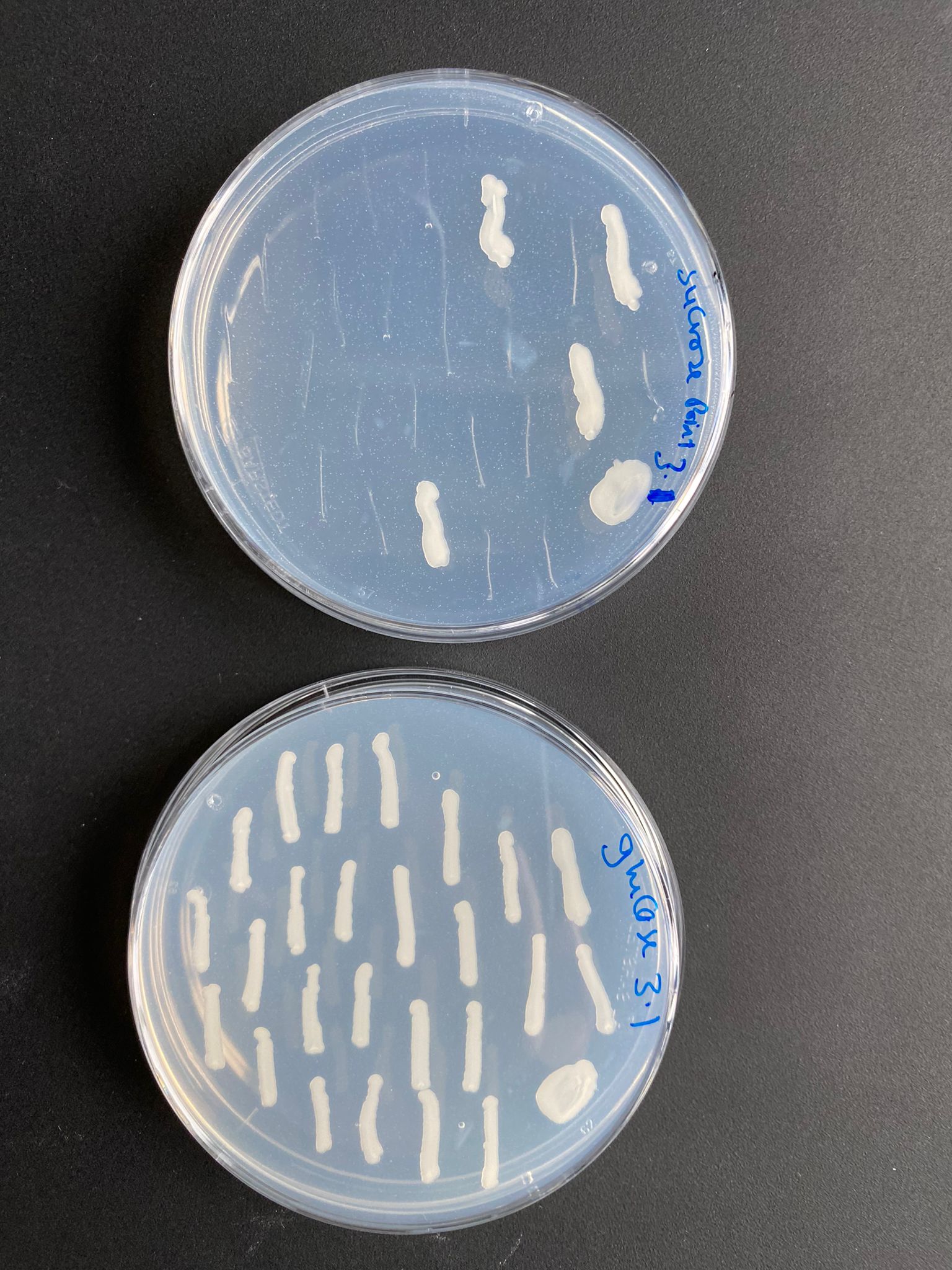

Supplement: Supplementary file 6 — Supplementary Data 4 [file 42003_2025_8934_MOESM6_ESM.zip › Supplementary Data 4/pointmutation/scrK_pointmut/scrK_pointmut_3.jpeg]

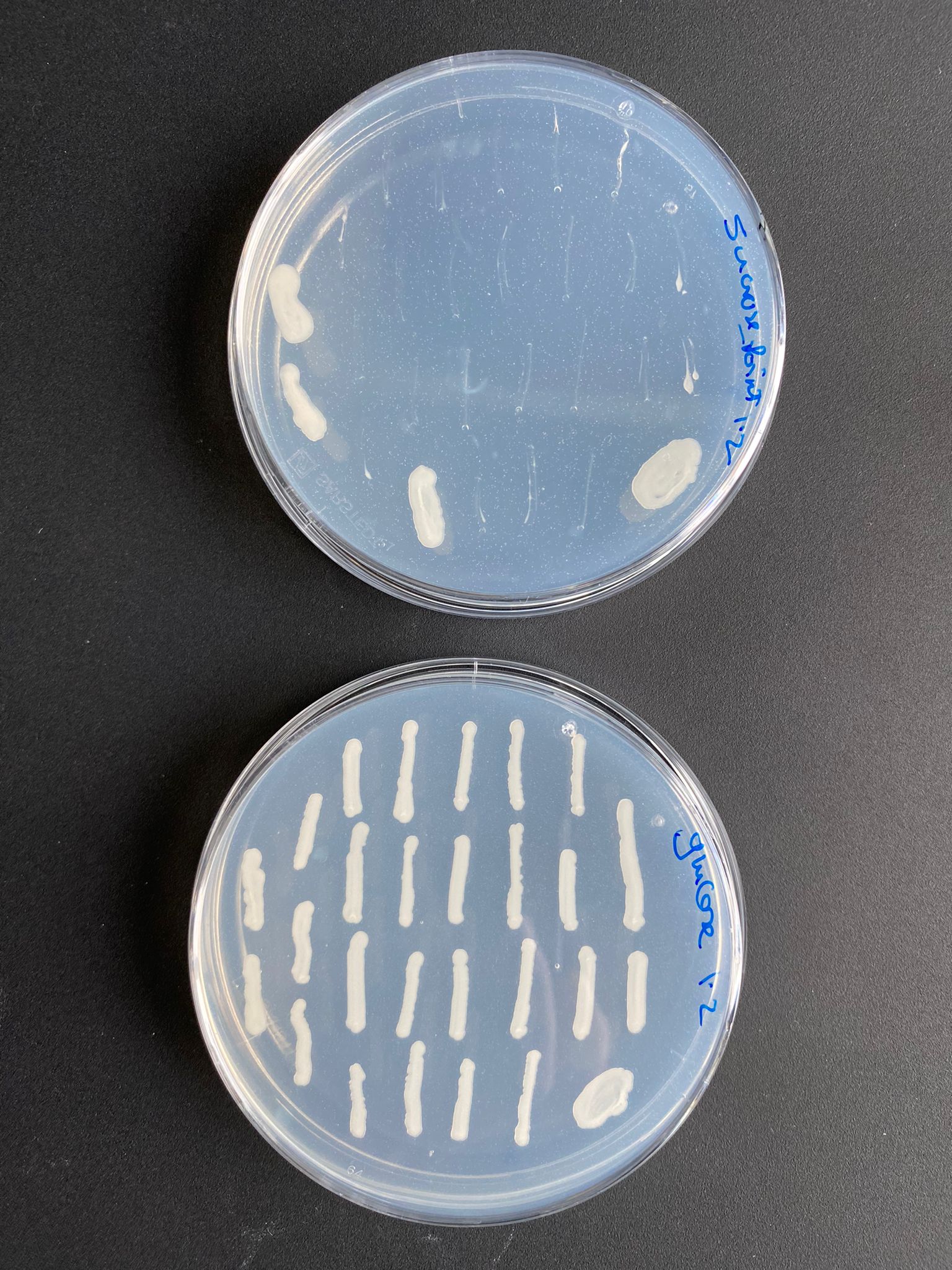

Supplement: Supplementary file 6 — Supplementary Data 4 [file 42003_2025_8934_MOESM6_ESM.zip › Supplementary Data 4/pointmutation/scrK_pointmut/scrK_pointmut_4.jpeg]

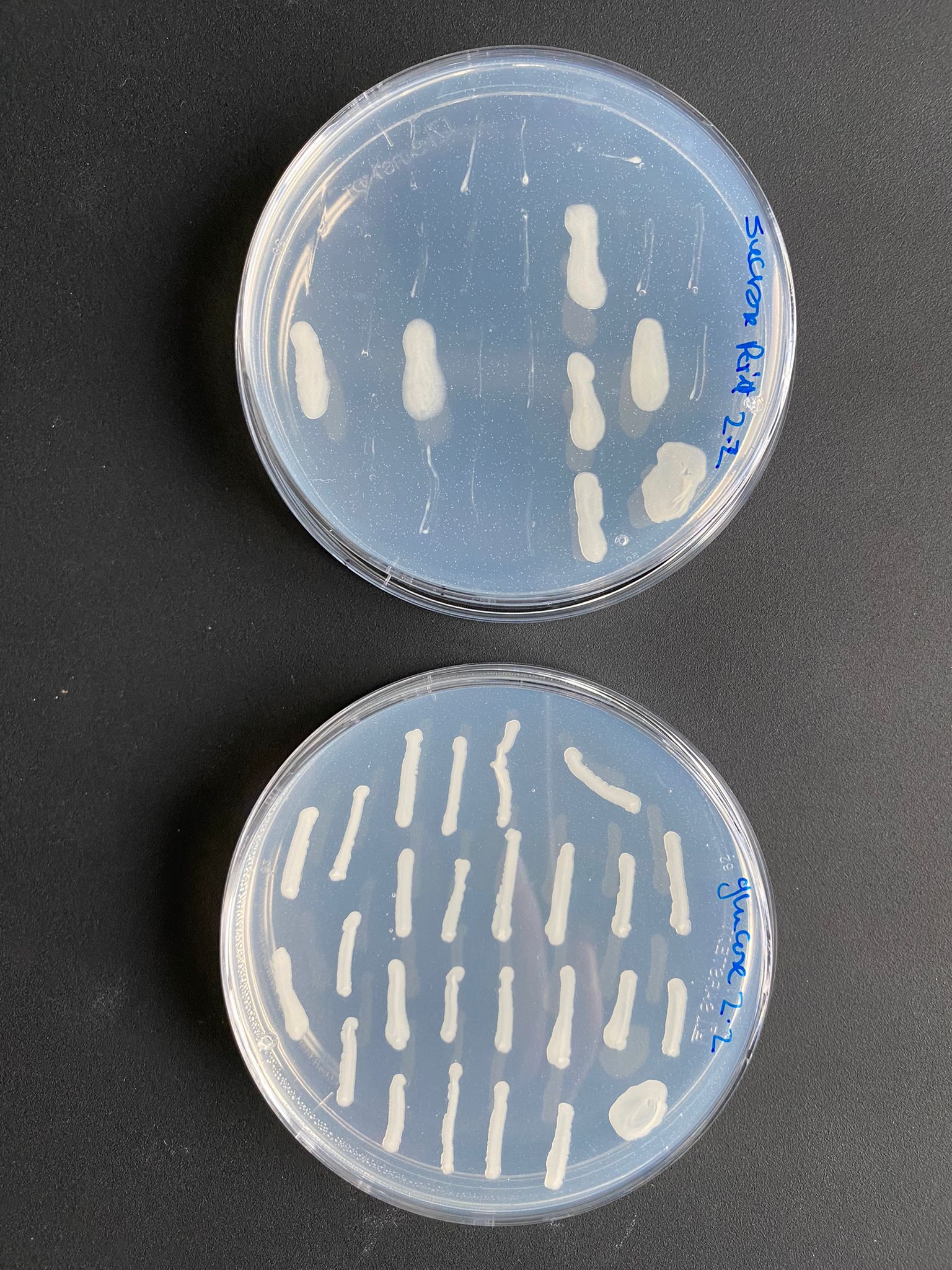

Supplement: Supplementary file 6 — Supplementary Data 4 [file 42003_2025_8934_MOESM6_ESM.zip › Supplementary Data 4/pointmutation/scrK_pointmut/scrK_pointmut_5.jpeg]

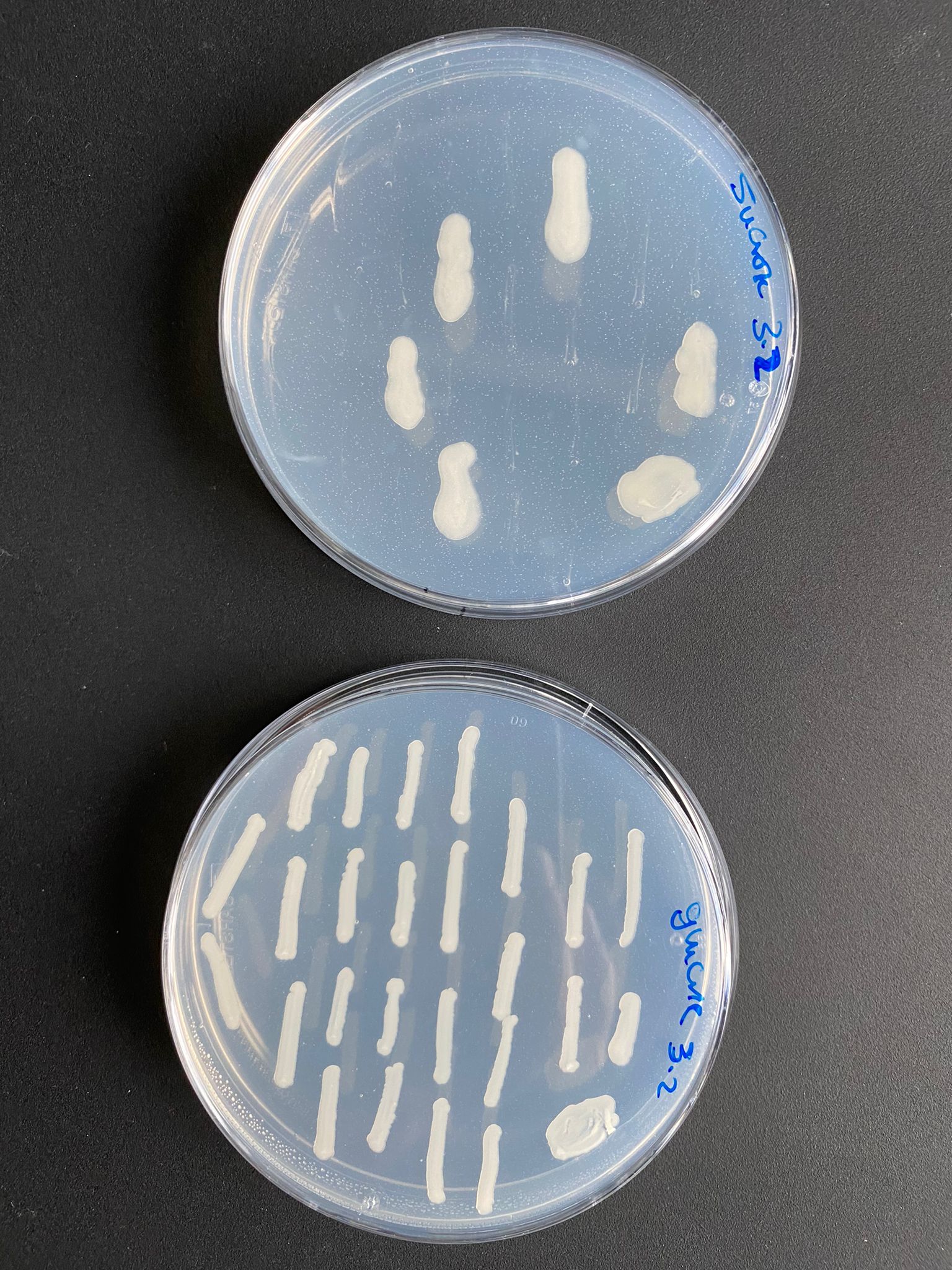

Supplement: Supplementary file 6 — Supplementary Data 4 [file 42003_2025_8934_MOESM6_ESM.zip › Supplementary Data 4/pointmutation/scrK_pointmut/scrK_pointmut_6.jpeg]
