# Supplementary material for: RECKLEEN is a lambda Red/CRISPR-Cas9 based single plasmid platform for enhanced genome editing in Klebsiella pneumoniae
Source: Commun Biol. 2025 Oct 30;8:1509. doi: 10.1038/s42003-025-08934-8 (PMC12575629; doi:10.1038/s42003-025-08934-8)
Supplement: Supplementary file 10 — Reporting Summary [file 42003_2025_8934_MOESM10_ESM.pdf]

nature portfolio

Corresponding author(s): Prof. Dr. Anke Becker

Last updated by author(s): Jul 29, 2025

Reporting Summary

Nature Portfolio wishes to improve the reproducibility of the work that we publish. This form provides structure for consistency and transparency in reporting. For further information on Nature Portfolio policies, see our Editorial Policies and the Editorial Policy Checklist.

Statistics

For all statistical analyses, confirm that the following items are present in the figure legend, table legend, main text, or Methods section.

n/a	Confirmed	
☐	☒	The exact sample size (*n*) for each experimental group/condition, given as a discrete number and unit of measurement
☐	☒	A statement on whether measurements were taken from distinct samples or whether the same sample was measured repeatedly
☒	☐	The statistical test(s) used AND whether they are one- or two-sided<br>*Only common tests should be described solely by name; describe more complex techniques in the Methods section.*
☒	☐	A description of all covariates tested
☒	☐	A description of any assumptions or corrections, such as tests of normality and adjustment for multiple comparisons
☐	☒	A full description of the statistical parameters including central tendency (e.g. means) or other basic estimates (e.g. regression coefficient) AND variation (e.g. standard deviation) or associated estimates of uncertainty (e.g. confidence intervals)
☒	☐	For null hypothesis testing, the test statistic (e.g. F, t, r) with confidence intervals, effect sizes, degrees of freedom and P value noted<br>*Give P values as exact values whenever suitable.*
☒	☐	For Bayesian analysis, information on the choice of priors and Markov chain Monte Carlo settings
☒	☐	For hierarchical and complex designs, identification of the appropriate level for tests and full reporting of outcomes
☒	☐	Estimates of effect sizes (e.g. Cohen's *d*, Pearson's *r*), indicating how they were calculated

Our web collection on statistics for biologists contains articles on many of the points above.

Software and code

Policy information about availability of computer code

Data collection	No software was used for data collection
Data analysis	Data are shown as mean values ± SEM for at least three biologically independent experiments. Prism 8.4.3 (GraphPad, La Jolla,USA) was used.

For manuscripts utilizing custom algorithms or software that are central to the research but not yet described in published literature, software must be made available to editors and reviewers. We strongly encourage code deposition in a community repository (e.g. GitHub). See the Nature Portfolio guidelines for submitting code & software for further information.

Data

Policy information about availability of data

All manuscripts must include a data availability statement. This statement should provide the following information, where applicable:

- Accession codes, unique identifiers, or web links for publicly available datasets
- A description of any restrictions on data availability
- For clinical datasets or third party data, please ensure that the statement adheres to our policy

The maps of the RECKLEEN plasmids are available as Supplementary Data 1. Plasmids will be submitted to Addgene (plasmid IDs 233456 to 233465) and are available from the authors upon reasonable request.
Descriptions of plasmid assemblies are provided in Supplementary table S1. Strains that have been used and constructed in this study are provided in Supplementary table S2. Sequences of all oligonucleotides used in this study are provided in Supplementary Tables S3 – S6. All supplementary tables are available in

Supplementary Data 2. Data used to generate the figures shown in this publication is provided as Supplementary Data 3 – 7. Bacterial sequencing data generated in this study have been deposited in the BioStudies database under the accession number E-MTAB-15474. Any other relevant data is available from the corresponding author upon reasonable request.

Research involving human participants, their data, or biological material

Policy information about studies with human participants or human data. See also policy information about sex, gender (identity/presentation), and sexual orientation and race, ethnicity and racism.

Reporting on sex and gender	N/A
Reporting on race, ethnicity, or other socially relevant groupings	N/A
Population characteristics	N/A
Recruitment	N/A
Ethics oversight	N/A

Note that full information on the approval of the study protocol must also be provided in the manuscript.

Field-specific reporting

Please select the one below that is the best fit for your research. If you are not sure, read the appropriate sections before making your selection.

☒ Life sciences ☐ Behavioural & social sciences ☐ Ecological, evolutionary & environmental sciences

For a reference copy of the document with all sections, see nature.com/documents/nr-reporting-summary-flat.pdf

Life sciences study design

All studies must disclose on these points even when the disclosure is negative.

Sample size	No statistical method was used to predetermine sample size. Quantitative experiments were performed with at least three replicates. Three biological replicates were used, defined as three colonies from the electroporation of the RECKLEEN plasmid, to test for detrimental mutations incorporated during strain preparation, and some experiments were repeated twice on different days to confirm reproducibility between experiments.
Data exclusions	No data were excluded
Replication	Replication was ensured by performing all quantitative experiments at least three times on three different days.
Randomization	Randomization is not required as no samples were allocated into experimental groups
Blinding	Blinding was not required as no samples were allocated into experimental groups

Reporting for specific materials, systems and methods

We require information from authors about some types of materials, experimental systems and methods used in many studies. Here, indicate whether each material, system or method listed is relevant to your study. If you are not sure if a list item applies to your research, read the appropriate section before selecting a response.

Materials & experimental systems

n/a	Involved in the study	
☒	☐	Antibodies
☒	☐	Eukaryotic cell lines
☒	☐	Palaeontology and archaeology
☒	☐	Animals and other organisms
☒	☐	Clinical data
☒	☐	Dual use research of concern
☒	☐	Plants

Methods

n/a	Involved in the study	
☒	☐	ChIP-seq
☒	☐	Flow cytometry
☒	☐	MRI-based neuroimaging

Plants

Seed stocks	N/A
Novel plant genotypes	N/A
Authentication	N/A
